# Plasma Proteome Signature for Leukocyte Telomere Length and Its Link to Abdominal Aortic Aneurysm

**DOI:** 10.1111/jcmm.71047

**Published:** 2026-02-12

**Authors:** Aixin Li, Thomas R. Austin, Brian T. Steffen, Ingrid Jacobson, Jiaqi Xie, Nathan Pankratz, John A. Lane, Annette Fitzpatrick, Joshua C. Bis, Dan E. Arking, Thomas Mosley, Sanaz Sedaghat, James S. Pankow, Pamela L. Lutsey, Weihua Guan, Weihong Tang

**Affiliations:** ^1^ Division of Epidemiology and Community Health, School of Public Health University of Minnesota Minneapolis Minnesota USA; ^2^ Cardiovascular Health Research Unit, Department of Medicine University of Washington Seattle Washington USA; ^3^ Division of Computational Health Sciences, Department of Surgery University of Minnesota Medical School Minneapolis Minnesota USA; ^4^ McKusick‐Nathans Institute, Department of Genetic Medicine Johns Hopkins University School of Medicine Baltimore Maryland USA; ^5^ Division of Molecular Pathology and Genomics University of Minnesota Minneapolis Minnesota USA; ^6^ Departments of Family Medicine and Epidemiology, Schools of Medicine and Public Health University of Washington Seattle Washington USA; ^7^ Department of Medicine, Memory Impairment and Neurodegenerative Dementia (MIND) Center University of Mississippi Jackson Mississippi USA; ^8^ Division of Biostatistics and Health Data Science, School of Public Health University of Minnesota Minneapolis Minnesota USA

**Keywords:** abdominal aortic aneurysm, mendelian randomization, proteomics, SNP, telomere length

## Abstract

Shorter leukocyte telomere length (LTL) is an aging biomarker and risk factor for aging‐related diseases, including abdominal aortic aneurysm (AAA). This study aimed to identify plasma proteins causally associated with LTL and investigate their roles in linking LTL to AAA. A proteomics analysis was conducted for LTL and a polygenic risk score (PRS) for LTL using 4955 plasma proteins by SomaScan in self‐identified White participants (*N* = 7587–8055) from the Atherosclerosis Risk in Communities (ARIC) study. Replications were evaluated in self‐identified Black participants (*N* = 1668–2094) from ARIC and White participants (*N* = 2333–2431) from the Cardiovascular Health Study (CHS). Mendelian randomization (MR) analysis assessed causality between LTL and proteins. Survival and mediation analyses explored protein‐mediated associations between LTL and AAA risk. In ARIC White participants, 15 unique proteins were identified for LTL or LTL PRS. Three LTL‐associated proteins (MZB1, PLOD3, COL28A1) replicated in ARIC Black participants, and six (TNFRSF17, MZB1, CHL1, GDF15, THPO and PLOD3) in CHS White participants. Three proteins associated with LTL PRS (THPO, GP1Bα, PEAR1) replicated in CHS White participants. MR analysis supported causal associations between LTL and five proteins (KDR, TNFRSF17, GDF15, ST3GAL6, CHL1) with all except GDF15 being novel to LTL. LTL was associated with AAA risk (HR = 0.873, 95% CI: 0.803–0.950), with GDF15 mediating 12.4% of this association (*p* = 0.028). We identified five proteins causally influenced by LTL and highlighted GDF15 as a mediator linking LTL to AAA risk, offering novel insights into aging biology and AAA pathogenesis.

**Abbreviations**
AAA: Abdominal Aortic Aneurysm
ARIC: Atherosclerosis Risk in Communities
CHS: Cardiovascular Health Study
eGFR: Estimated Glomerular Filtration Rate
GWAS: Genome‐Wide Association Study
IV: Instrumental Variable
LTL: Leukocyte telomere length
MR: Mendelian Randomization
PRESSO: Pleiotropy Residual Sum and Outlier
PRS: Polygenic Risk Score
SNP: Single Nucleotide Polymorphism
WGS: Whole Genome Sequencing

## 1 Introduction

Telomeres are repeating DNA sequences located at the end of chromosomes that protect chromosomal regions from recombination, degradation, and the loss of essential DNA sequences during replication [1]. With each cell division (e.g., as an individual ages), telomeres get shorter in their nucleotide repeats. Thanks to the accessibility of leukocytes, leukocyte telomere length (LTL) is commonly used to study telomere length dynamics [2]. Numerous studies have associated LTL with aging [3], age‐related diseases [4, 5, 6], and certain cancers [7, 8, 9]. However, the underlying biological mechanisms mediating telomere length and age‐related diseases are not fully elucidated. While a few studies have linked candidate proteins with LTL [10, 11, 12], to the best of our knowledge, no large‐scale proteomics study has been conducted for LTL.

Abdominal aortic aneurysm (AAA), characterised by a localised enlargement of the abdominal aorta, is an important age‐related disease and can lead to life‐threatening rupture if left untreated [13, 14]. While both animal and Mendelian randomization (MR) studies suggest a causal role of LTL in AAA development [15, 16], and certain proteins have been associated with AAA [17], the longitudinal relationship between LTL and AAA has not been well‐characterised in large prospective studies with long‐term follow‐up. Moreover, the underlying biological mechanisms mediating the association of LTL with AAA are poorly understood.

To improve the understanding of biological mechanisms related to telomere length, we conducted a large‐scale analysis to identify the proteomic signatures of LTL and LTL polygenic risk scores (PRS) [18] in the Atherosclerosis Risk in Communities (ARIC) Study cohort. We derived the LTL PRS based on a published genome‐wide association study (GWAS) [19]. We then replicated the LTL‐protein associations in the Cardiovascular Health Study (CHS).

To further investigate the causal link between LTL and identified proteins, we conducted a bidirectional Mendelian randomization (MR) analysis [20]. MR uses genetic variants associated with LTL (forward causality) or proteins (reverse causality) as instrumental variables (IVs). When certain assumptions are met, estimated exposure/outcome associations are free from social and environmental confounding and, therefore, provide evidence of causality [20]. Additionally, we conducted a prospective cohort study between LTL and AAA in ARIC followed by a mediation analysis to identify proteins that might mediate the pathway from LTL to AAA. The analysis workflow of the study is shown in Figure S1.

## 2 Methods

### 2.1 Study Populations

ARIC is an ongoing longitudinal cohort study that initially enrolled 15,792 men and women aged 45–64 in four U.S. communities (Washington County, MD; Forsyth County, NC; the northwestern suburbs of Minneapolis, MN; and Jackson, MS) between 1987 and 1989 [21]. Information on cardiovascular risk factors, medication history, and behavioural risk factors was collected at visit 1 in 1987–89, with subsequent reexaminations at visit 2 (1990–92) and visit 3 (1993–95), which are included in the present analysis. Several further visits have been conducted as part of the ongoing ARIC study. The present study utilized proteomics data measured in plasma samples collected at ARIC visits 2 and 3. The institutional review board at each field center institution and the Coordinating Center approved the study protocol.

To replicate the proteins identified in ARIC, we examined their associations with LTL in the CHS, a population‐based cohort that enrolled 5888 self‐identified White participants and Black participants aged 65 years or older from four U.S. communities (Forsyth County, NC; Sacramento County, CA; Washington County, MD; and Pittsburgh, PA) in the late 1980s and early 1990s [22]. The CHS was approved by institutional review boards at each of the four field centers and the Coordinating Center.

In both ARIC and CHS, all participants provided informed consent, and genetic analyses were limited to those with available DNA who consented to genetic studies.

### 2.2 Proteomics Measurement and Quality Control in ARIC

Plasma protein data from visit 2 samples were used for the LTL PRS analysis due to larger sample size (compared to the visit 3 samples), and the protein data from visit 3 samples were used for the LTL analysis to reduce reverse causation bias as LTL was measured from visits 1, 2 or 3 samples (only one visit for each sample). Plasma samples were collected per a standardised protocol and stored at −80°C. The relative concentration of plasma proteins was measured using a modified aptamer‐based array (SomaScan version 4.0, SomaLogic Inc., Boulder, CO, USA) [23, 24], and quantified in relative fluorescence units. The protein measurements were standardised and normalised by SomaLogic [25]. A pilot study of SomaScan v3 previously conducted by ARIC in 42 participants, yielding excellent reproducibility indexes: the median coefficient of variation (CV) was 5.0% (quartile 1, quartile 3: 4.1, 6.9), and the median intraclass correlation coefficient, which measures the consistency of repeated protein measurements, was 0.96 (quartile 1, quartile 3: 0.92, 0.98) [25]. Of the 5284 protein measurements in ARIC using SomaScan v4.0, we excluded 289 proteins binding to mouse, contaminants, or non‐proteins; 22 that were flagged as failed; 29 with a Bland–Altman CV (CVba) > 50%; and 11 with variance (on a log scale) < 0.01; thus left 4955 protein SOMAmers remained for our analysis. Each protein value was log2 transformed. For each participant, protein values that were off 5 standard deviations (SD) from the mean were winsorized, and any individual proteins that were off 6 SD from the mean were further excluded based on the updated distribution after the winsorization.

### 2.3 LTL Measurements in ARIC

We used TelSeq [26] to estimate LTL based on ARIC high‐pass WGS data (~38X) measured in DNA samples from ARIC visit 1, 2 or 3 (a single LTL estimate for each participant). The ARIC WGS data, funded by the NHLBI Trans‐Omics for Precision Medicine (TOPMed) program [27] and the NHGRI Centers for Common Disease Genomics (CCDG) [28, 29], were sequenced by a single lab and called jointly by the TOPMed Informatics Research Center to produce a uniform dataset with identical sequencing parameters, variant filtering, genotyping, and sample QC. TelSeq [26] estimates LTL by counting reads containing a fixed number of telomeric repeats, with the number of TTAGGG repeats being set to 12. This count was then normalised to the number of reads with GC content between 48% and 52%. LTL estimates were inverse normalised within read length group and sequencing program (TOPMed vs. CCDG) before being merged together. We inverse‐normalised the LTL values within read length group and sequencing program because it is well documented that large enough batch effects exist between sequencing runs, which influences the telomere length estimates [29]. By applying this approach to a combined data set of 2468 Jackson Heart Study samples and 602 Women's Health Initiative samples, we noticed that the Pearson correlation between the LTL estimates by TelSeq and Southern blot methods increased from a baseline of 0.263 (*p* = 7.4 × 10^−50^) to 0.564 (*p* = 1.7 × 10^−257^) after inverse normalisation within source and read length.

### 2.4 Proteomics and LTL Measurements in CHS

In CHS, proteomic measurements were obtained from plasma samples collected during the 1992–93 examination using the SomaScan 5K (v.4.0) and 7K (v.4.1) platforms that have been described previously [30]. Prior to data delivery, SomaLogic applied adaptive normalisation by maximum likelihood to correct for sample and study variability. Non‐human and deprecated aptamers were removed prior to analyses, and proteomics data were log2‐transformed and standardised.

In CHS, telomere length was measured in stored specimens from the 1992–93 examination as the mean length of terminal restriction fragments in peripheral leukocytes using the Southern blot method [31]. Duplicates in the sample had a Pearson correlation coefficient of 0.97 and an average coefficient of variation of 1.5%. LTL measurements were available for 1053 White participants. Additionally, we estimated LTL based on WGS data, following the same analytical approach used in ARIC. Due to the limited sample size of Black participants, only White participants were included in the CHS replication analysis. There were 1053 White participants for the Southern blot method and 2431 White participants for the WGS method, both with the SomaScan protein data.

### 2.5 LTL Polygenic Risk Score (PRS)

For each participant, the LTL PRS was computed as a weighted sum of the dosages of the alleles that increased LTL from the ARIC GWAS data imputed to the TOPMed reference panel. The details of genotyping, imputation, and quality control procedures for ARIC data have been previously reported; in brief, genome‐wide genotyping was conducted using the Affymetrix 6.0 SNP Array (Affymetrix, Santa Clara, CA, USA) [32]. ARIC investigators performed imputation of variant dosages separately for White participants and Black participants using the TOPMed reference panel (freeze 5b) [27]. To capture population substructure or genetic ancestry, race‐specific principal components (PC) were generated using EIGENSTRAT [33] based on the genotyped data.

The weights on the allele dosages in the PRS calculation were the estimated effect sizes for the corresponding alleles from a published LTL GWAS [19]. This GWAS included 472,174 well‐characterised UK Biobank participants and identified 197 independent sentinel variants associated with LTL, of which 150 were available in the ARIC GWAS imputation dataset. The LTL PRS was computed using these 150 SNPs in our primary analysis.

For SNPs not present in the ARIC dataset, we looked for proxy variants based on linkage disequilibrium (LD) *r* ^2^ > 0.80 in White participants and Black participants separately through the LDlink website [34]. For SNPs where proxies could not be found based on LD *r* ^2^ > 0.80, we lowered the threshold to *r* ^2^ > 0.70. Consequently, nine missing SNPs were replaced with proxy variants, which were added to the calculation of a second LTL PRS that consisted of 159 SNPs. The second PRS was analysed in a sensitivity analysis in both EAs and AAs. Detailed information about the SNPs used in the primary PRS calculations and the proxy SNPs is available in Tables S1 and S2. All the PRS calculations were conducted using PLINK and data processing was conducted using R (version 4.2.2).

The derivation of LTL PRS in CHS is similar to the approach used in ARIC described above except that we computed the PRS using 187 of the 197 variants based on the WGS data from TOPMed. The other 10 variants were not included because of rare frequency (~0.001) or missingness in the WGS data. PRS calculations in CHS were conducted using GenScorePipeline (https://github.com/PankratzLab/GenScorePipeline).

### 2.6 Assessment of Kidney Function

To account for the potential confounding effects of reduced kidney function in the analyses of plasma proteins with telomere length and AAA, we adjusted for the estimated glomerular filtration rate (eGFR) as a covariate in our analyses [35]. The eGFR was calculated in mL/min/1.73 m^2^ using the Chronic Kidney Disease Epidemiology Collaboration (CKD‐EPI) equation, which incorporates serum creatinine and cystatin C measurements [35]. Detailed methodologies pertaining to the serum creatinine and cystatin C measurements are documented in prior studies [36].

### 2.7 AAA Ascertainment

The methodology for AAA ascertainment in ARIC has been previously described [37]. In brief, the process involved annual or semi‐annual telephone interviews with ARIC participants to collect information about any hospitalizations or deaths that occurred since the last contact, and medical records for these reported events were sought. Additionally, ARIC implemented local hospital surveillance to try to capture hospitalizations or deaths. Furthermore, participant records were cross‐referenced with Medicare data from the Centers for Medicare and Medicaid Services (CMS) for 1991 to 2018 to capture additional outpatient or hospital events for participants aged 65 and above. A total of 727 incident clinical AAA events were identified from ARIC visit 1 through December 31, 2019 (December 31, 2017 for Jackson participants). Thoracic, thoracoabdominal, or unspecified aortic aneurysms were not considered as AAA events in this study. We excluded participants who reported prior AAA surgery or aortic angioplasty at visit 1.

### 2.8 Statistical Analysis

#### 2.8.1 Analysis of LTL With Proteins Measured at ARIC Visit 3

To reduce reverse causation bias, proteomics analysis was conducted using LTL measures estimated from samples collected at ARIC visit 1 (1987–89), visit 2 (1990–92) or visit 3 (1993–95) to predict the levels for each of 4955 proteins measured in visit 3 samples. From the initial cohort of 15,792 participants, 14,348 were available at visit 2. We excluded 2716 participants without LTL estimates; 216 participants whose LTL estimates were not from visits 1, 2 or 3; 1659 participants with missing plasma protein measurements or covariate data. For each individual protein, we further excluded participants whose protein levels were off 6 SD from the mean (minimum exclusion *N* = 14). This yielded 8055 White participants in the discovery set and 1668 Black participants in the replication set. The schematic of the sample size determinations is shown in Figure S2.1. We used linear regression models to analyse the relationship between LTL as the predictor and each protein as the outcome, adjusting for covariates collected at the corresponding visits for the LTL DNA samples, including age, sex, field center, LTL sample visit, BMI, eGFR and smoking status.

#### 2.8.2 Analysis of LTL PRS With Proteins Measured at ARIC Visit 2

To capture protein signatures for genetic predisposition of LTL, we conducted linear regression analyses between LTL PRS as the predictor and each of the 4955 plasma proteins as the outcome in White participants and Black participants separately. We analysed proteins measured at ARIC visit 2, which has a larger sample size than the visit 3 protein measurement. The analysis between LTL PRS and proteins measured at visit 3 was included in a sensitivity analysis.

Of the initial 15,792 participants, 12,219 had genetic data available for LTL PRS calculation. We excluded 2524 participants due to missing protein measurements or covariate data. For each individual protein, we further excluded participants whose protein levels were off 6 SD from the mean (minimum exclusion *N* = 14). The final sample for the LTL PRS proteomics analysis comprised 7587 White participants and 2094 Black participants. The schematic of the sample size determinations is shown in Figure S2.2. We adjusted for age at visit 2, sex, field center, eGFR at visit 2 and 10 principal components for genetic ancestry.

#### 2.8.3 Replication Analysis of LTL and LTL PRS With Proteins in CHS White Participants

In our proteomics analyses of both LTL and LTL PRS, we considered the analysis of White participants in ARIC as the discovery and that of ARIC Black participants and CHS White participants, respectively, as replications. The CHS replication analysis for LTL included White participants with either Southern blot LTL measurements (*N* = 1053) or WGS‐derived LTL estimates (*N* = 2431). In the WGS‐based LTL analysis, we conducted linear regression analyses adjusted for age, sex, eGFR, smoking status, BMI, WGS source, study field center, SomaLogic platform and study visit for DNA collection. In the Southern blot‐based LTL analysis, we conducted linear regression analyses adjusted for age, sex, eGFR, smoking status, BMI, study field center and SomaLogic platform.

In the LTL PRS analysis, we included 2333 White participants with both LTL PRS and proteomic data available, and adjusted for age, sex, eGFR, WGS source, study field center, SomaLogic platform, study visit for DNA collection and the first 10 principal components of genetic ancestry.

The statistical significance threshold for discovery in ARIC White participants was set at *p ≤* 1 × 10^−5^ after a Bonferroni correction for testing 4955 proteins. For each of the replication analyses, we applied a one‐sided Bonferroni correction based on the number of significant proteins identified in the discovery phase. This resulted in a significance threshold of *p* < 0.011 (0.1/9) for the LTL analysis and *p* < 0.0125 (0.1/8) for the LTL PRS analysis. All proteomics analyses were conducted using R (version 4.2.2).

#### 2.8.4 Comparison With Protein Measures by Different Platforms

For the top LTL‐associated proteins we examined the concordance between protein levels measured by different platforms. We calculated Spearman correlation coefficients for log2 scale measures by SomaScan 11k and Olink Explore HT platforms in plasma samples collected from 102 ARIC participants at visit 5. The Olink platform used Proximity Extension Assay (PEA) technology for protein quantification [38]. Details regarding sample characteristics, laboratory procedures and quality control measures for these assays have been described in detail elsewhere [39].

In addition, we measured GDF15 levels using an electrochemiluminescence immunoassay on a Cobas e 411 analyser (Elecsys, Roche Diagnostics). The assay has a detection limit of 400 pg/mL, measurement range of 400–20,000 pg/mL, and inter‐assay imprecision of 4.8%, 4.7% and 5.1% at GDF15 concentrations of 699, 1510 and 7264 pg/mL, respectively, in our ARIC samples.

#### 2.8.5 Bidirectional Two‐Sample Mendelian Randomization (MR) Analysis

MR analysis used genetic variants associated with an exposure as instrumental variables (IVs) to evaluate causal relationships between the exposure and outcome [40]. Among the 15 proteins that were significantly associated with either LTL or LTL PRS in the discovery phase, 12 proteins had *p*‐values < 0.05 in at least one of the replication cohorts (ARIC Black participants and CHS White participants) with consistent directions of association. For these 12 proteins, we conducted a bidirectional two‐sample MR analysis to investigate whether LTL is causally associated with these proteins, or vice versa. We used the same LTL GWAS summary statistics as those used in the LTL PRS calculation [19], and protein GWAS summary statistics from the population‐based deCODE Health study [41]. The latter reported a GWAS for 4907 plasma proteins measured by SomaScan in a cohort of 35,559 Icelanders.

In the forward analysis, that is, from LTL to each of these 12 proteins, we used IVs from the LTL GWAS by Codd et al. [19], which identified 197 sentinel SNPs within the 1 Mb window with a minor allele frequency (MAF) ≥ 0.1% that surpassed the GWAS threshold of *p* < 8.31 × 10^−9^. These 197 SNPs were further pruned using an LD r2 threshold of 0.01, resulting in 124 marked for inclusion in their final MR analyses. Of these 124 variants, 104 were also found in the deCODE protein GWAS [41]. After harmonisation and LD clumping, 89 variants were retained as IVs in the primary forward MR analysis (Table S10). To assess the potential influence of missing instruments, we conducted a sensitivity analysis by adding LD proxy variants for missing variants identified using a European‐ancestry LD reference panel (prioritising *r* ^2^ ≥ 0.8 when available; minimum *r* ^2^ ≥ 0.5). This reached a set of 101 instruments after harmonisation and LD clumping, and we conducted a sensitivity forward MR analysis using this IV set (MR results in Table S11; proxy variants listed in Table S13).

In the backward analysis, that is, from each protein to LTL, we used the summary statistics from the deCODE protein GWAS to select IVs for each protein. We applied the same filtering criteria as in the forward analysis. The clumping step was performed using the ClumpMR function in the TwoSampleMR R package [42], which uses the European genome in the 1000 Genomes Project as a reference panel.

The primary MR method we employed was the inverse‐variance weighted (IVW) method. This approach used a weighted linear regression, where the associations between IVs and the outcome were regressed on the IV‐exposure associations. The weighting was determined by the inverse of the variance of each IV‐outcome association [20]. We also performed sensitivity analyses, including weighted median [43], mode‐based estimate (MBE) [44], and MR‐Egger methods [40], that are robust to the violation of various MR assumptions. To assess that the assumption of no‐horizontal pleiotropy was met, we used the MR‐Egger intercept test to detect pleiotropy [40]. Additionally, we used the Mendelian Randomization Pleiotropy Residual Sum and Outlier (MR‐PRESSO) method to evaluate and adjust for potential horizontal pleiotropy effects through outlier removal [45]. All analyses were performed using the MendelianRandomization (version 0.7.0) [46] and TwoSampleMR (version 0.5.6) [42] packages in R (version 4.2.2). Based on the tests we conducted (IVW, weighted median, MBE, MR‐Egger and MR‐PRESSO methods), we considered MR evidence to be robust using the following criteria: a *p*‐value in at least one MR approach that is less than the Bonferroni‐corrected threshold (*p* < 0.05/12 proteins = 0.004) and a *p*‐value < 0.05 in at least one other approach.

#### 2.8.6 Association Analysis Between LTL and AAA

The prospective association between LTL and AAA was assessed using a Cox proportional hazards regression. From the initial cohort of 15,792 ARIC participants, we excluded individuals missing LTL measurements (*N* = 2764) or covariates (*N* = 878), as well as those who were diagnosed with AAA prior to the sample visit for LTL measurements (*N* = 51). Participants without AAA were censored at whichever of the following occurred first: loss to follow‐up, the end date of the study administrative censoring on December 31, 2019 (2017 for Jackson participants), or at the time of death. The final analysis included 12,099 participants, with 609 incident clinical AAA cases over a median follow‐up of 24.9 years. The schematic of the sample size determinations is shown in Figure S2.3. In the primary analysis, we analysed LTL as a continuous variable and adjusted for the following potential confounders based on our knowledge about their associations with both AAA and LTL: age, sex, race, field center, BMI, prevalent diabetes and hypertension, smoking status, eGFR and the corresponding LTL sample visit. The covariates were all measured at the visits corresponding to the WGS samples for LTL estimation. We additionally analysed LTL in quartiles and conducted a trend test in which rank‐ordered LTL quartiles were analysed as a numerical variable (i.e., 1–4). All the analysis used the survival package [47] in R (version 4.2.2).

#### 2.8.7 Causal Mediation Analysis

For the six proteins that emerged from the forward MR analysis linking LTL to protein levels (KDR, TNFRSF17, GDF15, ST3GAL6, CHL1 and GP1Bα), we explored the direct associations between each protein and AAA incidence. Adjustments were made for covariates measured at the visit for protein samples (visit 3), including age, sex, race, field center, BMI, prevalent diabetes or hypertension, smoking status and eGFR.

GDF15 was statistically significantly associated with AAA from the above direct association analysis. We then assessed whether GDF15 mediated the pathway from LTL to AAA. To reduce reverse causation, we analysed only LTL measures from visit 1, 2 or 3 samples and protein mediator data that was assessed at visit 3.

The outcome was incident AAA diagnosed after visit 3. From the initial cohort of 15,792 ARIC participants, we excluded participants without LTL measurements (*N* = 2764), participants with LTL measurements not from visits 1, 2 or 3 (*N* = 216), participants missing for visit 3 protein measurements or covariates (*N* = 3089) and participants who had AAA diagnosed before visit 3 (*N* = 91). The final sample included 9632 individuals with 495 incident clinical AAAs ascertained over a median follow‐up of 24.5 years. Covariate adjustment in the mediation analysis included corresponding LTL sample visit, age, sex, race, field center, eGFR, BMI, prevalent diabetes and hypertension and smoking status.

A counterfactual framework for causal mediation was used in our analysis [48], which involves a clear causal structure and enables the simultaneous estimation of causal estimates [49]. This approach was performed under a regression‐based approach to estimate direct effects (LTL and AAA association, not mediated by GDF15), indirect effects (mediation due to GDF15) and total effects (direct and indirect effects) [49]. An interaction term was included to allow for effect estimation in the presence of exposure‐mediator interaction. Counterfactual effect estimates and 95% confidence intervals (CIs) were calculated through the delta method [50]. The proportion of association mediated by protein was calculated as Direct Effect × (Indirect Effect − 1)/(Total Effect − 1) [49]. All the mediation analyses were conducted using the CMAverse package [50] in R (version 4.2.2).

## 3 Results

Table S3 shows the demographic and clinical characteristics of the samples included in the LTL proteomics and LTL AAA analyses. The sample characteristics for the LTL PRS proteomics analysis are shown in Table S4.

### 3.1 Associations of LTL With Proteins

As shown in Table 1, the discovery analysis in ARIC White participants identified nine proteins significantly associated with LTL after Bonferroni correction (*p* ≤ 1 × 10^−5^). These nine proteins were then evaluated for replication in ARIC Black and CHS White participants, using a one‐sided Bonferroni‐corrected significance threshold of *p* < 0.011 (0.1/9). Three proteins (MZB1, PLOD3 and COL28A1) met this threshold for replication and exhibited consistent directions of association in ARIC Black participants. In CHS White participants, three proteins (TNFRSF17, MZB1 and CHL1) were successfully replicated for the WGS‐derived LTL measurements, and four proteins (GDF15, THPO, PLOD3 and CHL1) replicated for the Southern blot‐based LTL measurements. All replicated proteins maintained consistent directions of association across cohorts.

**TABLE 1:** Proteins significantly associated with LTL in the discovery analysis of ARIC White Participants.

Aptamer ID	Protein	Gene symbol	ARIC White Participants (*N* = 8055)		ARIC Black Participants (*N* = 1668)		CHS White Participants (WGS) (*N* = 2431)		CHS White Participants (southern blot) (*N* = 1053)	
			Estimate (95% CI) §	*p*	Estimate (95% CI) §	*p*	Estimate (95% CI) §	*p*	Estimate (95% CI) §	*p*
4374_45	Growth/Differentiation factor 15‡	GDF15‡	−0.072 (−0.092, −0.053)	7.97E‐13	−0.049 (−0.095, −0.002)	0.039	−0.049 (−0.095, −0.002)	0.040	**−0.085 (−0.128, −0.042)**	**0.0001**
4990_87	Platelet glycoprotein Ib alpha chain	GP1Bα	0.071 (0.050, 0.091)	1.55E‐11	0.056 (0.008, 0.1049)	0.020	0.03 (−0.01, 0.07)	0.098	−0.003 (−0.038, 0.032)	0.869
11212_7	Thioredoxin domain‐containing protein 5	TXNDC5	−0.053 (−0.072, −0.034)	7.84E‐08	−0.043 (−0.088, 0.002)	0.063	−0.04 (−0.08, 0.01)	0.103	−0.038 (−0.075, −0.001)	0.047
5947_90	Thrombopoietin‡	THPO‡	−0.055 (−0.076, −0.035)	9.05E‐08	−0.035 (−0.07, 0.005)	0.084	−0.05 (−0.09, −0.01)	0.026	**−0.066 (−0.10, −0.030)**	**0.0003**
2665_26	Tumour necrosis factor receptor superfamily member 17‡	TNFRSF17‡	−0.054 (−0.075, −0.034)	2.56E‐07	−0.002 (−0.047, 0.044)	0.942	**−0.05 (−0.09, −0.01)**	**0.009**	−0.001 (−0.037, 0.035)	0.954
16322_10	Marginal zone B‐ and B1‐cell‐specific protein*‡	MZB1*‡	−0.056 (−0.078, −0.034)	4.88E‐07	**−0.070 (−0.118, −0.023)**	**0.003**	**−0.07 (−0.11, −0.03)**	**0.001**	−0.039 (−0.077, −0.002)	0.040
10612_18	Procollagen‐lysine,2‐oxoglutarate 5‐dioxygenase 3*‡	PLOD3*‡	−0.056 (−0.078, −0.034)	4.90E‐07	**−0.066 (−0.114, −0.018)**	**0.006**	−0.05 (−0.09, −0.01)	0.020	**−0.054 (−0.089, −0.018)**	**0.0035**
8958_51	Neural cell adhesion molecule L1‐like protein‡	CHL1‡	−0.054 (−0.076, −0.032)	1.50E‐06	−0.046 (−0.091, 0.0007)	0.053	**−0.07 (−0.11, −0.03)**	**0.001**	**−0.063 (−0.099, −0.027)**	**0.0007**
10702_1	Collagen alpha‐1 (XXVIII) chain*	COL28A1*	−0.043 (−0.060, −0.025)	2.97E‐06	**−0.060 (−0.103, −0.018)**	**0.005**	0.06 (0.01, 0.11)	0.030	0.011 (−0.036, 0.059)	0.638

*Note:* Bold font: proteins that replicated at *p* < 0.011 (0.1/9) in ARIC black participants and CHS white participants based on one‐sided Bonferroni correction adjusted for the number of significant proteins identified in the discovery analysis. Protein replicated in ARIC black participants (*) and CHS white participants (‡) at *p* < 0.011 (0.1/9). §Effect size and 95% confidence interval (CI) for the association between LTL and each protein in the respective cohorts: ARIC and CHS (WGS)—increment in SD unit of log2 protein per 1 unit increase in inversely normalised LTL; CHS (Southern blot): increment in SD unit of log2 protein per 1 kilobase pair increase in LTL.

In a sensitivity analysis in ARIC White participants that additionally adjusted for leisure time physical activity measured as metabolic equivalent task (MET) minutes/week [51], prevalent coronary heart disease and diabetes, and alcohol consumption, the LTL protein associations remained highly consistent with those of the primary analysis, showing similar effect estimates and maintaining statistical significance for all nine proteins (Table S5).

### 3.2 Associations of LTL PRS With Proteins

As shown in Table 2, in the discovery analysis in ARIC White participants, eight proteins exceeded the Bonferroni corrected significance threshold (*p* ≤ 10^−5^). In CHS White participants, three proteins (THPO, GP1Bα and PEAR1) were replicated based on the one‐sided Bonferroni correction (*p* < 0.1/8 = 0.0125). Although no proteins were significantly replicated in ARIC Black participants, KDR showed a nominally significant association (*p* = 0.02) with the same direction of association as the discovery result.

**TABLE 2:** Proteins significantly associated with LTL PRS in the discovery analysis of ARIC White Participants.

Aptamer ID	Protein	Gene symbol	ARIC White Participants (*N* = 7587)		ARIC Black Participants (*N* = 2094)		CHS White Participants (*N* = 2333)	
			Estimate (95% CI) §	*p*	Estimate (95% CI) §	*p*	Estimate (95% CI) §	*p*
5947_90	Thrombopoietin‡	THPO‡	−0.051 (−0.071, −0.032)	2.33E‐07	−0.026 (−0.068, 0.016)	0.225	**−0.018 (−0.027, −0.009)**	**1.74E‐04**
4990_87	Platelet glycoprotein Ib alpha chain‡	GP1Bα‡	0.054 (0.033, 0.075)	5.67E‐07	0.007 (−0.040, 0.054)	0.778	**0.015 (0.006, 0.023)**	**0.001**
8275_31	Platelet endothelial aggregation receptor 1‡	PEAR1‡	0.054 (0.032, 0.075)	7.67E‐07	0.003 (−0.042, 0.048)	0.902	**0.014 (0.006, 0.022)**	**0.001**
6947_4	Type 2 lactosamine alpha‐2,3‐sialyltransferase	ST3GAL6	0.046 (0.027, 0.065)	1.60E‐06	−0.015 (−0.057, 0.026)	0.471	0.010 (0.001, 0.018)	0.021
3651_50	Vascular endothelial growth factor receptor 2	KDR	0.051 (0.03, 0.073)	3.00E‐06	0.056 (0.009, 0.102)	0.019	0.005 (−0.004, 0.014)	0.276
11128_29	Transmembrane protein 132C	TMEM132C	0.040 (0.023, 0.058)	4.03E‐06	−0.022 (−0.058, 0.014)	0.228	0.005 (−0.003, 0.014)	0.200
7185_29	Platelet glycoprotein V	GP5	0.050 (0.029, 0.071)	4.32E‐06	0.033 (−0.018, 0.083)	0.206	0.005 (−0.006, 0.017)	0.367
11178_21	Sushi, von Willebrand factor type A, EGF and pentraxin domain‐containing protein 1	SVEP1	−0.049 (−0.070, −0.028)	4.65E‐06	0.0003 (−0.045, 0.046)	0.990	0.001 (−0.008, 0.009)	0.875

*Note:* Bold font: proteins that replicated at *p* < 0.0125 (0.1/8) in CHS white participants based on one‐sided Bonferroni correction adjusted for the number of significant proteins identified in the discovery set; no proteins replicated in ARIC black participants at this statistical threshold. Protein replicated in CHS white participants‡ at *p* < 0.0125 (0.1/8). §The effect size and 95% confidence interval (CI) for the association between LTL PRS and each protein in the respective cohorts: increment in SD unit of log2 protein per 1 unit increase in LTL PRS.

The sensitivity analysis between LTL PRS and proteins measured at visit 3 was shown in Tables S6 and S7. Additionally, in a sensitivity analysis adding nine proxy SNPs into the LTL PRS calculation, six of the eight proteins identified in the primary analysis retained their significance and the same direction of estimates (THPO, KDR, SVEP1, GP1Bα, PEAR1 and ST3GAL6) in ARIC White participants. These proteins showed similar replication in ARIC Black participants to that observed in the primary analysis. Detailed results on the associations from the sensitivity analyses by race are provided in Tables S8 and S9.

### 3.3 MR Analysis Results

In our discovery analysis in ARIC White participants, 15 unique proteins demonstrated statistically significant associations with LTL or LTL PRS (Tables 1 and 2). Among these 15 proteins, 12 had *p*‐values < 0.05 in at least one of the replication cohorts (ARIC Black participants and CHS White participants) with consistent association directions and thus moved forward to the MR analysis.

**FIGURE 1:**
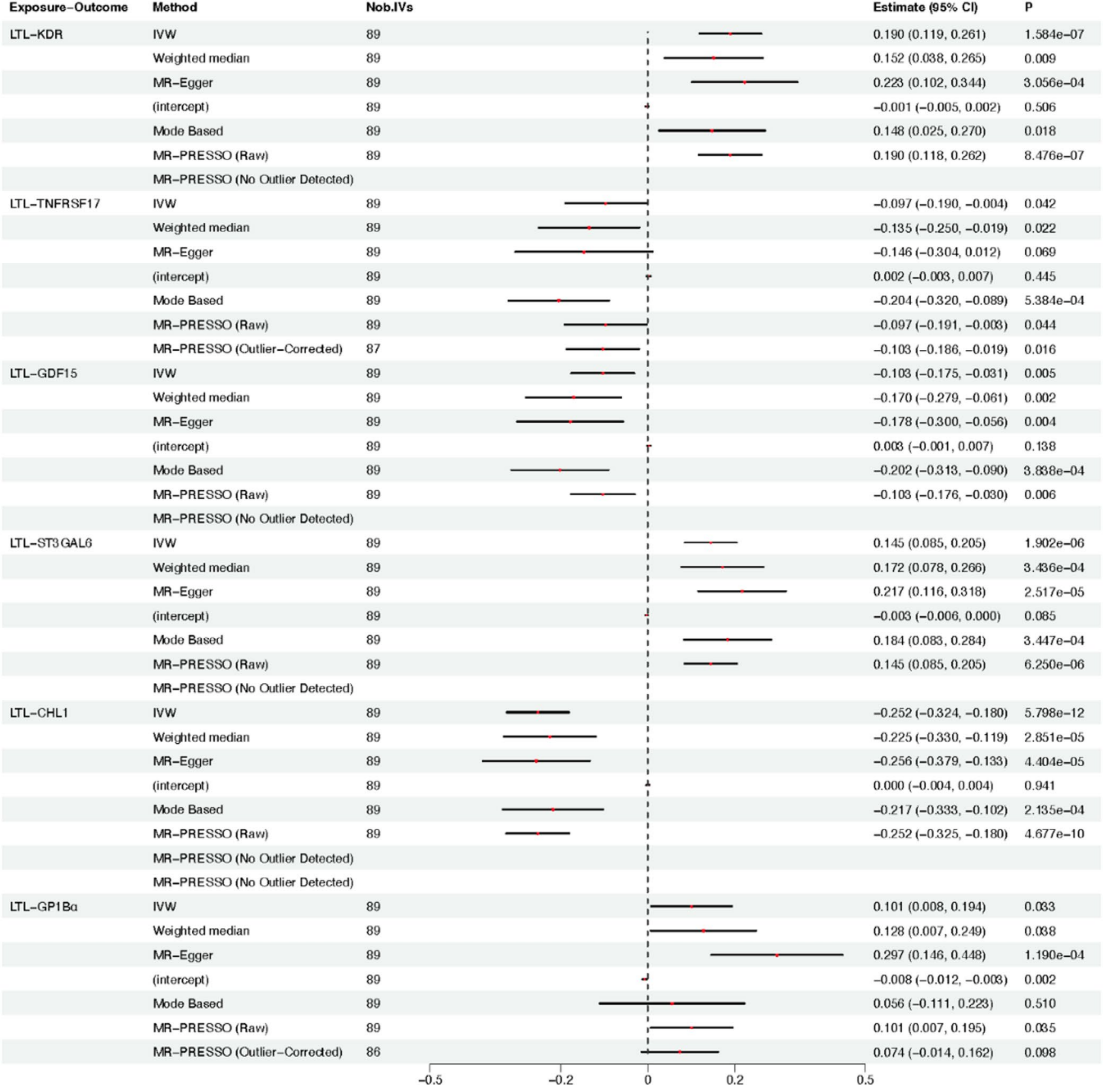
Forward Mendelian randomization analysis showing proteins with a *p*‐value < 0.004 in at least one method (Bonferroni‐corrected for 12 tests) and *p* < 0.05 in at least one additional method. Estimates represent the causal effect of LTL on protein levels: Increment in SD unit of rank‐inverse normal transformed protein per 1 unit increase in genetically instrumented LTL. IVW, inverse‐variance weighted method; MR, mendelian randomization; Estimate (95% CI), estimated effect and 95% confidence interval for LTL‐protein association; Nob. IVs, number of instrumental variables.

In the forward MR analysis from LTL to each of the 12 proteins, the F‐statistics for the instrumental variables were all greater than 10, indicating strong instrumental variables. As shown in Figure 1, LTL showed statistically significant associations with six proteins based on our criteria (a *p*‐value in at least one MR approach that is less than the Bonferroni‐corrected threshold (*p* < 0.05/12 proteins = 0.004) and a *p*‐value < 0.05 in at least one other approach). However, the MR‐Egger intercept test indicated possible pleiotropy influence for GP1Bα. The MR‐PRESSO test after outlier correction was not significant for GP1Bα, indicating less robust results. All these LTL‐protein pairs show the same directions of associations in the MR analysis as in the LTL proteomics analysis. Sensitivity analyses incorporating additional IVs identified through LD proxies for unavailable LTL instruments yielded consistent results, supporting the robustness of the primary forward MR findings (Table S11).

**FIGURE 2:**
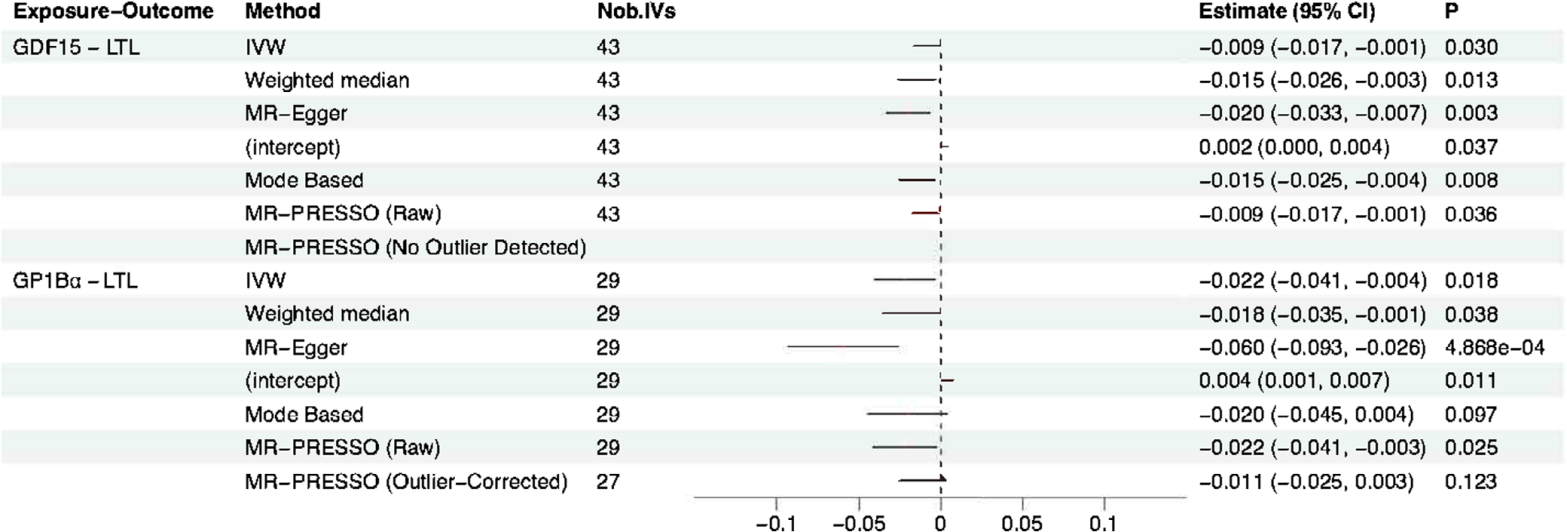
Backward Mendelian randomization analysis showing proteins with a *p*‐value < 0.004 in at least one method (Bonferroni‐corrected for 12 tests) and *p* < 0.05 in at least one additional method. Estimates represent the causal effect of protein levels on LTL: increment in LTL per 1 unit increase in genetically instrumented protein.

In the backward analysis from each of the 12 proteins to LTL, all F‐statistics for IVs were above the threshold of 10, and GP1Bα and GDF15 showed statistically significant associations with LTL (Figure 2). However, the pleiotropic effects detected in the MR‐Egger intercept tests for GDF15 and the non‐significant result after outlier correction for GP1Bα indicate that the MR evidence for the causation for the backward direction is not robust. Detailed information on the MR analysis results and a summary of the IVs is provided in Tables S10–S15.

### 3.4 Prospective Association Between LTL and AAA

**FIGURE 3:**
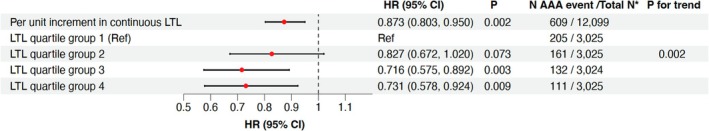
Prospective association between LTL and incident AAA in ARIC (1987–2019) showing hazard ratio of AAA associated with continuous LTL or LTL quartile group. Covariate adjustment included age, sex, race, field center, BMI, prevalent diabetes and hypertension, smoking status, eGFR and corresponding LTL sample visit. * Number of AAA events/Total number of participants. AAA, abdominal aortic aneurysm; CI, confidence interval; HR, hazard ratio.

The prospective Cox regression analysis revealed a statistically significant association between longer LTL at baseline and decreased risk of AAA (HR per unit increase of LTL = 0.873, 95% CI [0.803, 0.950]) (Figure 3). The proportional hazard assumption using the Schoenfeld residual test was not violated (*p* = 0.18). Consistent results were observed when analysing LTL by quartile groups, with a significant linear trend between LTL and AAA risk (*p* = 0.002) (Figure 3).

Since white blood cell count may confound the association between LTL and AAA, we conducted a sensitivity analysis by additional adjustment for white blood cell count measured at visits 1 or 2. This analysis included a total of 10,161 participants (499 AAA events). The results are consistent with the primary analysis, with a longer LTL still showing a statistically significant association with decreased AAA risk (HR = 0.891, 95% CI [0.811, 0.979]), *p* = 0.02 for linear trend (Table S16).

### 3.5 Mediation Analysis of LTL, Proteins and AAA

Of the six proteins that emerged from the forward MR analysis from LTL to the proteins (KDR, TNFRSF17, GDF15, ST3GAL6, CHL1 and GP1Bα), GDF15, an emerging prognostic biomarker of aging and cellular stress [61], was significantly associated with AAA incidence (HR = 1.129, 95% CI [1.029, 1.238]) after correcting for multiple testing (*p* < 0.05/6). Detailed associations for these proteins can be found in Table S17. In the mediation analysis for LTL‐GDF15‐AAA, GDF15 showed a statistically significant proportion of the mediation effect on the pathway from LTL to AAA (12.4%, *p* = 0.028; Table S18). These findings suggest that GDF15 levels are involved in the vascular pathways downstream of LTL that are relevant to the development of AAA.

### 3.6 Comparison With Protein Measures by Different Platforms

Of the 15 proteins that were significantly associated with either LTL or LTL PRS in the discovery analysis, 11 were available on the Olink Explore HT panel and one protein (GDF15) was measured using the Roche assay in plasma samples of 102 ARIC participants. Eight of the proteins showed strong correlations (Spearman *r* > 0.6) between platforms, with GDF15 showing the strongest correlation (*r* = 0.94) (Table S19). THPO and PEAR1 showed moderate correlations (*r* = 0.44 and 0.58, respectively). PLOD3 and COL28A1 showed weak correlations (*r* < 0.4) (Table S19).

## 4 Discussion

This study, conducted in the large community‐based ARIC cohort, identified 15 proteins that were associated with LTL or LTL PRS in ARIC Whites. Of the nine proteins identified in our LTL proteomics analysis, three (MZB1, PLOD3 and COL28A1) were replicated in ARIC Blacks, three (TNFRSF17, MZB1 and CHL1) in CHS Whites for WGS‐based LTL measures, and four (GDF15, THPO, PLOD3 and CHL1) in CHS Whites for Southern blot‐based LTL. All of these 15 proteins except GDF15 represent novel findings, as no previous human studies have reported associations between circulating levels of the 14 proteins and leukocyte telomere length. Furthermore, the forward MR analysis suggested that LTL has a robust causal influence on five of the proteins (KDR, TNFRSF17, GDF15, ST3GAL6 and CHL1). We also found that GDF15 mediated 12.4% of the effect in the prospective association between LTL and future risk of AAA.

Previous research has widely recognised shorter LTL as a hallmark of aging [3, 19, 54] and associated shorter LTL with numerous age‐related diseases [4, 5, 6] and certain cancers [7, 8, 9, 19]. However, the underlying biological mechanisms mediating the relationship between shorter telomere length and age‐related diseases have not been fully elucidated. Telomere attrition and maintenance are influenced by genetic factors and other biological mechanisms such as cellular replication, oxidative stress, glucocorticoid exposure, mitochondrial dysfunction, and inflammation, collectively impacting aging and disease susceptibility [54, 54]. Our research integrates large‐scale proteomics, Mendelian randomization, and prospective cohort analyses with a mediation component. This approach not only improves the understanding of the biological processes involved in aging but also elucidates possible biological mechanisms mediating the relationship between LTL and AAA, which offers new perspectives on how telomere dynamics might influence aging and age‐related conditions.

Among the identified proteins, our study found that GDF15 mediated a significant portion (12.4%) of the pathway from LTL to AAA, providing new insights into the molecular mechanisms of age‐related vascular disease. Accumulating evidence indicates that GDF15 levels serve as an independent prognostic marker for various age‐associated diseases, including dementia, ischemic heart disease, heart failure, atherosclerosis, and hypertrophic cardiomyopathy [55, 56, 57]. A previous Mendelian randomization analysis also suggested a causal relationship in which longer telomeres were associated with lower levels of GDF15 [58], which is consistent with the findings in our study.

The biological mechanisms underlying the LTL–GDF15–AAA relationship likely involve multiple pathways in which GDF15 plays a fundamental role. Shortened telomeres can trigger increased cellular senescence, leading to an inflammatory environment that promotes GDF15 secretion [59]. Elevated GDF15 may then influence immunomodulation, cytokine regulation, and extracellular matrix homeostasis, which are important for maintaining aortic wall integrity in AAA pathophysiology [59, 60]. Overall, these findings broaden the link between telomere biology, aging and aging‐related disease and highlight GDF15 as a candidate mediator that warrants further validation and mechanistic investigation for AAA.

Our research also suggests a potential causal relationship between telomere length and ST3GAL6 expression. While no direct mechanistic link has been established, longer telomere length and ST3GAL6 expression have been implicated in cancer development [8, 61, 62]. ST3GAL6, an enzyme responsible for glycoprotein and glycolipid sialylation, shows increased expression in several cancers, including colorectal cancer, lung adenocarcinoma, and hepatocellular carcinoma [61, 62, 63]. Longer telomere length has been associated with elevated risks of lung, ovarian, bladder and melanoma cancers [8]. Mechanistically, longer telomeres may promote genomic instability and enhance cellular proliferation [9]. This mechanism could potentially influence ST3GAL6 expression through altered signalling pathways associated with increased proliferation [62]. ST3GAL6 overexpression may also affect cell adhesion and migration, as observed in multiple myeloma [62]. Further investigation is needed to elucidate the potential interactions between telomere biology and ST3GAL6 expression in cancer development and progression as well as in other conditions.

Our study identified an inverse causal relationship between LTL and CHL1. CHL1 is a member of the L1 family of neural cell adhesion molecules, which play a crucial role in nervous system development, synaptic plasticity [64], and the development of cancers [65, 66]. A large‐scale epigenome‐wide association study reported that telomere length was associated with differential DNA methylation at a CpG site (cg07414525) within the CHL1 gene that showed a strong negative correlation with LTL across populations of European and African ancestry [67]. Given that both telomeres and CHL1 are involved in maintaining chromosomal stability, the observed causal relationship between LTL and CHL1 may reflect a shared biological pathway. Further mechanistic studies are needed to elucidate the specific molecular interactions between telomere attrition and CHL1 expression.

KDR, also known as Vascular Endothelial Growth Factor Receptor 2 (VEGFR2), plays a critical role in angiogenesis, vascular development and the maintenance of endothelial cell functions [68]. The association between LTL and KDR could be linked through the pathways of endothelial cell and vascular function. Specifically, longer telomeres, which are indicative of greater cellular replicative potential and reduced cellular aging [69], may enhance the ability of endothelial cells to maintain robust KDR signalling. Given its central role in endothelial biology and angiogenesis, KDR represents a biologically plausible pathway for vascular remodelling and a candidate pathway that may contribute to AAA risk.

TNFRSF17 is crucial in B‐cell survival and promotes cell survival by activating NF‐κB signalling [70]. Since TNFRSF17 activates NF‐κB signalling, decreased levels of TNFRSF17 would likely result in reduced NF‐κB activation and consequently lower inflammatory signalling [70]. Our MR results indicate that a longer LTL is causally associated with decreased levels of TNFRSF17. While the precise mechanisms remain unclear, it is plausible that a longer LTL may enhance cellular robustness and stability, potentially leading to a reduced need for the survival signals typically provided by TNFRSF17. This decrease could result from an overall reduction in cellular stress, as longer telomeres may help protect cells from replicative senescence and the stress responses that usually require TNFRSF17 activity.

Previous MR analyses have supported a causal association between telomere length and AAA [8, 15], which is consistent with our finding. However, most available population‐based data were derived from case–control studies [5]. To the best of our knowledge, our study is the largest population‐based biracial cohort to prospectively investigate the association between telomere length and AAA to date. Consistent with previous findings, our results suggest that shorter telomeres may promote accelerated cellular aging, potentially contributing to an increased risk of AAA. Furthermore, our results highlight GDF15, an aging biomarker, as a potential mediator in the pathway from LTL to AAA. Given the modest evidence for mediation, this finding should be interpreted cautiously and requires further validation.

Strengths of this study include the agnostic and large‐scale nature of our proteomic analyses, independent replications in a different cohort using LTL measurements obtained with a different platform, the prospective study design with a long follow‐up, and the causal inferences that Mendelian randomization experiments allow.

Several limitations must also be acknowledged. First, this proteomics platform does not fully capture the human proteome. As an aptamer‐based platform, SomaScan may not always accurately capture the intended protein targets, since cross‐reactivity and non‐specific binding can affect measurement accuracy [71]. However, a majority of the proteins we identified showed strong correlations across platforms (Table S19), supporting the reproducibility of SomaScan measurements for those proteins. Second, the generalizability of the study results may be limited to White and Black mid‐life adults in the United States as the genetic predispositions and environmental interactions influencing telomere length might differ significantly across ethnic backgrounds. Third, the GWAS for the MR analysis and derivation for the LTL PRS predominantly included individuals of European ancestry, which limits the applicability of our findings to other populations. This could explain the weaker replication results for the LTL PRS proteomics associations observed in the ARIC Black participants. Fourth, while our results identify a significant mediating effect by GDF15 from LTL to AAA risk, the detailed biological pathways still need to be elucidated through experimental studies.

In conclusion, this study identified novel proteomic signals related to telomere length and explored potential biological mechanisms linking telomere length to AAA. Our mediation analysis suggests that GDF15 may act as a candidate mediator or biomarker in the LTL–AAA pathway, warranting further validation. A comprehensive understanding of these connections could provide insights into the biological mechanisms of telomere attrition, aging and age‐related diseases. Additional research into the identified proteins and their potential roles in AAA development is warranted.

## Author Contributions

Aixin Li conducted the statistical analyses and drafted the manuscript. Nathan Pankratz and John A. Lane derived the LTL estimates based on WGS data in ARIC and CHS. Thomas R. Austin analysed the CHS data. Weihong Tang designed the study and provided funding. Weihong Tang and Weihua Guan supervised the data analyses, manuscript writing and manuscript revision. All authors were involved in data interpretation and critical revision of the manuscript. All authors read and approved the final version of the manuscript.

## Funding

This work was supported by the National Institute on Aging, R21AG072530, and the National Heart, Lung, and Blood Institute, R01HL155209.

## Disclosure

Data Sharing Statement: The parent studies ARIC and CHS deposit individual‐level data to BioLINCC (https://biolincc.nhlbi.nih.gov/home/) and dbGaP (https://www.ncbi.nlm.nih.gov/gap/) periodically, where these data are available upon approval by those repositories. The LTL GWAS summary statistics used in this study were obtained from Codd et al. (Nat Genet. 2021;53 (10): 1425–1433). The protein GWAS summary statistics from the deCODE study were obtained from Ferkingstad et al. (Nat Genet. 2021; 53 (12): 1712–1721). Upon reasonable request, the corresponding authors (Drs. Tang and Guan) will make the analytical methods, analytical code and summary statistics generated by this project available to other researchers.

## Conflicts of Interest

The authors declare no conflicts of interest.

## Supporting information


**Appendix S1:** jcmm71047‐sup‐0001‐AppendixS1.docx.

## Data Availability

The data that support the findings of this study are available on request from the corresponding author.
