# Supplementary material for: Plasma Proteome Signature for Leukocyte Telomere Length and Its Link to Abdominal Aortic Aneurysm
Source: J Cell Mol Med. 2026 Feb 12;30(3):e71047. doi: 10.1111/jcmm.71047 (PMC12895465; doi:10.1111/jcmm.71047)
Supplement: Supplementary file 1 — Appendix S1: jcmm71047‐sup‐0001‐AppendixS1.docx. [file JCMM-30-e71047-s001.docx]

**Supplementary Tables and Figures to:**

**Plasma proteome signature for leukocyte telomere length and its link to abdominal aortic aneurysm**

Aixin Li MPH^1^, Thomas R. Austin, PhD^2^, Brian T. Steffen PhD^1,3^, Ingrid Jacobson MPH^1^, Jiaqi Xie MS^4^, Nathan Pankratz PhD^5^, John A. Lane PhD^5^, Annette Fitzpatrick, PhD^6^, Joshua C. Bis, PhD^2^, Dan E. Arking PhD^4^, Thomas Mosley PhD^7^, Sanaz Sedaghat PhD^1^, James S. Pankow PhD^1^, Pamela L. Lutsey PhD^1^, Weihua Guan PhD*^8^, and Weihong Tang MD, PhD*^1^

^1^ Division of Epidemiology and Community Health, School of Public Health, University of Minnesota, Minneapolis, MN, USA.

^2^ Cardiovascular Health Research Unit, Department of Medicine, University of Washington, Seattle WA USA

^3^ Division of Computational Health Sciences, Department of Surgery, University of Minnesota Medical School, Minneapolis, MN, USA

^4^ McKusick-Nathans Institute, Department of Genetic Medicine, Johns Hopkins University School of Medicine, Baltimore, MD, USA.

^5^ Division of Molecular Pathology and Genomics, University of Minnesota, Minneapolis, MN, USA.

^6^ Departments of Family Medicine and Epidemiology, Schools of Medicine and Public Health, University of Washington, Seattle, WA, USA.

^7^ Department of Medicine, Memory Impairment and Neurodegenerative Dementia (MIND) Center, University of Mississippi, Jackson, Mississippi, USA.

^8^ Division of Biostatistics and Health Data Science, School of Public Health, University of Minnesota, Minneapolis, MN, USA

* Equally-contributing last/senior authors

**Table of Contents**

Figure S1: General Analysis Workflow

Table S1: SNPs Used in the Primary PRS Calculation

Table S2: Information on the Proxy SNPs Included in the Sensitivity PRS Calculation

Figure S2.1-2.3: Participants Exclusion Scheme

Table S3: Baseline Characteristics of Participants in the LTL-Proteomics and LTL-AAA Analyses

Table S4: Baseline Characteristics of Participants Included in the LTL PRS Proteomics Analysis in ARIC and CHS

Table S5: Significant Proteins in the Primary LTL Proteomics Analysis and Sensitivity Analysis in ARIC White Participants

Table S6: Visit 3 Replication of Eight LTL PRS-Protein Associations Identified at Visit 2 in ARIC White Participants

Table S7: Visit 3 Replication of Eight LTL PRS-Protein Associations in ARIC Black Participants Identified at Visit 2 in ARIC White Participants

Table S8: Sensitivity Analysis of Eight LTL PRS–Protein Associations in ARIC White Participants at Visit 2 After Adding Nine Additional Proxy SNPs to the PRS

Table S9: Sensitivity Analysis of Eight LTL PRS–Protein Associations in ARIC Black Participants at Visit 2 After Adding Nine Additional Proxy SNPs to the PRS

Table S10: Forward Direction Mendelian Randomization (MR) Analysis Results

Table S11: Forward Direction Mendelian Randomization (MR) Sensitivity Analysis Results with Added LD Proxies

Table S12: Summary of Instrumental Variables Used in the LTL-Protein MR Analysis

Table S13: LD Proxy Variants Used in the Forward MR Sensitivity Analysis

Table S14: Backward Direction Mendelian Randomization (MR) Analysis Results

Table S15: Summary of Instrumental Variables Used in the Protein-LTL MR Analysis

Table S16: Sensitivity Analysis of LTL with Incident AAA by Additional Adjustment for White Blood Cell Count in ARIC (1987-2019)

Table S17: Associations Between the LTL-Associated Proteins and AAA Incidence in ARIC (1987-2019)

Table S18: Mediation Analysis of the Association Between LTL and AAA through Proteins as Mediators in ARIC

Table S19: Spearman Correlation for Protein Measures by SomaScan vs Other Assays in ARIC for Selected Top LTL Proteins

| 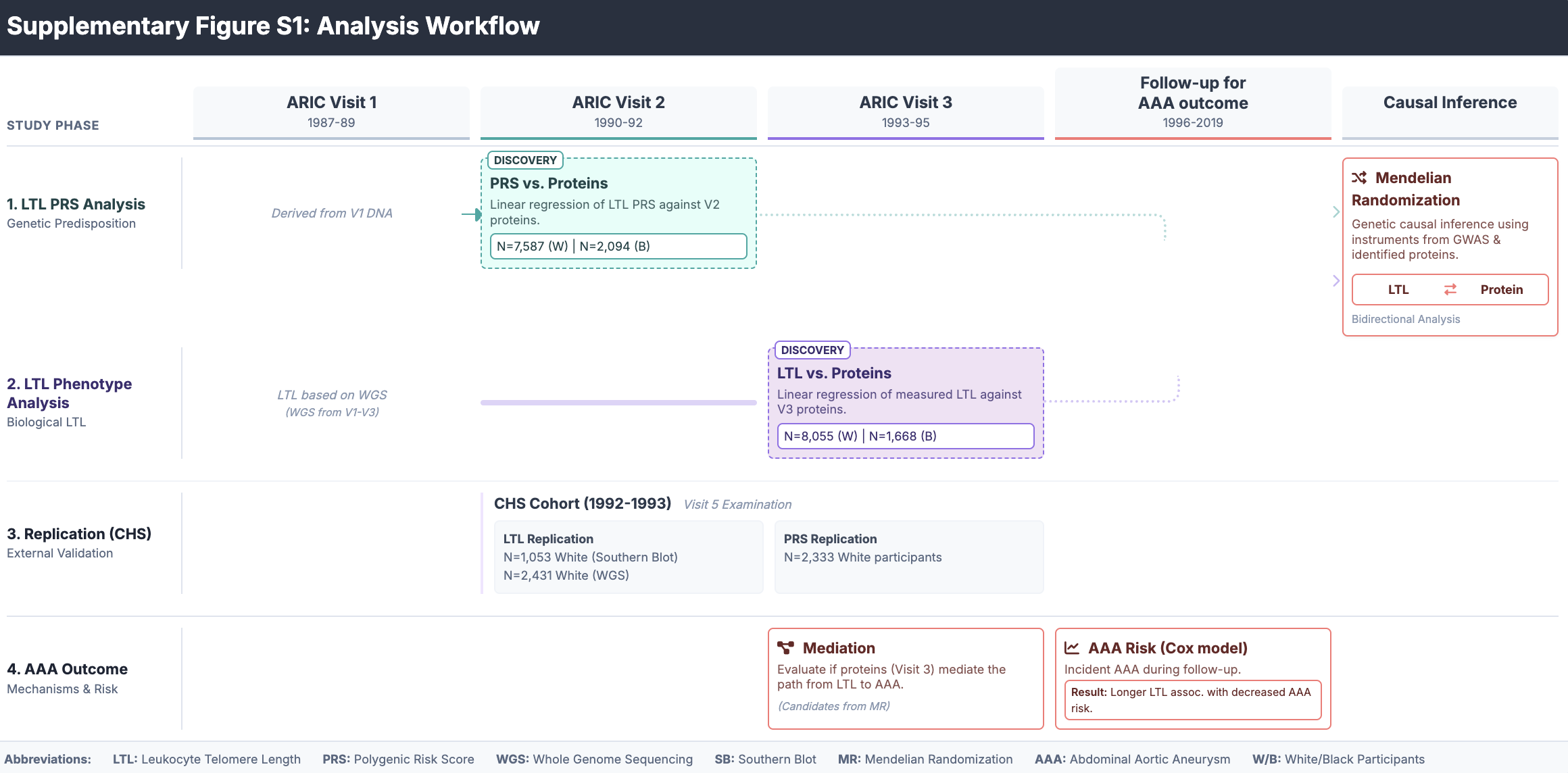  **Table S1. SNPs Used in the Primary PRS Calculation** | | | | | | | | | | | |
| --- | --- | --- | --- | --- | --- | --- | --- | --- | --- | --- | --- |
| **SNP** | **GENE** | **Chr** | **BP_GRCh38** | **BP_GRCh37** | | **Effect Allele(A1)** | | **Other Allele** | **FREQ(A1)** | **MAF** | **Beta (95% CI)*** |
| rs187540244 | EXOSC10 | 1 | 11164270 | | 11224327 | | G | A | 0.995 | 0.005 | 0.093 (0.064 , 0.121) |
| rs66731853 | CDA | 1 | 20589745 | | 20916238 | | G | A | 0.683 | 0.317 | 0.018 (0.013 , 0.022) |
| rs17185038 | RPA2 | 1 | 27893147 | | 28219658 | | C | G | 0.935 | 0.065 | 0.025 (0.017 , 0.033) |
| rs6669563 | SPOCD1 | 1 | 31814028 | | 32279629 | | G | A | 0.562 | 0.438 | -0.018 (-0.022 , -0.015) |
| rs3768321 | PABPC4 | 1 | 39570256 | | 40035928 | | G | T | 0.810 | 0.190 | 0.015 (0.010 , 0.020) |
| 1:41236837_CT_C | NFYC | 1 | 40771165 | | 41236837 | | CT | C | 0.775 | 0.225 | -0.014 (-0.019 , -0.009) |
| rs41269079 | BEST4 | 1 | 44786343 | | 45252015 | | T | A | 0.811 | 0.189 | -0.015 (-0.020 , -0.010) |
| rs139795227 | RPAP2 | 1 | 92376810 | | 92842367 | | A | C | 0.986 | 0.014 | -0.059 (-0.075 , -0.042) |
| rs4498805 | SLC16A4 | 1 | 110367775 | | 110910397 | | G | T | 0.453 | 0.453 | -0.015 (-0.019 , -0.011) |
| rs11579626 | CHD1L | 1 | 147270300 | | 146741960 | | A | C | 0.915 | 0.085 | -0.026 (-0.033 , -0.020) |
| rs61818036 | PSMB4 | 1 | 151391723 | | 151364199 | | G | A | 0.176 | 0.176 | 0.020 (0.015 , 0.025) |
| rs932002 | PARP1 | 1 | 226389605 | | 226577306 | | C | T | 0.849 | 0.151 | 0.040 (0.035 , 0.046) |
| rs9752694 | SMC6 | 2 | 17692910 | | 17874177 | | C | G | 0.571 | 0.429 | 0.014 (0.010 , 0.018) |
| rs56178008 | TRMT61B | 2 | 28875677 | | 29098543 | | T | A | 0.563 | 0.437 | -0.014 (-0.018 , -0.010) |
| rs12615793 | ACYP2 | 2 | 54248777 | | 54475914 | | G | A | 0.860 | 0.140 | -0.043 (-0.049 , -0.037) |
| rs12613375 | LINC01122 | 2 | 58756974 | | 58984109 | | C | T | 0.862 | 0.138 | -0.018 (-0.023 , -0.012) |
| rs35671754 | ATIC | 2 | 215356147 | | 216220870 | | G | T | 0.704 | 0.296 | -0.013 (-0.017 , -0.008) |
| rs869785 | THRB | 3 | 24306309 | | 24347800 | | T | C | 0.328 | 0.328 | 0.015 (0.011 , 0.019) |
| rs78491606 | SHQ1 | 3 | 72842396 | | 72891547 | | A | C | 0.982 | 0.018 | 0.074 (0.060 , 0.089) |
| rs13062095 | SENP7 | 3 | 101548541 | | 101267385 | | T | C | 0.672 | 0.328 | -0.014 (-0.018 , -0.010) |
| rs6776756 | GATA2 | 3 | 128496978 | | 128215821 | | G | A | 0.402 | 0.402 | 0.015 (0.011 , 0.019) |
| rs2811491 |  | 3 | 128635107 | | 128353950 | | C | A | 0.392 | 0.392 | -0.012 (-0.016 , -0.008) |
| rs41272947 |  | 3 | 160401737 | | 160119525 | | G | A | 0.458 | 0.458 | 0.016 (0.012 , 0.020) |
| rs2293607 | TERC | 3 | 169764547 | | 169482335 | | T | C | 0.758 | 0.242 | 0.080 (0.071 , 0.089) |
| rs146546514 | | 3 | 169835282 | | 169553070 | | C | A | 0.983 | 0.017 | -0.057 (-0.073 , -0.041) |
| rs871134 | CCDC96 | 4 | 7042653 | | 7044380 | | C | T | 0.431 | 0.431 | 0.018 (0.014 , 0.022) |
| rs13129697 | SLC2A9 | 4 | 9925343 | | 9926967 | | T | G | 0.721 | 0.279 | -0.018 (-0.022 , -0.013) |
| rs4695407 | OCIAD1 | 4 | 48841355 | | 48843372 | | A | G | 0.492 | 0.492 | -0.014 (-0.017 , -0.010) |
| rs4435700 | NAF1 | 4 | 163099022 | | 164020174 | | C | A | 0.235 | 0.235 | -0.038 (-0.043 , -0.032) |
| rs113580095 | | 4 | 163144568 | | 164065720 | | A | G | 0.998 | 0.002 | 0.338 (0.293 , 0.383) |
| rs72631678 |  | 4 | 163181563 | | 164102715 | | G | A | 0.588 | 0.412 | -0.019 (-0.024 , -0.015) |
| rs9990898 |  | 4 | 163227972 | | 164149124 | | T | C | 0.857 | 0.143 | -0.022 (-0.027 , -0.016) |
| rs138895564 | TERT | 5 | 1271959 | | 1272074 | | C | T | 0.991 | 0.009 | -0.192 (-0.214 , -0.171) |
| rs7705526 |  | 5 | 1285859 | | 1285974 | | C | A | 0.673 | 0.327 | -0.053 (-0.059 , -0.048) |
| rs112290073 | | 5 | 1285917 | | 1286032 | | G | A | 0.988 | 0.012 | -0.080 (-0.099 , -0.061) |
| rs2853677 |  | 5 | 1287079 | | 1287194 | | G | A | 0.424 | 0.424 | 0.030 (0.025 , 0.035) |
| rs61748181 |  | 5 | 1294051 | | 1294166 | | C | T | 0.971 | 0.029 | 0.059 (0.048 , 0.071) |
| rs33987166 |  | 5 | 1296643 | | 1296758 | | T | C | 0.975 | 0.025 | -0.044 (-0.057 , -0.030) |
| 5:1303867_CT_C | | 5 | 1303752 | | 1303867 | | CT | C | 0.995 | 0.005 | -0.099 (-0.129 , -0.070) |
| rs79717857 |  | 5 | 1319882 | | 1319997 | | C | A | 0.974 | 0.026 | -0.060 (-0.073 , -0.047) |
| rs115251750 | | 5 | 1349749 | | 1349864 | | G | A | 0.965 | 0.035 | -0.047 (-0.059 , -0.036) |
| rs72801474 | HSPA4 | 5 | 133108436 | | 132444128 | | G | A | 0.912 | 0.088 | 0.020 (0.014 , 0.027) |
| rs34255404 | UBE2D2 | 5 | 139555995 | | 138935580 | | G | A | 0.943 | 0.057 | -0.038 (-0.046 , -0.030) |
| rs80324517 | LOC285766 | 6 | 204031 | | 204031 | | G | A | 0.952 | 0.048 | -0.040 (-0.049 , -0.031) |
| rs154979 | HLA-DMB | 6 | 32923162 | | 32890939 | | C | A | 0.971 | 0.029 | -0.038 (-0.049 , -0.026) |
| rs9398196 | CCDC162P | 6 | 109280351 | | 109601554 | | A | G | 0.480 | 0.480 | 0.015 (0.011 , 0.019) |
| rs13230646 | STK31 | 7 | 23890697 | | 23930316 | | T | C | 0.751 | 0.249 | 0.018 (0.013 , 0.022) |
| rs11769630 | IKZF1 | 7 | 50218107 | | 50257703 | | T | A | 0.928 | 0.072 | 0.026 (0.018 , 0.033) |
| rs2538745 | UPK3B | 7 | 76681467 | | 76310784 | | T | C | 0.397 | 0.397 | 0.012 (0.008 , 0.016) |
| rs2056726 | STAG3 | 7 | 100182660 | | 99780283 | | G | A | 0.786 | 0.214 | 0.024 (0.019 , 0.028) |
| rs609953 | RNU6-2 | 7 | 123782390 | | 123422444 | | T | A | 0.618 | 0.382 | -0.012 (-0.016 , -0.008) |
| rs7790856 | POT1 | 7 | 124819798 | | 124459852 | | C | T | 0.711 | 0.289 | 0.033 (0.027 , 0.038) |
| rs117811540 | | 7 | 124929862 | | 124569916 | | G | A | 0.992 | 0.008 | -0.138 (-0.161 , -0.115) |
| rs4731541 | TNPO3 | 7 | 129038182 | | 128678236 | | C | G | 0.375 | 0.375 | 0.021 (0.017 , 0.025) |
| rs11556924 | ZC3HC1 | 7 | 130023656 | | 129663496 | | C | T | 0.624 | 0.376 | -0.012 (-0.016 , -0.008) |
| rs138061125 | VIPR2 | 7 | 159286503 | | 159079192 | | G | A | 0.970 | 0.030 | -0.043 (-0.054 , -0.032) |
| rs1985369 |  | 7 | 159326530 | | 159119220 | | A | G | 0.132 | 0.132 | 0.032 (0.026 , 0.037) |
| rs2306646 | XPO7 | 8 | 21989075 | | 21846586 | | G | C | 0.441 | 0.441 | 0.021 (0.017 , 0.025) |
| rs762679 | MCM4 | 8 | 47972876 | | 48885436 | | T | A | 0.143 | 0.143 | -0.031 (-0.036 , -0.025) |
| rs7012816 | PRDM14 | 8 | 70052508 | | 70964743 | | G | A | 0.870 | 0.130 | -0.018 (-0.024 , -0.013) |
| rs10112752 | TERF1 | 8 | 73046483 | | 73958718 | | G | A | 0.570 | 0.430 | 0.028 (0.024 , 0.032) |
| rs1023767 | VIRMA | 8 | 94518741 | | 95530969 | | G | A | 0.762 | 0.238 | 0.019 (0.014 , 0.023) |
| rs11557154 | DCAF12 | 9 | 34107507 | | 34107505 | | C | T | 0.870 | 0.130 | 0.035 (0.029 , 0.041) |
| rs4743037 | ZNF462 | 9 | 106877689 | | 109639970 | | C | T | 0.769 | 0.231 | -0.014 (-0.019 , -0.010) |
| rs12572897 | NOC3L | 10 | 94355078 | | 96114835 | | G | A | 0.870 | 0.130 | 0.032 (0.026 , 0.038) |
| 10:101274251_CT_C | NKX2-3 | 10 | 99514494 | | 101274251 | | CT | C | 0.620 | 0.380 | 0.021 (0.017 , 0.025) |
| rs11190184 |  | 10 | 99608442 | | 101368199 | | G | C | 0.716 | 0.284 | 0.017 (0.013 , 0.021) |
| rs4919611 | PPRC1 | 10 | 102135182 | | 103894939 | | C | A | 0.113 | 0.113 | 0.021 (0.015 , 0.027) |
| rs10748858 | STN1 (OBFC1) | 10 | 103879756 | | 105639514 | | T | G | 0.595 | 0.405 | -0.027 (-0.031 , -0.023) |
| rs9419958 |  | 10 | 103916188 | | 105675946 | | T | C | 0.139 | 0.139 | 0.068 (0.062 , 0.074) |
| rs182641927 | | 10 | 104045281 | | 105805039 | | C | T | 0.996 | 0.004 | -0.102 (-0.137 , -0.068) |
| rs939916 | ODF3 | 11 | 202253 | | 202253 | | G | A | 0.330 | 0.330 | -0.025 (-0.029 , -0.020) |
| rs1609812 | HBB | 11 | 5225911 | | 5247141 | | G | A | 0.160 | 0.160 | -0.045 (-0.050 , -0.040) |
| rs10840270 | WEE1 | 11 | 9608006 | | 9629553 | | C | G | 0.344 | 0.344 | -0.014 (-0.018 , -0.010) |
| rs2293579 | PSMC3 | 11 | 47419207 | | 47440758 | | G | A | 0.614 | 0.386 | 0.013 (0.009 , 0.017) |
| rs141379009 | ATM | 11 | 108278480 | | 108149207 | | T | G | 0.974 | 0.026 | 0.062 (0.048 , 0.076) |
| rs611646 |  | 11 | 108306370 | | 108177097 | | T | A | 0.591 | 0.409 | 0.027 (0.022 , 0.032) |
| rs10845387 | LINC01252 | 12 | 11604809 | | 11757743 | | G | A | 0.647 | 0.353 | 0.014 (0.009 , 0.018) |
| rs12369950 | LINC00477 | 12 | 24609175 | | 24762109 | | T | C | 0.859 | 0.141 | 0.017 (0.011 , 0.022) |
| rs1907702 | KITLG | 12 | 88561692 | | 88955469 | | G | A | 0.233 | 0.233 | -0.015 (-0.019 , -0.010) |
| rs10774625 | SH2B3 | 12 | 111472415 | | 111910219 | | A | G | 0.476 | 0.476 | -0.016 (-0.020 , -0.012) |
| rs76666449 | SRSF9 | 12 | 120467092 | | 120904895 | | T | C | 0.899 | 0.101 | -0.029 (-0.035 , -0.022) |
| rs4758644 | ZCCHC8 | 12 | 122459368 | | 122943915 | | A | C | 0.269 | 0.269 | 0.016 (0.012 , 0.021) |
| rs1727302 | MPHOSPH9 | 12 | 123148383 | | 123632930 | | G | A | 0.259 | 0.259 | -0.019 (-0.024 , -0.015) |
| rs1332941 | KBTBD6 | 13 | 41120964 | | 41695100 | | A | G | 0.180 | 0.180 | -0.026 (-0.031 , -0.021) |
| rs35017269 | DIS3 | 13 | 72766039 | | 73340177 | | G | A | 0.984 | 0.016 | -0.066 (-0.082 , -0.050) |
| rs3093888 | TEP1 | 14 | 20344792 | | 20812951 | | G | A | 0.949 | 0.051 | 0.029 (0.021 , 0.038) |
| rs73581419 | RAB2B | 14 | 21472989 | | 21941148 | | C | T | 0.893 | 0.107 | -0.023 (-0.029 , -0.016) |
| rs12884911 | PPP1R36 | 14 | 64561153 | | 65027871 | | C | T | 0.497 | 0.497 | 0.012 (0.008 , 0.015) |
| rs762810 | MAX | 14 | 65077649 | | 65544367 | | C | A | 0.648 | 0.352 | 0.019 (0.015 , 0.023) |
| rs137901416 | DCAF4 | 14 | 72951387 | | 73418095 | | G | A | 0.900 | 0.100 | -0.039 (-0.046 , -0.033) |
| rs1007934 |  | 14 | 72996771 | | 73463479 | | G | A | 0.595 | 0.405 | 0.020 (0.016 , 0.024) |
| rs1957937 | TCL1A | 14 | 95715023 | | 96181360 | | A | T | 0.840 | 0.160 | -0.021 (-0.026 , -0.015) |
| rs17677991 | MGA | 15 | 41740185 | | 42032383 | | C | G | 0.658 | 0.342 | -0.022 (-0.026 , -0.018) |
| rs1980240 | TEX9 | 15 | 56481820 | | 56774018 | | A | C | 0.595 | 0.405 | -0.012 (-0.016 , -0.009) |
| rs5742915 | PML | 15 | 74044292 | | 74336633 | | T | C | 0.554 | 0.446 | -0.019 (-0.023 , -0.015) |
| rs80116508 | SLX4 | 16 | 3600969 | | 3650970 | | G | A | 0.938 | 0.062 | 0.035 (0.027 , 0.043) |
| rs11646283 | USP7 | 16 | 8979203 | | 9073060 | | T | C | 0.589 | 0.411 | -0.015 (-0.019 , -0.011) |
| rs182059586 | PARN | 16 | 14558363 | | 14652220 | | T | C | 0.975 | 0.025 | 0.056 (0.043 , 0.069) |
| rs8053839 | LONP2 | 16 | 48356601 | | 48390512 | | G | T | 0.458 | 0.458 | 0.014 (0.010 , 0.018) |
| rs76219171 |  | 16 | 50155018 | | 50188929 | | G | A | 0.942 | 0.058 | -0.034 (-0.042 , -0.025) |
| rs28711261 | ACD | 16 | 67583283 | | 67617186 | | A | G | 0.867 | 0.133 | -0.020 (-0.026 , -0.014) |
| rs139438549 | | 16 | 67658960 | | 67692863 | | T | C | 0.999 | 0.001 | -0.259 (-0.317 , -0.201) |
| rs142507451 | | 16 | 67660141 | | 67694044 | | C | T | 0.997 | 0.003 | 0.156 (0.121 , 0.192) |
| rs529549411 | | 16 | 70356607 | | 70390510 | | C | T | 0.994 | 0.006 | 0.104 (0.074 , 0.133) |
| rs34003787 | ZFHX3 | 16 | 73037482 | | 73071381 | | C | T | 0.912 | 0.088 | 0.024 (0.017 , 0.031) |
| rs183553155 | RFWD3 | 16 | 74457341 | | 74491239 | | G | A | 0.989 | 0.011 | -0.083 (-0.102 , -0.063) |
| rs11866592 |  | 16 | 74620498 | | 74654396 | | G | A | 0.858 | 0.142 | -0.028 (-0.034 , -0.022) |
| rs7193541 |  | 16 | 74630845 | | 74664743 | | T | C | 0.583 | 0.417 | 0.017 (0.013 , 0.021) |
| rs2303262 | MPHOSPH6 | 16 | 82170153 | | 82203758 | | C | T | 0.223 | 0.223 | 0.047 (0.043 , 0.052) |
| rs35216338 |  | 16 | 88069404 | | 88103010 | | C | T | 0.960 | 0.040 | -0.032 (-0.042 , -0.022) |
| rs9923119 | PRDM7 | 16 | 90087407 | | 90153815 | | T | C | 0.750 | 0.250 | -0.018 (-0.023 , -0.014) |
| rs5030755 |  | 17 | 1879658 | | 1782952 | | A | G | 0.887 | 0.113 | -0.025 (-0.031 , -0.019) |
| rs4724 | CTC1 | 17 | 7857079 | | 7760397 | | G | A | 0.883 | 0.117 | 0.056 (0.050 , 0.062) |
| rs75664430 |  | 17 | 8161461 | | 8064779 | | C | G | 0.752 | 0.248 | 0.024 (0.020 , 0.029) |
| rs111527438 | ADAP2 | 17 | 30925685 | | 29252703 | | T | C | 0.649 | 0.351 | -0.012 (-0.016 , -0.008) |
| rs12941945 |  | 17 | 43370860 | | 41448228 | | A | G | 0.832 | 0.168 | 0.022 (0.016 , 0.027) |
| rs2069536 | TEN1 | 17 | 76005025 | | 74001106 | | A | G | 0.250 | 0.250 | 0.015 (0.010 , 0.019) |
| rs1143697 | TK1 | 17 | 78182667 | | 76178748 | | T | C | 0.537 | 0.463 | -0.017 (-0.021 , -0.013) |
| rs144204502 | | 17 | 78187152 | | 76183233 | | C | T | 0.987 | 0.013 | 0.113 (0.096 , 0.131) |
| rs9952504 | TYMS | 18 | 657458 | | 657458 | | A | G | 0.958 | 0.042 | 0.038 (0.027 , 0.048) |
| rs3891167 |  | 18 | 658423 | | 658423 | | A | G | 0.747 | 0.253 | 0.026 (0.021 , 0.032) |
| rs111811424 | | 18 | 674579 | | 674579 | | C | T | 0.914 | 0.086 | -0.046 (-0.053 , -0.038) |
| rs2741181 |  | 18 | 689437 | | 689437 | | C | T | 0.913 | 0.087 | 0.032 (0.024 , 0.039) |
| rs78694226 |  | 18 | 710980 | | 710980 | | G | A | 0.992 | 0.008 | 0.070 (0.048 , 0.092) |
| rs79824385 | LINC01478 | 18 | 44402414 | | 41982379 | | T | A | 0.870 | 0.130 | -0.029 (-0.035 , -0.023) |
| rs8088824 |  | 18 | 44571296 | | 42151261 | | C | T | 0.237 | 0.237 | -0.025 (-0.029 , -0.020) |
| rs2276182 | POLI | 18 | 54271677 | | 51798047 | | C | G | 0.597 | 0.403 | -0.023 (-0.027 , -0.019) |
| rs6565924 | ZNF236 | 18 | 76979269 | | 74691225 | | A | G | 0.639 | 0.361 | -0.013 (-0.017 , -0.009) |
| rs80337039 | MAP2K2 | 19 | 4105091 | | 4105089 | | G | T | 0.994 | 0.006 | -0.084 (-0.111 , -0.058) |
| rs35601737 | TRMT1 | 19 | 13109889 | | 13220703 | | C | G | 0.704 | 0.296 | 0.014 (0.010 , 0.018) |
| rs8105767 | ZNF208 | 19 | 22032639 | | 22215441 | | A | G | 0.705 | 0.295 | -0.030 (-0.035 , -0.026) |
| rs4530278 | CEBPA | 19 | 33262088 | | 33752994 | | G | T | 0.402 | 0.402 | -0.014 (-0.018 , -0.010) |
| rs429358 | APOE | 19 | 44908684 | | 45411941 | | T | C | 0.846 | 0.154 | -0.017 (-0.023 , -0.012) |
| rs11084431 | ZSCAN5B | 19 | 56197298 | | 56708667 | | G | A | 0.396 | 0.396 | 0.013 (0.008 , 0.017) |
| rs8102497 | PEG3 | 19 | 56858687 | | 57370055 | | G | A | 0.568 | 0.432 | 0.015 (0.011 , 0.019) |
| rs1291143 | SAMHD1 | 20 | 36897237 | | 35525640 | | A | C | 0.151 | 0.151 | -0.040 (-0.046 , -0.034) |
| rs6030416 |  | 20 | 36956221 | | 35584624 | | T | G | 0.136 | 0.136 | 0.028 (0.022 , 0.033) |
| rs2259797 |  | 20 | 63640895 | | 62272248 | | T | C | 0.907 | 0.093 | 0.036 (0.029 , 0.043) |
| rs41308088 |  | 20 | 63661765 | | 62293118 | | C | T | 0.919 | 0.081 | -0.073 (-0.081 , -0.065) |
| rs187013287 | | 20 | 63667021 | | 62298374 | | A | T | 0.997 | 0.003 | 0.255 (0.213 , 0.298) |
| rs8114049 |  | 20 | 63679453 | | 62310806 | | C | T | 0.331 | 0.331 | 0.058 (0.052 , 0.063) |
| rs35640778 |  | 20 | 63689775 | | 62321128 | | G | A | 0.979 | 0.021 | 0.236 (0.222 , 0.250) |
| 20:62321690_GAGA_G | | 20 | 63690337 | | 62321690 | | GAGA | G | 0.996 | 0.004 | -0.108 (-0.143 , -0.073) |
| rs115610405 | | 20 | 63694480 | | 62325833 | | C | A | 0.981 | 0.019 | 0.065 (0.050 , 0.080) |
| rs55765053 |  | 20 | 63697127 | | 62328480 | | C | T | 0.934 | 0.066 | -0.038 (-0.046 , -0.029) |
| rs3761121 |  | 20 | 63711343 | | 62342695 | | T | C | 0.878 | 0.122 | -0.037 (-0.043 , -0.030) |
| rs187577818 | | 20 | 63727517 | | 62358869 | | A | C | 0.997 | 0.003 | -0.297 (-0.339 , -0.256) |
| rs111527478 | | 20 | 64046747 | | 62678100 | | G | A | 0.900 | 0.100 | -0.023 (-0.030 , -0.017) |
| rs28502153 | GAB4 | 22 | 16988159 | | 17469049 | | C | A | 0.622 | 0.378 | 0.021 (0.017 , 0.025) |
| SNPs included in the primary LTL PRS calculation. Columns show gene, chromosome, base-pair positions (GRCh38 and GRCh37), effect allele (A1), effect allele frequency (FREQ(A1)), minor allele frequency (MAF), and effect size (Beta) with 95% confidence intervals. * Statistics from Codd V, et al. Polygenic basis and biomedical consequences of telomere length variation. Nat Genet. 2021;53(10):1425-1433. doi:10.1038/s41588-021-00944-6 | | | | | | | | | | | |

| **Table S2. Information on the Proxy SNPs Included in the Sensitivity PRS Calculation** | | | | |
| --- | --- | --- | --- | --- |
| **RSID for missing SNPs** | **Proxy RSID*** | **Chr** | **Position (GRCh38)** ‡ | **MAF (EA)** |
| rs113206288 | rs536114760 | 5 | [1610790](http://genome.ucsc.edu/cgi-bin/hgTracks?db=hg38&position=chr5:1610739-1610839) | 0.13 |
| rs4742448 | rs4740925 | 9 | [826584](http://genome.ucsc.edu/cgi-bin/hgTracks?db=hg38&position=chr9:826533-826633) | 0.473 |
| rs181647350 | rs145723797 | 15 | [50089272](http://genome.ucsc.edu/cgi-bin/hgTracks?db=hg38&position=chr15:50089221-50089321) | 0.222 |
| rs12447324 | [rs6500280](http://www.ncbi.nlm.nih.gov/snp/rs6500280) | 16 | [50066363](http://genome.ucsc.edu/cgi-bin/hgTracks?db=hg38&position=chr16:50066312-50066412) | 0.173 |
| rs62053340 | rs62050645 | 16 | [69980912](http://genome.ucsc.edu/cgi-bin/hgTracks?db=hg38&position=chr16:69980861-69980961) | 0.405 |
| rs62046862 | rs12922388 | 16 | [88040093](http://genome.ucsc.edu/cgi-bin/hgTracks?db=hg38&position=chr16:88040042-88040142) | 0.366 |
| rs7218033 | rs7217857 | 17 | [1790848](http://genome.ucsc.edu/cgi-bin/hgTracks?db=hg38&position=chr17:1790797-1790897) | 0.25 |
| rs62079650 | rs62079652 | 17 | [43323901](http://genome.ucsc.edu/cgi-bin/hgTracks?db=hg38&position=chr17:43323850-43323950) | 0.263 |
| rs1879100 | rs1879102 | 18 | [80227876](http://genome.ucsc.edu/cgi-bin/hgTracks?db=hg38&position=chr18:80227825-80227925) | 0.148 |
| *SNP information on the 9 proxies added to the PRS calculation in the sensitivity analysis. ‡Chromosome and position (GRCh38) correspond to the proxy SNPs. | | | | |

**Figure S2.1 LTL Proteomics Analysis Participants Exclusion Diagram**

Atherosclerosis Risk in Communities Population at Baseline N = 15,792

Missing Telomere Length Estimates, N = 2,716

Participants not available at Visit 2, N = 1,444

Missing plasma protein data, covariates,
or protein levels were off 6 SD from the mean, N = 1,673

Final Sample Size: N = 9,723

Samples not at Visit 1, 2, or 3, N = 216

**Figure S2.2 LTL PRS Proteomics Analysis Participants Exclusion Diagram**

Atherosclerosis Risk in Communities Population at Baseline N = 15,792

Missing Any Information on Covariates or Protein Levels, or protein levels were off 6 SD from the mean: N = 2,538

Missing Genetic Data N = 3,573

Final Sample Size: N = 9,681

**Figure S2.3 LTL and AAA Association Analysis Participants Exclusion Diagram**

Atherosclerosis Risk in Communities Population at Baseline N = 15,792

Missing Telomere Length Estimates N = 2,661

Exclude AAA cases prior corresponding LTL measurement visit (N=51)

Missing Any Information on Covariates N = 981

Final Sample Size: N = 12,099

| **Table S3. Baseline Characteristics of Participants in the LTL-Proteomics and LTL-AAA Analyses: Mean (SD) or n (%)** | | | | | | | | | |  |
| --- | --- | --- | --- | --- | --- | --- | --- | --- | --- | --- |
|  | **LTL Proteomics Analysis in ARIC and CHS *** | | | | |  | **LTL AAA Association Analysis in ARIC** | | | |
|  | **ARIC White Participants (N=8,055)** | **ARIC Black Participants (N=1,668)** | | **CHS White Participants (WGS) (N=2,431)** | | **CHS White Participants (Southern blot) (N=1,053)** | **Incident AAA(N=609)** | | **Non-AAA (N=11,490)** | |
| Age, years | 57.8 (5.9) | 56.7 (5.9) | | 74.7 (4.8) | | 74.7 (4.8) | 59.1 (5.5) | | 57.4 (6.0) | |
| Female, n (%) | 4261 (53%) | 1038 (62%) | | 1445 (59%) | | 645 (61%) | 185 (30%) | | 6529 (57%) | |
| eGFR,mL/min/1.73 m^2^, | 96.5 (12.9) | 91.5 (17.7) | | 72.4 (17.4) | | 70.9 (17.8) | 95.9 (13.6) | | 95.7 (14.0) | |
| Smoking Status, n (%) |  |  | |  | |  |  | |  | |
| Current Smoking | 1706 (21.2%) | 445 (26.7%) | | 219 (9%) | | 116 (11%) | 269 (44.2%) | | 2366 (20.6%) | |
| Former Smoking | 3306 (41.1%) | 508 (30.5%) | | 1118 (46%) | | 495 (47%) | 246 (40.4%) | | 4410 (38.4%) | |
| Never Smoking | 3043 (37.8%) | 715 (42.9%) | | 1094 (45%) | | 442 (42%) | 94 (15.4%) | | 4714 (41.0%) | |
| BMI, kg/m2 | 27.5 (4.9) | 30.2 (6.3) | | 26.4 (4.4) | | 26.6 (4.4) | 27.4 (4.3) | | 28.0 (5.5) | |
| Height, cm | 169 (9.4) | 168 (8.9) | | 164.2 (9.6) | | 163.7 (9.6) | 172 (8.7) | | 168 (9.3) | |
| Diabetes, n (%) | 968 (12.0%) | 442 (26.5%) | | 310 (13%) | | 143 (14%) | 62 (10.2%) | | 1756 (15.3%) | |
| Hypertension, n (%) | 2445 (30.4%) | 942 (56.5%) | | 1571 (65%) | | 701 (67%) | 229 (37.6%) | | 4108 (35.8%) | |
| *The LTL proteomics analysis treated ARIC white participants as the discovery set and ARIC black participants and CHS white participants as replication sets.  Abbreviations: AAA, abdominal aortic aneurysm; ARIC, Atherosclerosis Risk in Communities Study; BMI, body mass index, CHS, Cardiovascular Health Study; eGFR, estimated glomerular filtration rate; LTL, leukocyte telomere length; N, number of participants; PRS, polygenic risk score; WGS, whole genome sequencing. | | | | | | | | | | |
|  |  |  |  |  |  |  |  |  |  |  |
| **Table S4. Baseline Characteristics of Participants Included in the LTL PRS Proteomics Analysis in ARIC and CHS: Mean (SD) or n (%)** | | | | | | | | | |  |
|  | | | **LTL PRS Proteomics Analysis in ARIC and CHS** | | | | | | |  |
|  | | | **ARIC White Participants (N=7,587)** | | **ARIC Black Participants (N=2,094)** | | | **CHS White Participants (N=2,333)** | |  |
| Age, years | | | 57.2 (5.68) | | 56.5 (5.76) | | | 74.8 (4.9) | |  |
| Female, n (%) | | | 4048 (53.4%) | | 1307 (62.4%) | | | 1390 (59.6%) | |  |
| eGFR, mL/min/1.73 m^2^, | | | 94.1 (13.2) | | 103 (20.7) | | | 72.4 (17.6) | |  |
| Smoking Status, n (%) | | |  | |  | | |  | |  |
| Current Smoking | | | 1625 (21.4%) | | 567 (27.1%) | | | 211 (9.1%) | |  |
| Former Smoking | | | 3082 (40.6%) | | 644 (30.8%) | | | 1078 (46.2%) | |  |
| Never Smoking | | | 2880 (38.0%) | | 883 (42.2%) | | | 1044 (44.8%) | |  |
| BMI, kg/m2 | | | 27.4 (4.94) | | 30.1 (6.14) | | | 26.4 (4.4) | |  |
| Height, cm | | | 169 (9.45) | | 168 (8.99) | | | 164 (9.6) | |  |
| Diabetes, n (%) | | | 823 (10.8%) | | 461 (22.0%) | | | 294 (12.7%) | |  |
| Hypertension, n (%) | | | 2155 (28.4%) | | 956 (45.7%) | | | 1507 (64.6%) | |  |
| Abbreviations: ARIC, Atherosclerosis Risk in Communities Study; BMI, body mass index, CHS, Cardiovascular Health Study; eGFR, estimated glomerular filtration rate; LTL: leukocyte telomere length; N, number of participants; PRS, polygenic risk score | | | | | | | | | |  |
|  |  |  |  |  |  |  |  |  |  |  |

| **Table S5. Significant Proteins in the Primary LTL Proteomics Analysis and Sensitivity Analysis in ARIC White Participants** **§** | | | | | | |  |
| --- | --- | --- | --- | --- | --- | --- | --- |
|  |  |  | **Significant Proteins in the Primary LTL Proteomics Analysis** | | **Sensitivity Analysis§** | |  |
| **Aptamer ID** | **Protein Name** | **Gene Symbol** | **Estimate (95%CI)** | **P** | **Estimate (95%CI)** | **P** |  |
| SeqId_4374_45 | Growth/differentiation factor 15‡ | GDF15 | -0.072 (-0.092, -0.053) | 7.97E-13 | -0.074 (-0.095, -0.053) | 5.33E-12 |  |
| SeqId_4990_87 | Platelet glycoprotein Ib alpha chain | GP1Bα | 0.071 (0.050, 0.091) | 1.55E-11 | 0.076 (0.054, 0.098) | 1.32E-11 |  |
| SeqId_11212_7 | Thioredoxin domain-containing protein 5 | TXNDC5 | -0.053 (-0.072, -0.034) | 7.84E-08 | -0.049 (-0.069, -0.028) | 3.28E-06 |  |
| SeqId_5947_90 | Thrombopoietin‡ | THPO | -0.055 (-0.076, -0.035) | 9.05E-08 | -0.052 (-0.074, -0.031) | 2.06E-06 |  |
| SeqId_2665_26 | Tumor necrosis factor receptor superfamily member 17 | TNFRSF17 | -0.054 (-0.075, -0.034) | 2.56E-07 | -0.054 (-0.076, -0.032) | 1.66E-06 |  |
| SeqId_16322_10 | Marginal zone B- and B1-cell-specific protein* | MZB1* | -0.056 (-0.078, -0.034) | 4.88E-07 | -0.055 (-0.078, -0.032) | 4.25E-06 |  |
| SeqId_10612_18 | Procollagen-lysine,2-oxoglutarate 5-dioxygenase 3*‡ | PLOD3* | -0.056 (-0.078, -0.034) | 4.90E-07 | -0.056 (-0.079, -0.032) | 3.02E-06 |  |
| SeqId_8958_51 | Neural cell adhesion molecule L1-like protein‡ | CHL1 | -0.054 (-0.076, -0.032) | 1.50E-06 | -0.054 (-0.078, -0.030) | 8.36E-06 |  |
| SeqId_10702_1 | Collagen alpha-1 (XXVIII) chain* | COL28A1* | -0.043 (-0.060, -0.025) | 2.97E-06 | -0.046 (-0.065, -0.027) | 2.64E-06 |  |
| § Sensitivity analysis additionally adjusted for leisure-time physical activity (measured in MET-minutes/week), prevalent coronary heart disease, prevalent diabetes, and alcohol consumption in ARIC White participants.  Effect estimates and p-values for the associations between leukocyte telomere length (LTL) and nine significant proteins identified in the primary LTL proteomics analysis. * Protein replicated in ARIC Black participants. ‡Protein replicated in CHS White participants (p < 0.011; one-sided Bonferroni correction for testing 9 proteins). | | | | | | |  |
|  |  |  |  |  |  |  |  |

| **Table S6. Visit 3 Replication of Eight LTL PRS-Protein Associations in ARIC White Participants Identified at Visit 2 in ARIC White Participants** | | | | | | | | | |  |
| --- | --- | --- | --- | --- | --- | --- | --- | --- | --- | --- |
| **Aptamer ID** | **Estimate** | **Std. Error** | **t-Statistic** | **P** | **Lower 95% CI** | **Upper 95% CI** | **N** | **Gene Symbol** | **Protein Name** |  |
| SeqId_5947_90 | -0.038 | 0.010 | -3.613 | 3.04E-04 | -0.058 | -0.017 | 7557 | THPO | Thrombopoietin |  |
| SeqId_4990_87 | 0.033 | 0.011 | 3.008 | 2.64E-03 | 0.011 | 0.054 | 7579 | GP1Bα | Platelet glycoprotein Ib alpha chain |  |
| SeqId_8275_31 | 0.037 | 0.011 | 3.257 | 1.13E-03 | 0.015 | 0.059 | 7582 | PEAR1 | Platelet endothelial aggregation receptor 1 |  |
| SeqId_6947_4 | 0.041 | 0.010 | 4.143 | 3.46E-05 | 0.022 | 0.060 | 7567 | ST3GAL6 | Type 2 lactosamine alpha-2,3-sialyltransferase |  |
| SeqId_3651_50 | 0.023 | 0.011 | 2.017 | 4.38E-02 | 0.001 | 0.046 | 7584 | KDR | Vascular endothelial growth factor receptor 2 |  |
| SeqId_11128_29 | 0.024 | 0.009 | 2.581 | 9.86E-03 | 0.006 | 0.043 | 7519 | TMEM132C | Transmembrane protein 132C |  |
| SeqId_7185_29 | 0.022 | 0.011 | 2.066 | 3.88E-02 | 0.001 | 0.044 | 7584 | GP5 | Platelet glycoprotein V |  |
| SeqId_11178_21 | -0.032 | 0.011 | -2.918 | 3.53E-03 | -0.054 | -0.011 | 7577 | SVEP1 | Sushi, von Willebrand factor type A, EGF and pentraxin domain-containing protein 1 |  |
| Replication of LTL PRS–protein associations at ARIC Visit 3 among White participants, for eight proteins originally identified at Visit 2 in the discovery analysis. Estimate: Effect estimates for the association between LTL PRS and each protein; Std. Error: Standard errors; N: Number of participants with available data for each protein. Abbreviations: SNP, single nucleotide polymorphism; PRS, polygenic risk score. | | | | | | | | | |  |
|  |  |  |  |  |  |  |  |  |  |  |

| **Table S7. Visit 3 Replication of Eight LTL PRS-Protein Associations in ARIC Black Participants Identified at Visit 2 in ARIC White Participants** | | | | | | | | | |  |
| --- | --- | --- | --- | --- | --- | --- | --- | --- | --- | --- |
| **Aptamer ID** | **Estimate** | **Std. Error** | **t-Statistic** | **P** | **Lower 95% CI** | **Upper 95% CI** | **N** | **Gene Symbol** | **Protein Name** |  |
| SeqId_5947_90 | -0.001 | 0.022 | -0.026 | 0.980 | -0.044 | 0.043 | 1731 | THPO | Thrombopoietin |  |
| SeqId_4990_87 | 0.012 | 0.025 | 0.486 | 0.627 | -0.037 | 0.062 | 1733 | GP1Bα | Platelet glycoprotein Ib alpha chain |  |
| SeqId_8275_31 | -0.020 | 0.025 | -0.835 | 0.404 | -0.069 | 0.028 | 1737 | PEAR1 | Platelet endothelial aggregation receptor 1 |  |
| SeqId_6947_4 | -0.013 | 0.023 | -0.595 | 0.552 | -0.058 | 0.031 | 1733 | ST3GAL6 | Type 2 lactosamine alpha-2,3-sialyltransferase |  |
| SeqId_3651_50 | 0.060 | 0.025 | 2.444 | 0.015 | 0.012 | 0.108 | 1737 | KDR | Vascular endothelial growth factor receptor 2 |  |
| SeqId_11128_29 | -0.030 | 0.020 | -1.487 | 0.137 | -0.069 | 0.009 | 1723 | TMEM132C | Transmembrane protein 132C |  |
| SeqId_7185_29 | 0.010 | 0.026 | 0.370 | 0.711 | -0.041 | 0.061 | 1737 | GP5 | Platelet glycoprotein V |  |
| SeqId_11178_21 | -0.041 | 0.026 | -1.603 | 0.109 | -0.091 | 0.009 | 1735 | SVEP1 | Sushi, von Willebrand factor type A, EGF and pentraxin domain-containing protein 1 |  |
| Replication of LTL PRS–protein associations at ARIC Visit 3 in black participants for eight proteins identified in the discovery analysis among ARIC Visit 2 White participants. Estimate: Effect estimates for the association between LTL PRS and each protein; Std. Error: Standard errors; N: Number of participants with available data for each protein. Abbreviations: SNP, single nucleotide polymorphism; PRS, polygenic risk score. | | | | | | | | | |  |
|  |  |  |  |  |  |  |  |  |  |  |

| **Table S8. Sensitivity Analysis of Eight LTL PRS–Protein Associations in ARIC White Participants at Visit 2 After Adding Nine Additional Proxy SNPs to the PRS** | | | | | | | | | |  |
| --- | --- | --- | --- | --- | --- | --- | --- | --- | --- | --- |
| **Aptamer ID** | **Estimate** | **Std. Error** | **t-Statistic** | **P** | **Lower.95%** | **Upper.95%** | **N** | **Gene Symbol** | **Protein Name** |  |
| SeqId_5947_90 | -0.051 | 0.010 | -5.099 | 3.490E-07 | -0.070 | -0.031 | 7561 | THPO | Thrombopoietin |  |
| SeqId_4990_87 | 0.051 | 0.011 | 4.741 | 2.160E-06 | 0.030 | 0.072 | 7581 | GP1Bα | Platelet glycoprotein Ib alpha chain |  |
| SeqId_8275_31 | 0.051 | 0.011 | 4.700 | 2.650E-06 | 0.030 | 0.072 | 7584 | PEAR1 | Platelet endothelial aggregation receptor 1 |  |
| SeqId_6947_4 | 0.045 | 0.010 | 4.666 | 3.130E-06 | 0.026 | 0.064 | 7569 | ST3GAL6 | Type 2 lactosamine alpha-2,3-sialyltransferase |  |
| SeqId_3651_50 | 0.054 | 0.011 | 4.969 | 6.880E-07 | 0.033 | 0.076 | 7587 | KDR | Vascular endothelial growth factor receptor 2 |  |
| SeqId_11128_29 | 0.038 | 0.009 | 4.311 | 1.650E-05 | 0.021 | 0.055 | 7536 | TMEM132C | Transmembrane protein 132C |  |
| SeqId_7185_29 | 0.046 | 0.011 | 4.267 | 2.000E-05 | 0.025 | 0.067 | 7586 | GP5 | Platelet glycoprotein V |  |
| SeqId_11178_21 | -0.051 | 0.011 | -4.749 | 2.080E-06 | -0.072 | -0.030 | 7584 | SVEP1 | Sushi, von Willebrand factor type A, EGF and pentraxin domain-containing protein 1 |  |
| Replication of LTL PRS–protein associations at ARIC Visit 2 among white participants, after adding nine additional proxy SNPs to the PRS. These associations involve eight proteins identified in the original Visit 2 discovery analysis. Estimate: Effect estimates for the association between LTL PRS and each protein; Std. Error: Standard errors; N: Number of participants with available data for each protein. Abbreviations: SNP, single nucleotide polymorphism; PRS, polygenic risk score. | | | | | | | | | |  |
|  |  |  |  |  |  |  |  |  |  |  |

| **Table S9. Sensitivity Analysis of Eight LTL PRS–Protein Associations in ARIC Black Participants at Visit 2 After Adding Nine Additional Proxy SNPs to the PRS** | | | | | | | | | |  |
| --- | --- | --- | --- | --- | --- | --- | --- | --- | --- | --- |
| **Aptamer ID** | **Estimate** | **Std. Error** | **t-Statistic** | **P** | **Lower.95%** | **Upper.95%** | **N** | **Gene Symbol** | **Protein Name** |  |
| SeqId_5947_90 | -0.023 | 0.022 | -1.085 | 2.78E-01 | -0.065 | 0.019 | 2090 | THPO | Thrombopoietin |  |
| SeqId_4990_87 | 0.005 | 0.024 | 0.211 | 8.33E-01 | -0.042 | 0.052 | 2092 | GP1Bα | Platelet glycoprotein Ib alpha chain |  |
| SeqId_8275_31 | -0.001 | 0.023 | -0.054 | 9.57E-01 | -0.047 | 0.044 | 2094 | PEAR1 | Platelet endothelial aggregation receptor 1 |  |
| SeqId_6947_4 | -0.017 | 0.021 | -0.815 | 4.15E-01 | -0.059 | 0.024 | 2089 | ST3GAL6 | Type 2 lactosamine alpha-2,3-sialyltransferase |  |
| SeqId_3651_50 | 0.058 | 0.024 | 2.445 | 1.46E-02 | 0.012 | 0.105 | 2093 | KDR | Vascular endothelial growth factor receptor 2 |  |
| SeqId_11128_29 | -0.026 | 0.018 | -1.402 | 1.61E-01 | -0.061 | 0.010 | 2077 | TMEM132C | Transmembrane protein 132C |  |
| SeqId_7185_29 | 0.039 | 0.026 | 1.493 | 1.36E-01 | -0.012 | 0.089 | 2094 | GP5 | Platelet glycoprotein V |  |
| SeqId_11178_21 | -0.004 | 0.023 | -0.169 | 8.66E-01 | -0.050 | 0.042 | 2089 | SVEP1 | Sushi, von Willebrand factor type A, EGF and pentraxin domain-containing protein 1 |  |
| Replication of LTL PRS–protein associations at ARIC Visit 2 in Black participants after adding nine additional proxy SNPs, based on eight proteins identified in the ARIC Visit 2 White discovery analysis. Estimate: Effect estimates for the association between LTL PRS and each protein; Std. Error: Standard errors; N: Number of participants with available data for each protein. Abbreviations: SNP, single nucleotide polymorphism; PRS, polygenic risk score. | | | | | | | | | |  |
|  |  |  |  |  |  |  |  |  |  |  |

| **Table S10. Forward Direction Mendelian Randomization (MR) Analysis Results** | | | | | | | | | | | |  |
| --- | --- | --- | --- | --- | --- | --- | --- | --- | --- | --- | --- | --- |
| **Exposure** | **Outcome** | **Aptamer ID** | **NSNP** | **Method** | **Estimate** | **Std. Error** | **Lower 95% CI** | **Upper 95% CI** | **P-value** | **R2*** | **F♰** |  |
| LTL | PLOD3 | 10612_18 | 89 | IVW | -0.017 | 0.036 | -0.088 | 0.053 | 0.635 | 0.028 | 149.924 |  |
| LTL | PLOD3 | 10612_18 | 89 | Weighted median | -0.018 | 0.055 | -0.126 | 0.089 | 0.737 | 0.028 | 149.924 |  |
| LTL | PLOD3 | 10612_18 | 89 | MR-Egger | -0.020 | 0.061 | -0.140 | 0.100 | 0.738 | 0.028 | 149.924 |  |
| LTL | PLOD3 | 10612_18 | 89 | (intercept) | 0.000 | 0.002 | -0.004 | 0.004 | 0.945 | 0.028 | 149.924 |  |
| LTL | PLOD3 | 10612_18 | 89 | Mode Based | -0.043 | 0.058 | -0.156 | 0.071 | 0.460 | 0.028 | 149.924 |  |
| LTL | PLOD3 | 10612_18 | 89 | MR-PRESSO (Raw) | -0.017 | 0.036 | -0.088 | 0.054 | 0.636 | 0.028 | 149.924 |  |
| LTL | PLOD3 | 10612_18 | 89 | MR-PRESSO (Outlier-Corrected) | No Outliers Detected | | | | | | |  |
| LTL | COL28A1 | 10702_1 | 89 | IVW | -0.011 | 0.036 | -0.082 | 0.061 | 0.769 | 0.028 | 149.924 |  |
| LTL | COL28A1 | 10702_1 | 89 | Weighted median | -0.088 | 0.055 | -0.195 | 0.020 | 0.109 | 0.028 | 149.924 |  |
| LTL | COL28A1 | 10702_1 | 89 | MR-Egger | -0.026 | 0.062 | -0.147 | 0.095 | 0.676 | 0.028 | 149.924 |  |
| LTL | COL28A1 | 10702_1 | 89 | (intercept) | 0.001 | 0.002 | -0.003 | 0.004 | 0.761 | 0.028 | 149.924 |  |
| LTL | COL28A1 | 10702_1 | 89 | Mode Based | -0.085 | 0.056 | -0.194 | 0.024 | 0.127 | 0.028 | 149.924 |  |
| LTL | COL28A1 | 10702_1 | 89 | MR-PRESSO (Raw) | -0.011 | 0.036 | -0.083 | 0.062 | 0.769 | 0.028 | 149.924 |  |
| LTL | COL28A1 | 10702_1 | 89 | MR-PRESSO (Outlier-Corrected) | No Outliers Detected | | | | | | |  |
| LTL | SVEP1 | 11178_21 | 89 | IVW | -0.075 | 0.033 | -0.140 | -0.010 | 0.024 | 0.028 | 149.924 |  |
| LTL | SVEP1 | 11178_21 | 89 | Weighted median | -0.097 | 0.053 | -0.202 | 0.007 | 0.067 | 0.028 | 149.924 |  |
| LTL | SVEP1 | 11178_21 | 89 | MR-Egger | -0.119 | 0.057 | -0.230 | -0.008 | 0.036 | 0.028 | 149.924 |  |
| LTL | SVEP1 | 11178_21 | 89 | (intercept) | 0.002 | 0.002 | -0.002 | 0.005 | 0.336 | 0.028 | 149.924 |  |
| LTL | SVEP1 | 11178_21 | 89 | Mode Based | -0.135 | 0.057 | -0.247 | -0.022 | 0.019 | 0.028 | 149.924 |  |
| LTL | SVEP1 | 11178_21 | 89 | MR-PRESSO (Raw) | -0.075 | 0.030 | -0.135 | -0.015 | 0.015 | 0.028 | 149.924 |  |
| LTL | SVEP1 | 11178_21 | 89 | MR-PRESSO (Outlier-Corrected) | No Outliers Detected | | | | | | |  |
| LTL | TXNDC5 | 11212_7 | 89 | IVW | 0.004 | 0.034 | -0.063 | 0.071 | 0.904 | 0.028 | 149.924 |  |
| LTL | TXNDC5 | 11212_7 | 89 | Weighted median | 0.003 | 0.055 | -0.104 | 0.110 | 0.960 | 0.028 | 149.924 |  |
| LTL | TXNDC5 | 11212_7 | 89 | MR-Egger | 0.087 | 0.058 | -0.026 | 0.200 | 0.131 | 0.028 | 149.924 |  |
| LTL | TXNDC5 | 11212_7 | 89 | (intercept) | -0.003 | 0.002 | -0.007 | 0.000 | 0.075 | 0.028 | 149.924 |  |
| LTL | TXNDC5 | 11212_7 | 89 | Mode Based | -0.017 | 0.057 | -0.129 | 0.094 | 0.761 | 0.028 | 149.924 |  |
| LTL | TXNDC5 | 11212_7 | 89 | MR-PRESSO (Raw) | 0.004 | 0.034 | -0.064 | 0.072 | 0.905 | 0.028 | 149.924 |  |
| LTL | TXNDC5 | 11212_7 | 89 | MR-PRESSO (Outlier-Corrected) | No Outliers Detected | | | | | | |  |
| LTL | MZB1 | 16322_10 | 89 | IVW | -0.083 | 0.048 | -0.177 | 0.010 | 0.080 | 0.028 | 149.924 |  |
| LTL | MZB1 | 16322_10 | 89 | Weighted median | -0.129 | 0.059 | -0.245 | -0.014 | 0.028 | 0.028 | 149.924 |  |
| LTL | MZB1 | 16322_10 | 89 | MR-Egger | -0.129 | 0.081 | -0.288 | 0.030 | 0.112 | 0.028 | 149.924 |  |
| LTL | MZB1 | 16322_10 | 89 | (intercept) | 0.002 | 0.003 | -0.003 | 0.007 | 0.487 | 0.028 | 149.924 |  |
| LTL | MZB1 | 16322_10 | 89 | Mode Based | -0.152 | 0.059 | -0.267 | -0.036 | 0.010 | 0.028 | 149.924 |  |
| LTL | MZB1 | 16322_10 | 89 | MR-PRESSO (Raw) | -0.083 | 0.048 | -0.178 | 0.011 | 0.083 | 0.028 | 149.924 |  |
| LTL | MZB1 | 16322_10 | 88 | MR-PRESSO (Outlier-Corrected) | -0.067 | 0.045 | -0.155 | 0.021 | 0.135 | 0.028 | 149.924 |  |
| LTL | TNFRSF17 | 2665_26 | 89 | IVW | -0.097 | 0.047 | -0.190 | -0.004 | 0.042 | 0.028 | 149.924 |  |
| LTL | TNFRSF17 | 2665_26 | 89 | Weighted median | -0.135 | 0.059 | -0.250 | -0.019 | 0.022 | 0.028 | 149.924 |  |
| LTL | TNFRSF17 | 2665_26 | 89 | MR-Egger | -0.146 | 0.081 | -0.304 | 0.012 | 0.069 | 0.028 | 149.924 |  |
| LTL | TNFRSF17 | 2665_26 | 89 | (intercept) | 0.002 | 0.002 | -0.003 | 0.007 | 0.445 | 0.028 | 149.924 |  |
| LTL | TNFRSF17 | 2665_26 | 89 | Mode Based | -0.204 | 0.059 | -0.320 | -0.089 | 0.001 | 0.028 | 149.924 |  |
| LTL | TNFRSF17 | 2665_26 | 89 | MR-PRESSO (Raw) | -0.097 | 0.047 | -0.191 | -0.003 | 0.044 | 0.028 | 149.924 |  |
| LTL | TNFRSF17 | 2665_26 | 87 | MR-PRESSO (Outlier-Corrected) | -0.103 | 0.042 | -0.186 | -0.019 | 0.016 | 0.028 | 149.924 |  |
| LTL | KDR | 3651_50 | 89 | IVW | 0.190 | 0.036 | 0.119 | 0.261 | 1.58E-07 | 0.028 | 149.924 |  |
| LTL | KDR | 3651_50 | 89 | Weighted median | 0.152 | 0.058 | 0.038 | 0.265 | 0.009 | 0.028 | 149.924 |  |
| LTL | KDR | 3651_50 | 89 | MR-Egger | 0.223 | 0.062 | 0.102 | 0.344 | 3.06E-04 | 0.028 | 149.924 |  |
| LTL | KDR | 3651_50 | 89 | (intercept) | -0.001 | 0.002 | -0.005 | 0.002 | 0.506 | 0.028 | 149.924 |  |
| LTL | KDR | 3651_50 | 89 | Mode Based | 0.148 | 0.062 | 0.025 | 0.270 | 0.018 | 0.028 | 149.924 |  |
| LTL | KDR | 3651_50 | 89 | MR-PRESSO (Raw) | 0.190 | 0.036 | 0.118 | 0.262 | 8.48E-07 | 0.028 | 149.924 |  |
| LTL | KDR | 3651_50 | 89 | MR-PRESSO (Outlier-Corrected) | No Outliers Detected | | | | | | |  |
| LTL | GDF15 | 4374_45 | 89 | IVW | -0.103 | 0.037 | -0.175 | -0.031 | 0.005 | 0.028 | 149.924 |  |
| LTL | GDF15 | 4374_45 | 89 | Weighted median | -0.170 | 0.056 | -0.279 | -0.061 | 0.002 | 0.028 | 149.924 |  |
| LTL | GDF15 | 4374_45 | 89 | MR-Egger | -0.178 | 0.062 | -0.300 | -0.056 | 0.004 | 0.028 | 149.924 |  |
| LTL | GDF15 | 4374_45 | 89 | (intercept) | 0.003 | 0.002 | -0.001 | 0.007 | 0.138 | 0.028 | 149.924 |  |
| LTL | GDF15 | 4374_45 | 89 | Mode Based | -0.202 | 0.057 | -0.313 | -0.090 | 3.84E-04 | 0.028 | 149.924 |  |
| LTL | GDF15 | 4374_45 | 89 | MR-PRESSO (Raw) | -0.103 | 0.037 | -0.176 | -0.030 | 0.006 | 0.028 | 149.924 |  |
| LTL | GDF15 | 4374_45 | 89 | MR-PRESSO (Outlier-Corrected) | No Outliers Detected | | | | | | |  |
| LTL | GP1Bα | 4990_87 | 89 | IVW | 0.101 | 0.047 | 0.008 | 0.194 | 0.033 | 0.028 | 149.924 |  |
| LTL | GP1Bα | 4990_87 | 89 | Weighted median | 0.128 | 0.062 | 0.007 | 0.249 | 0.038 | 0.028 | 149.924 |  |
| LTL | GP1Bα | 4990_87 | 89 | MR-Egger | 0.297 | 0.077 | 0.146 | 0.448 | 1.19E-04 | 0.028 | 149.924 |  |
| LTL | GP1Bα | 4990_87 | 89 | (intercept) | -0.008 | 0.002 | -0.012 | -0.003 | 0.002 | 0.028 | 149.924 |  |
| LTL | GP1Bα | 4990_87 | 89 | Mode Based | 0.056 | 0.085 | -0.111 | 0.223 | 0.510 | 0.028 | 149.924 |  |
| LTL | GP1Bα | 4990_87 | 89 | MR-PRESSO (Raw) | 0.101 | 0.047 | 0.007 | 0.195 | 0.035 | 0.028 | 149.924 |  |
| LTL | GP1Bα | 4990_87 | 86 | MR-PRESSO (Outlier-Corrected) | 0.073 | 0.042 | -0.010 | 0.155 | 0.084 | 0.028 | 149.924 |  |
| LTL | THPO | 5947_90 | 89 | IVW | -0.033 | 0.042 | -0.115 | 0.049 | 0.427 | 0.028 | 149.924 |  |
| LTL | THPO | 5947_90 | 89 | Weighted median | -0.003 | 0.058 | -0.117 | 0.111 | 0.955 | 0.028 | 149.924 |  |
| LTL | THPO | 5947_90 | 89 | MR-Egger | -0.059 | 0.070 | -0.197 | 0.079 | 0.403 | 0.028 | 149.924 |  |
| LTL | THPO | 5947_90 | 89 | (intercept) | 0.001 | 0.002 | -0.003 | 0.005 | 0.649 | 0.028 | 149.924 |  |
| LTL | THPO | 5947_90 | 89 | Mode Based | -0.014 | 0.072 | -0.155 | 0.127 | 0.845 | 0.028 | 149.924 |  |
| LTL | THPO | 5947_90 | 89 | MR-PRESSO (Raw) | -0.033 | 0.042 | -0.116 | 0.050 | 0.429 | 0.028 | 149.924 |  |
| LTL | THPO | 5947_90 | 87 | MR-PRESSO (Outlier-Corrected) | 0.006 | 0.036 | -0.064 | 0.077 | 0.865 | 0.028 | 149.924 |  |
| LTL | ST3GAL6 | 6947_4 | 89 | IVW | 0.145 | 0.030 | 0.085 | 0.205 | 1.90E-06 | 0.028 | 149.924 |  |
| LTL | ST3GAL6 | 6947_4 | 89 | Weighted median | 0.172 | 0.048 | 0.078 | 0.266 | 3.44E-04 | 0.028 | 149.924 |  |
| LTL | ST3GAL6 | 6947_4 | 89 | MR-Egger | 0.217 | 0.051 | 0.116 | 0.318 | 2.52E-05 | 0.028 | 149.924 |  |
| LTL | ST3GAL6 | 6947_4 | 89 | (intercept) | -0.003 | 0.002 | -0.006 | 0.000 | 0.085 | 0.028 | 149.924 |  |
| LTL | ST3GAL6 | 6947_4 | 89 | Mode Based | 0.184 | 0.051 | 0.083 | 0.284 | 3.45E-04 | 0.028 | 149.924 |  |
| LTL | ST3GAL6 | 6947_4 | 89 | MR-PRESSO (Raw) | 0.145 | 0.030 | 0.085 | 0.205 | 6.25E-06 | 0.028 | 149.924 |  |
| LTL | ST3GAL6 | 6947_4 | 89 | MR-PRESSO (Outlier-Corrected) | No Outliers Detected | | | | | | |  |
| LTL | CHL1 | 8958_51 | 89 | IVW | -0.252 | 0.037 | -0.324 | -0.180 | 5.80E-12 | 0.028 | 149.924 |  |
| LTL | CHL1 | 8958_51 | 89 | Weighted median | -0.225 | 0.054 | -0.330 | -0.119 | 2.85E-05 | 0.028 | 149.924 |  |
| LTL | CHL1 | 8958_51 | 89 | MR-Egger | -0.256 | 0.063 | -0.379 | -0.133 | 4.40E-05 | 0.028 | 149.924 |  |
| LTL | CHL1 | 8958_51 | 89 | (intercept) | 0.000 | 0.002 | -0.004 | 0.004 | 0.941 | 0.028 | 149.924 |  |
| LTL | CHL1 | 8958_51 | 89 | Mode Based | -0.217 | 0.059 | -0.333 | -0.102 | 2.14E-04 | 0.028 | 149.924 |  |
| LTL | CHL1 | 8958_51 | 89 | MR-PRESSO (Raw) | -0.252 | 0.037 | -0.325 | -0.180 | 4.68E-10 | 0.028 | 149.924 |  |
| LTL | CHL1 | 8958_51 | 89 | MR-PRESSO (Outlier-Corrected) | No Outliers Detected | | | | | | |  |
| * R² indicates variance explained by genetic instruments. ♰ F statistic represents the strength of the genetic instruments. IVW= inverse-variance weighted method; MR-PRESSO (Outlier-Corrected) test was presented after exclusion of outlier SNPs if outliers were detected. | | | | | | | | | | | |  |
|  |  |  |  |  |  |  |  |  |  |  |  |  |

| **Table S11. Forward Direction Mendelian Randomization (MR) Sensitivity Analysis Results with Added LD Proxies** | | | | | | | | | | | |
| --- | --- | --- | --- | --- | --- | --- | --- | --- | --- | --- | --- |
| Exposure | Outcome | Aptamer ID | NSNP | Method | Estimate | Std. Error | Lower 95% CI | Upper 95% CI | P-value | R2* | F♰ |
| LTL | PLOD3 | 10612_18 | 101 | IVW | -0.005 | 0.036 | -0.075 | 0.065 | 0.887 | 0.029 | 140.96 |
| LTL | PLOD3 | 10612_18 | 101 | Weighted median | -0.018 | 0.055 | -0.126 | 0.089 | 0.737 | 0.029 | 140.96 |
| LTL | PLOD3 | 10612_18 | 101 | MR-Egger | 0.027 | 0.061 | -0.093 | 0.147 | 0.66 | 0.029 | 140.96 |
| LTL | PLOD3 | 10612_18 | 101 | (intercept) | -0.001 | 0.002 | -0.005 | 0.002 | 0.519 | 0.029 | 140.96 |
| LTL | PLOD3 | 10612_18 | 101 | Mode Based | -0.048 | 0.057 | -0.16 | 0.063 | 0.4 | 0.029 | 140.96 |
| LTL | PLOD3 | 10612_18 | 101 | MR-PRESSO (Raw) | -0.005 | 0.036 | -0.075 | 0.065 | 0.887 | 0.029 | 140.96 |
| LTL | PLOD3 | 10612_18 | 101 | MR-PRESSO (Outlier-Corrected) | No Outliers Detected | | | | | | |
| LTL | COL28A1 | 10702_1 | 101 | IVW | -0.006 | 0.035 | -0.075 | 0.063 | 0.871 | 0.029 | 140.96 |
| LTL | COL28A1 | 10702_1 | 101 | Weighted median | -0.088 | 0.057 | -0.2 | 0.024 | 0.123 | 0.029 | 140.96 |
| LTL | COL28A1 | 10702_1 | 101 | MR-Egger | -0.004 | 0.06 | -0.123 | 0.114 | 0.944 | 0.029 | 140.96 |
| LTL | COL28A1 | 10702_1 | 101 | (intercept) | 0 | 0.002 | -0.004 | 0.004 | 0.976 | 0.029 | 140.96 |
| LTL | COL28A1 | 10702_1 | 101 | Mode Based | -0.081 | 0.054 | -0.187 | 0.025 | 0.137 | 0.029 | 140.96 |
| LTL | COL28A1 | 10702_1 | 101 | MR-PRESSO (Raw) | -0.006 | 0.035 | -0.075 | 0.063 | 0.871 | 0.029 | 140.96 |
| LTL | COL28A1 | 10702_1 | 101 | MR-PRESSO (Outlier-Corrected) | No Outliers Detected | | | | | | |
| LTL | SVEP1 | 11178_21 | 101 | IVW | -0.08 | 0.033 | -0.146 | -0.015 | 0.0162 | 0.029 | 140.96 |
| LTL | SVEP1 | 11178_21 | 101 | Weighted median | -0.111 | 0.053 | -0.214 | -0.008 | 0.0351 | 0.029 | 140.96 |
| LTL | SVEP1 | 11178_21 | 101 | MR-Egger | -0.108 | 0.058 | -0.221 | 0.005 | 0.0642 | 0.029 | 140.96 |
| LTL | SVEP1 | 11178_21 | 101 | (intercept) | 0.001 | 0.002 | -0.002 | 0.004 | 0.559 | 0.029 | 140.96 |
| LTL | SVEP1 | 11178_21 | 101 | Mode Based | -0.14 | 0.055 | -0.248 | -0.032 | 0.0127 | 0.029 | 140.96 |
| LTL | SVEP1 | 11178_21 | 101 | MR-PRESSO (Raw) | -0.08 | 0.03 | -0.138 | -0.022 | 0.00783 | 0.029 | 140.96 |
| LTL | SVEP1 | 11178_21 | 101 | MR-PRESSO (Outlier-Corrected) | No Outliers Detected | | | | | | |
| LTL | TXNDC5 | 11212_7 | 101 | IVW | -0.014 | 0.034 | -0.08 | 0.052 | 0.685 | 0.029 | 140.96 |
| LTL | TXNDC5 | 11212_7 | 101 | Weighted median | -0.034 | 0.055 | -0.142 | 0.075 | 0.543 | 0.029 | 140.96 |
| LTL | TXNDC5 | 11212_7 | 101 | MR-Egger | 0.071 | 0.058 | -0.042 | 0.184 | 0.218 | 0.029 | 140.96 |
| LTL | TXNDC5 | 11212_7 | 101 | (intercept) | -0.003 | 0.002 | -0.007 | 0 | 0.0719 | 0.029 | 140.96 |
| LTL | TXNDC5 | 11212_7 | 101 | Mode Based | -0.031 | 0.055 | -0.138 | 0.076 | 0.573 | 0.029 | 140.96 |
| LTL | TXNDC5 | 11212_7 | 101 | MR-PRESSO (Raw) | -0.014 | 0.033 | -0.078 | 0.051 | 0.679 | 0.029 | 140.96 |
| LTL | TXNDC5 | 11212_7 | 101 | MR-PRESSO (Outlier-Corrected) | No Outliers Detected | | | | | | |
| LTL | MZB1 | 16322_10 | 101 | IVW | -0.065 | 0.046 | -0.156 | 0.026 | 0.162 | 0.029 | 140.96 |
| LTL | MZB1 | 16322_10 | 101 | Weighted median | -0.128 | 0.058 | -0.242 | -0.013 | 0.0289 | 0.029 | 140.96 |
| LTL | MZB1 | 16322_10 | 101 | MR-Egger | -0.104 | 0.08 | -0.261 | 0.052 | 0.194 | 0.029 | 140.96 |
| LTL | MZB1 | 16322_10 | 101 | (intercept) | 0.001 | 0.002 | -0.003 | 0.006 | 0.544 | 0.029 | 140.96 |
| LTL | MZB1 | 16322_10 | 101 | Mode Based | -0.139 | 0.062 | -0.259 | -0.018 | 0.0267 | 0.029 | 140.96 |
| LTL | MZB1 | 16322_10 | 101 | MR-PRESSO (Raw) | -0.065 | 0.046 | -0.156 | 0.026 | 0.165 | 0.029 | 140.96 |
| LTL | MZB1 | 16322_10 | 100 | MR-PRESSO (Outlier-Corrected) | -0.055 | 0.044 | -0.142 | 0.032 | 0.218 | 0.029 | 140.96 |
| LTL | TNFRSF17 | 2665_26 | 101 | IVW | -0.108 | 0.047 | -0.2 | -0.016 | 0.0219 | 0.029 | 140.96 |
| LTL | TNFRSF17 | 2665_26 | 101 | Weighted median | -0.15 | 0.055 | -0.258 | -0.041 | 0.00699 | 0.029 | 140.96 |
| LTL | TNFRSF17 | 2665_26 | 101 | MR-Egger | -0.139 | 0.081 | -0.297 | 0.02 | 0.0892 | 0.029 | 140.96 |
| LTL | TNFRSF17 | 2665_26 | 101 | (intercept) | 0.001 | 0.002 | -0.004 | 0.006 | 0.638 | 0.029 | 140.96 |
| LTL | TNFRSF17 | 2665_26 | 101 | Mode Based | -0.215 | 0.056 | -0.324 | -0.106 | 0.000199 | 0.029 | 140.96 |
| LTL | TNFRSF17 | 2665_26 | 101 | MR-PRESSO (Raw) | -0.108 | 0.047 | -0.2 | -0.016 | 0.024 | 0.029 | 140.96 |
| LTL | TNFRSF17 | 2665_26 | 99 | MR-PRESSO (Outlier-Corrected) | -0.114 | 0.041 | -0.195 | -0.033 | 0.00671 | 0.029 | 140.96 |
| LTL | KDR | 3651_50 | 101 | IVW | 0.173 | 0.04 | 0.095 | 0.251 | 1.48E-05 | 0.029 | 140.96 |
| LTL | KDR | 3651_50 | 101 | Weighted median | 0.148 | 0.061 | 0.029 | 0.267 | 0.0149 | 0.029 | 140.96 |
| LTL | KDR | 3651_50 | 101 | MR-Egger | 0.224 | 0.068 | 0.09 | 0.358 | 0.00147 | 0.029 | 140.96 |
| LTL | KDR | 3651_50 | 101 | (intercept) | -0.002 | 0.002 | -0.006 | 0.002 | 0.36 | 0.029 | 140.96 |
| LTL | KDR | 3651_50 | 101 | Mode Based | 0.146 | 0.062 | 0.025 | 0.268 | 0.0201 | 0.029 | 140.96 |
| LTL | KDR | 3651_50 | 101 | MR-PRESSO (Raw) | 0.173 | 0.04 | 0.095 | 0.251 | 3.52E-05 | 0.029 | 140.96 |
| LTL | KDR | 3651_50 | 101 | MR-PRESSO (Outlier-Corrected) | No Outliers Detected | | | | | | |
| LTL | GDF15 | 4374_45 | 101 | IVW | -0.116 | 0.037 | -0.189 | -0.044 | 0.00157 | 0.029 | 140.96 |
| LTL | GDF15 | 4374_45 | 101 | Weighted median | -0.182 | 0.053 | -0.286 | -0.078 | 0.00062 | 0.029 | 140.96 |
| LTL | GDF15 | 4374_45 | 101 | MR-Egger | -0.163 | 0.063 | -0.287 | -0.039 | 0.0113 | 0.029 | 140.96 |
| LTL | GDF15 | 4374_45 | 101 | (intercept) | 0.002 | 0.002 | -0.002 | 0.006 | 0.367 | 0.029 | 140.96 |
| LTL | GDF15 | 4374_45 | 101 | Mode Based | -0.21 | 0.058 | -0.324 | -0.095 | 0.000509 | 0.029 | 140.96 |
| LTL | GDF15 | 4374_45 | 101 | MR-PRESSO (Raw) | -0.116 | 0.037 | -0.189 | -0.044 | 0.00208 | 0.029 | 140.96 |
| LTL | GDF15 | 4374_45 | 101 | MR-PRESSO (Outlier-Corrected) | No Outliers Detected | | | | | | |
| LTL | GP1Bα | 4990_87 | 101 | IVW | 0.119 | 0.047 | 0.027 | 0.211 | 0.0111 | 0.029 | 140.96 |
| LTL | GP1Bα | 4990_87 | 101 | Weighted median | 0.157 | 0.063 | 0.033 | 0.281 | 0.0132 | 0.029 | 140.96 |
| LTL | GP1Bα | 4990_87 | 101 | MR-Egger | 0.298 | 0.078 | 0.146 | 0.45 | 0.000215 | 0.029 | 140.96 |
| LTL | GP1Bα | 4990_87 | 101 | (intercept) | -0.007 | 0.002 | -0.011 | -0.002 | 0.00542 | 0.029 | 140.96 |
| LTL | GP1Bα | 4990_87 | 101 | Mode Based | 0.074 | 0.093 | -0.108 | 0.257 | 0.427 | 0.029 | 140.96 |
| LTL | GP1Bα | 4990_87 | 101 | MR-PRESSO (Raw) | 0.119 | 0.047 | 0.027 | 0.211 | 0.0127 | 0.029 | 140.96 |
| LTL | GP1Bα | 4990_87 | 99 | MR-PRESSO (Outlier-Corrected) | 0.094 | 0.044 | 0.008 | 0.179 | 0.0342 | 0.029 | 140.96 |
| LTL | THPO | 5947_90 | 101 | IVW | -0.05 | 0.04 | -0.129 | 0.03 | 0.22 | 0.029 | 140.96 |
| LTL | THPO | 5947_90 | 101 | Weighted median | -0.005 | 0.059 | -0.12 | 0.111 | 0.939 | 0.029 | 140.96 |
| LTL | THPO | 5947_90 | 101 | MR-Egger | -0.061 | 0.069 | -0.197 | 0.074 | 0.376 | 0.029 | 140.96 |
| LTL | THPO | 5947_90 | 101 | (intercept) | 0 | 0.002 | -0.004 | 0.005 | 0.833 | 0.029 | 140.96 |
| LTL | THPO | 5947_90 | 101 | Mode Based | -0.015 | 0.073 | -0.158 | 0.128 | 0.836 | 0.029 | 140.96 |
| LTL | THPO | 5947_90 | 101 | MR-PRESSO (Raw) | -0.05 | 0.04 | -0.129 | 0.03 | 0.223 | 0.029 | 140.96 |
| LTL | THPO | 5947_90 | 98 | MR-PRESSO (Outlier-Corrected) | -0.002 | 0.037 | -0.073 | 0.07 | 0.961 | 0.029 | 140.96 |
| LTL | ST3GAL6 | 6947_4 | 101 | IVW | 0.152 | 0.031 | 0.092 | 0.213 | 8.67E-07 | 0.029 | 140.96 |
| LTL | ST3GAL6 | 6947_4 | 101 | Weighted median | 0.209 | 0.048 | 0.114 | 0.303 | 1.55E-05 | 0.029 | 140.96 |
| LTL | ST3GAL6 | 6947_4 | 101 | MR-Egger | 0.234 | 0.053 | 0.13 | 0.337 | 2.39E-05 | 0.029 | 140.96 |
| LTL | ST3GAL6 | 6947_4 | 101 | (intercept) | -0.003 | 0.002 | -0.006 | 0 | 0.0609 | 0.029 | 140.96 |
| LTL | ST3GAL6 | 6947_4 | 101 | Mode Based | 0.186 | 0.049 | 0.091 | 0.282 | 0.000226 | 0.029 | 140.96 |
| LTL | ST3GAL6 | 6947_4 | 101 | MR-PRESSO (Raw) | 0.152 | 0.031 | 0.092 | 0.213 | 3.42E-06 | 0.029 | 140.96 |
| LTL | ST3GAL6 | 6947_4 | 101 | MR-PRESSO (Outlier-Corrected) | No Outliers Detected | | | | | | |
| LTL | CHL1 | 8958_51 | 101 | IVW | -0.248 | 0.037 | -0.32 | -0.176 | 1.81E-11 | 0.029 | 140.96 |
| LTL | CHL1 | 8958_51 | 101 | Weighted median | -0.224 | 0.054 | -0.33 | -0.119 | 2.92E-05 | 0.029 | 140.96 |
| LTL | CHL1 | 8958_51 | 101 | MR-Egger | -0.233 | 0.064 | -0.357 | -0.108 | 0.000417 | 0.029 | 140.96 |
| LTL | CHL1 | 8958_51 | 101 | (intercept) | -0.001 | 0.002 | -0.004 | 0.003 | 0.766 | 0.029 | 140.96 |
| LTL | CHL1 | 8958_51 | 101 | Mode Based | -0.216 | 0.061 | -0.336 | -0.096 | 0.000619 | 0.029 | 140.96 |
| LTL | CHL1 | 8958_51 | 101 | MR-PRESSO (Raw) | -0.248 | 0.037 | -0.32 | -0.176 | 1.13E-09 | 0.029 | 140.96 |
| LTL | CHL1 | 8958_51 | 101 | MR-PRESSO (Outlier-Corrected) | No Outliers Detected | | | | | | |
| Sensitivity analyses with additional IVs added. Missing Codd et al. MR variants were replaced with LD proxies (European ancestry reference; prioritized r² ≥ 0.8 with a minimum r² ≥ 0.5; proxies listed in Supplementary Table S13). R² indicates the variance explained by the genetic instruments, and the F statistic represents instrument strength. IVW denotes the inverse-variance weighted method. MR-PRESSO (outlier-corrected) results are reported after exclusion of detected outlier SNPs, where applicable. | | | | | | | | | | | |

| **Table S12. Summary of Instrumental Variables Used in the LTL-Protein Forward Direction MR Analysis** | | | | | | | |
| --- | --- | --- | --- | --- | --- | --- | --- |
| **SNP** | **Effect Allele** | **Other Allele** | **Chr** | **Position (GRCh37)** | **Position (GRCh38)** | **R2*** | **F♰** |
| rs66731853 | G | A | 1 | 20916238 | 20589745 | 1.44E-04 | 68.115 |
| rs17185038 | C | G | 1 | 28219658 | 27893147 | 8.55E-05 | 40.356 |
| rs6669563 | G | A | 1 | 32279629 | 31814028 | 1.72E-04 | 81.115 |
| rs41269079 | T | A | 1 | 45252015 | 44786343 | 7.69E-05 | 36.294 |
| rs139795227 | A | C | 1 | 92842367 | 92376810 | 9.98E-05 | 47.112 |
| rs4498805 | G | T | 1 | 110910397 | 110367775 | 1.20E-04 | 56.489 |
| rs11579626 | A | C | 1 | 146741960 | 147270300 | 1.16E-04 | 54.916 |
| rs61818036 | G | A | 1 | 151364199 | 151391723 | 1.05E-04 | 49.379 |
| rs932002 | C | T | 1 | 226577306 | 226389605 | 4.38E-04 | 206.671 |
| rs56178008 | T | A | 2 | 29098543 | 28875677 | 1.08E-04 | 50.904 |
| rs12613375 | C | T | 2 | 58984109 | 58756974 | 7.94E-05 | 37.501 |
| rs35671754 | G | T | 2 | 216220870 | 215356147 | 6.82E-05 | 32.185 |
| rs869785 | T | C | 3 | 24347800 | 24306309 | 1.01E-04 | 47.915 |
| rs575032615 | A | G | 3 | 47638657 | 47597150 | 1.07E-04 | 50.745 |
| rs78491606 | A | C | 3 | 72891547 | 72842396 | 2.20E-04 | 104.128 |
| rs6776756 | G | A | 3 | 128215821 | 128496978 | 1.55E-04 | 73.300 |
| rs41272947 | G | A | 3 | 160119525 | 160401737 | 1.58E-04 | 74.541 |
| rs2293607 | T | C | 3 | 169482335 | 169764547 | 3.46E-03 | 1638.272 |
| rs871134 | C | T | 4 | 7044380 | 7042653 | 1.73E-04 | 81.550 |
| rs13129697 | T | G | 4 | 9926967 | 9925343 | 1.25E-04 | 59.113 |
| rs4435700 | C | A | 4 | 164020174 | 163099022 | 1.07E-03 | 507.527 |
| rs7705526 | C | A | 5 | 1285974 | 1285859 | 2.72E-03 | 1289.259 |
| rs61748181 | C | T | 5 | 1294166 | 1294051 | 2.09E-04 | 98.799 |
| rs80324517 | G | A | 6 | 204031 | 204031 | 1.53E-04 | 72.312 |
| rs13230646 | T | C | 7 | 23930316 | 23890697 | 1.18E-04 | 55.602 |
| rs11769630 | T | A | 7 | 50257703 | 50218107 | 9.21E-05 | 43.476 |
| rs2056726 | G | A | 7 | 99780283 | 100182660 | 1.86E-04 | 87.634 |
| rs609953 | T | A | 7 | 123422444 | 123782390 | 9.00E-05 | 42.513 |
| rs7790856 | C | T | 7 | 124459852 | 124819798 | 8.32E-04 | 393.040 |
| rs4731541 | C | G | 7 | 128678236 | 129038182 | 2.12E-04 | 100.152 |
| rs1985369 | A | G | 7 | 159119220 | 159326530 | 2.27E-04 | 107.403 |
| rs2306646 | G | C | 8 | 21846586 | 21989075 | 2.28E-04 | 107.587 |
| rs762679 | T | A | 8 | 48885436 | 47972876 | 2.51E-04 | 118.372 |
| rs7012816 | G | A | 8 | 70964743 | 70052508 | 7.45E-05 | 35.165 |
| rs10112752 | G | A | 8 | 73958718 | 73046483 | 4.27E-04 | 201.564 |
| rs1023767 | G | A | 8 | 95530969 | 94518741 | 1.30E-04 | 61.246 |
| rs4743037 | C | T | 9 | 109639970 | 106877689 | 8.18E-05 | 38.624 |
| rs12572897 | G | A | 10 | 96114835 | 94355078 | 2.47E-04 | 116.572 |
| rs4919611 | C | A | 10 | 103894939 | 102135182 | 1.39E-04 | 65.750 |
| rs9419958 | T | C | 10 | 105675946 | 103916188 | 1.61E-03 | 760.030 |
| rs939916 | G | A | 11 | 202253 | 202253 | 2.64E-04 | 124.474 |
| rs10840270 | C | G | 11 | 9629553 | 9608006 | 9.70E-05 | 45.815 |
| rs611646 | T | A | 11 | 108177097 | 108306370 | 6.93E-04 | 327.411 |
| rs6590343 | A | G | 11 | 128500215 | 128630320 | 7.73E-05 | 36.522 |
| rs10845387 | G | A | 12 | 11757743 | 11604809 | 9.63E-05 | 45.480 |
| rs12369950 | T | C | 12 | 24762109 | 24609175 | 7.99E-05 | 37.751 |
| rs1907702 | G | A | 12 | 88955469 | 88561692 | 8.12E-05 | 38.339 |
| rs76666449 | T | C | 12 | 120904895 | 120467092 | 1.66E-04 | 78.458 |
| rs4758644 | A | C | 12 | 122943915 | 122459368 | 1.13E-04 | 53.403 |
| rs1332941 | A | G | 13 | 41695100 | 41120964 | 1.87E-04 | 88.210 |
| rs3093888 | G | A | 14 | 20812951 | 20344792 | 8.68E-05 | 41.004 |
| rs73581419 | C | T | 14 | 21941148 | 21472989 | 1.06E-04 | 50.273 |
| rs12884911 | C | T | 14 | 65027871 | 64561153 | 9.36E-05 | 44.217 |
| rs762810 | C | A | 14 | 65544367 | 65077649 | 1.98E-04 | 93.399 |
| rs137901416 | G | A | 14 | 73418095 | 72951387 | 4.01E-04 | 189.237 |
| rs1957937 | A | T | 14 | 96181360 | 95715023 | 1.24E-04 | 58.659 |
| rs17677991 | C | G | 15 | 42032383 | 41740185 | 2.36E-04 | 111.566 |
| rs181647350 | T | C | 15 | 50379219 | 50087018 | 4.28E-04 | 201.990 |
| rs1980240 | A | C | 15 | 56774018 | 56481820 | 8.54E-05 | 40.312 |
| rs11646283 | T | C | 16 | 9073060 | 8979203 | 1.20E-04 | 56.614 |
| rs182059586 | T | C | 16 | 14652220 | 14558363 | 1.49E-04 | 70.373 |
| rs76219171 | G | A | 16 | 50188929 | 50155018 | 1.47E-04 | 69.465 |
| rs62053340 | C | T | 16 | 69987764 | 69953861 | 2.22E-04 | 104.969 |
| rs34003787 | C | T | 16 | 73071381 | 73037482 | 9.54E-05 | 45.048 |
| rs11866592 | G | A | 16 | 74654396 | 74620498 | 3.09E-04 | 146.072 |
| rs2303262 | C | T | 16 | 82203758 | 82170153 | 8.10E-04 | 382.930 |
| rs62046862 | C | A | 16 | 88073029 | 88039423 | 2.84E-04 | 134.223 |
| rs9923119 | T | C | 16 | 90153815 | 90087407 | 1.15E-04 | 54.214 |
| rs7218033 | C | T | 17 | 1694247 | 1790953 | 2.04E-04 | 96.265 |
| rs4724 | G | A | 17 | 7760397 | 7857079 | 6.50E-04 | 307.004 |
| rs75664430 | C | G | 17 | 8064779 | 8161461 | 2.18E-04 | 102.883 |
| rs111527438 | T | C | 17 | 29252703 | 30925685 | 7.43E-05 | 35.090 |
| rs12941945 | A | G | 17 | 41448228 | 43370860 | 1.99E-04 | 94.099 |
| rs2069536 | A | G | 17 | 74001106 | 76005025 | 8.61E-05 | 40.679 |
| rs144204502 | C | T | 17 | 76183233 | 78187152 | 2.57E-04 | 121.249 |
| rs3891167 | A | G | 18 | 658423 | 658423 | 6.68E-04 | 315.776 |
| rs8088824 | C | T | 18 | 42151261 | 44571296 | 2.53E-04 | 119.498 |
| rs2276182 | C | G | 18 | 51798047 | 54271677 | 2.77E-04 | 130.728 |
| rs6565924 | A | G | 18 | 74691225 | 76979268 | 8.41E-05 | 39.711 |
| rs1879100 | C | T | 18 | 77985740 | 80227857 | 8.94E-05 | 42.234 |
| rs35601737 | C | G | 19 | 13220703 | 13109889 | 8.59E-05 | 40.547 |
| rs8105767 | A | G | 19 | 22215441 | 22032639 | 4.71E-04 | 222.564 |
| rs4530278 | G | T | 19 | 33752994 | 33262088 | 9.64E-05 | 45.540 |
| rs8102497 | G | A | 19 | 57370055 | 56858687 | 1.16E-04 | 54.708 |
| rs1291143 | A | C | 20 | 35525640 | 36897237 | 6.57E-04 | 310.392 |
| rs35640778 | G | A | 20 | 62321128 | 63689775 | 1.87E-03 | 886.247 |
| rs115610405 | C | A | 20 | 62325833 | 63694480 | 4.72E-04 | 223.196 |
| rs111527478 | G | A | 20 | 62678100 | 64046747 | 8.79E-05 | 41.490 |
| rs28502153 | C | A | 22 | 17469049 | 16988159 | 2.32E-04 | 109.637 |
| SNPs used as genetic instruments for the primary forward MR analysis (LTL → protein). Listed are effect and other alleles, chromosome, base-pair positions (GRCh37/GRCh38), R² (variance in LTL explained by each SNP), and F statistics (instrument strength). Abbreviations: SNP, single nucleotide polymorphism; R², variance explained; F, F statistic. | | | | | | | |

| **Table S13. LD Proxy Variants Used in Sensitivity Forward MR Analysis** | | | | | | | |
| --- | --- | --- | --- | --- | --- | --- | --- |
| **SNP** | **Effect Allele** | **Other Allele** | **Chr** | **Position (GRCh37)** | **Proxy For** | **R2*** |  |
| rs10167027 | C | G | 2 | 17874177 | rs9752694 | 1 |  |
| rs1558438 | TC | T | 2 | 210667432 | rs775145631 | 0.9918 |  |
| rs10026945 | CAAAAA | C | 4 | 2191750 | rs753936006 | 0.9769 |  |
| rs10023020 | CACTT | C | 4 | 122729413 | rs35500378 | 1 |  |
| rs3852312 | T | C | 7 | 76310784 | rs2538745 | 0.5053 |  |
| rs2890365 | CAAACAT | C | 10 | 5816070 | rs762222726 | 0.9873 |  |
| rs117712971 | G | A | 13 | 73340177 | rs35017269 | 1 |  |
| rs8048108 | C | A | 16 | 50089038 | rs12447324 | 0.9258 |  |
| rs78046419 | C | T | 16 | 67694044 | rs142507451 | 1 |  |
| rs183556764 | G | A | 18 | 710980 | rs78694226 | 1 |  |
| rs147068659 | G | T | 19 | 4105089 | rs80337039 | 0.733 |  |
| rs181080831 | A | C | 20 | 62358869 | rs187577818 | 1 |  |
| LD proxy variants added as additional IVs in the forward MR sensitivity analysis. “Proxy for” indicates the original LTL instrument variant that was unavailable in the deCODE protein GWAS. R² denotes the LD (r²) between each proxy variant and its corresponding original instrument in a European ancestry reference panel. | | | | | | | |

|  | **Table S14. Backward Direction Mendelian Randomization (MR) Analysis Results** | | | | | | | | |  |  |  |
| --- | --- | --- | --- | --- | --- | --- | --- | --- | --- | --- | --- | --- |
| **Exposure** | **Aptamer ID** | **Outcome** | **NSNP** | **Method** | **Estimate** | **Std. Error** | **Lower 95% CI** | **Upper 95% CI** | **P-value** | **R2*** | **F♰** |  |
| PLOD3 | 10612_18 | LTL | 30 | IVW | -0.0004 | 0.004 | -0.009 | 0.008 | 0.936 | 0.0036 | 56.687 |  |
| PLOD3 | 10612_18 | LTL | 30 | Weighted median | -0.0041 | 0.006 | -0.016 | 0.008 | 0.498 | 0.0036 | 56.687 |  |
| PLOD3 | 10612_18 | LTL | 30 | MR-Egger | -0.0021 | 0.007 | -0.015 | 0.011 | 0.746 | 0.0036 | 56.687 |  |
| PLOD3 | 10612_18 | LTL | 30 | (intercept) | 0.0005 | 0.001 | -0.002 | 0.003 | 0.716 | 0.0036 | 56.687 |  |
| PLOD3 | 10612_18 | LTL | 30 | Mode Based | -0.0042 | 0.006 | -0.016 | 0.007 | 0.468 | 0.0036 | 56.687 |  |
| PLOD3 | 10612_18 | LTL | 30 | MR-PRESSO (Raw) | -0.0004 | 0.004 | -0.008 | 0.007 | 0.924 | 0.0036 | 56.687 |  |
| PLOD3 | 10612_18 | LTL | 30 | MR-PRESSO (Outlier-Corrected) | No Outliers Detected | | | | | | |  |
| COL28A1 | 10702_1 | LTL | 9 | IVW | -0.0133 | 0.023 | -0.057 | 0.031 | 0.557 | 0.0007 | 37.819 |  |
| COL28A1 | 10702_1 | LTL | 9 | Weighted median | 0.0001 | 0.020 | -0.039 | 0.039 | 0.995 | 0.0007 | 37.819 |  |
| COL28A1 | 10702_1 | LTL | 9 | MR-Egger | 0.0313 | 0.077 | -0.119 | 0.182 | 0.684 | 0.0007 | 37.819 |  |
| COL28A1 | 10702_1 | LTL | 9 | (intercept) | -0.0029 | 0.005 | -0.012 | 0.006 | 0.542 | 0.0007 | 37.819 |  |
| COL28A1 | 10702_1 | LTL | 9 | Mode Based | 0.0062 | 0.025 | -0.042 | 0.054 | 0.802 | 0.0007 | 37.819 |  |
| COL28A1 | 10702_1 | LTL | 9 | MR-PRESSO (Raw) | -0.0133 | 0.023 | -0.067 | 0.040 | 0.573 | 0.0007 | 37.819 |  |
| COL28A1 | 10702_1 | LTL | 8 | MR-PRESSO (Outlier-Corrected) | -0.0012 | 0.019 | -0.048 | 0.046 | 0.951 | 0.0007 | 37.819 |  |
| SVEP1 | 11178_21 | LTL | 61 | IVW | -0.0022 | 0.004 | -0.009 | 0.005 | 0.543 | 0.0161 | 124.532 |  |
| SVEP1 | 11178_21 | LTL | 61 | Weighted median | 0.0049 | 0.005 | -0.004 | 0.014 | 0.283 | 0.0161 | 124.532 |  |
| SVEP1 | 11178_21 | LTL | 61 | MR-Egger | -0.0002 | 0.006 | -0.012 | 0.011 | 0.978 | 0.0161 | 124.532 |  |
| SVEP1 | 11178_21 | LTL | 61 | (intercept) | -0.0004 | 0.001 | -0.002 | 0.002 | 0.659 | 0.0161 | 124.532 |  |
| SVEP1 | 11178_21 | LTL | 61 | Mode Based | 0.0034 | 0.005 | -0.006 | 0.013 | 0.476 | 0.0161 | 124.532 |  |
| SVEP1 | 11178_21 | LTL | 61 | MR-PRESSO (Raw) | -0.0022 | 0.004 | -0.009 | 0.005 | 0.545 | 0.0161 | 124.532 |  |
| SVEP1 | 11178_21 | LTL | 60 | MR-PRESSO (Outlier-Corrected) | -0.0014 | 0.003 | -0.008 | 0.005 | 0.671 | 0.0161 | 124.532 |  |
| TXNDC5 | 11212_7 | LTL | 31 | IVW | -0.0019 | 0.003 | -0.007 | 0.004 | 0.496 | 0.0067 | 101.565 |  |
| TXNDC5 | 11212_7 | LTL | 31 | Weighted median | -0.0011 | 0.004 | -0.009 | 0.007 | 0.789 | 0.0067 | 101.565 |  |
| TXNDC5 | 11212_7 | LTL | 31 | MR-Egger | -0.0023 | 0.004 | -0.011 | 0.006 | 0.574 | 0.0067 | 101.565 |  |
| TXNDC5 | 11212_7 | LTL | 31 | (intercept) | 0.0002 | 0.001 | -0.002 | 0.002 | 0.889 | 0.0067 | 101.565 |  |
| TXNDC5 | 11212_7 | LTL | 31 | Mode Based | -0.0008 | 0.003 | -0.007 | 0.006 | 0.801 | 0.0067 | 101.565 |  |
| TXNDC5 | 11212_7 | LTL | 31 | MR-PRESSO (Raw) | -0.0019 | 0.003 | -0.008 | 0.004 | 0.492 | 0.0067 | 101.565 |  |
| TXNDC5 | 11212_7 | LTL | 31 | MR-PRESSO (Outlier-Corrected) | No Outliers Detected | | | | | | |  |
| MZB1 | 16322_10 | LTL | 32 | IVW | 0.0113 | 0.008 | -0.005 | 0.028 | 0.175 | 0.0048 | 70.435 |  |
| MZB1 | 16322_10 | LTL | 32 | Weighted median | 0.0122 | 0.007 | -0.002 | 0.026 | 0.092 | 0.0048 | 70.435 |  |
| MZB1 | 16322_10 | LTL | 32 | MR-Egger | 0.0066 | 0.015 | -0.023 | 0.036 | 0.660 | 0.0048 | 70.435 |  |
| MZB1 | 16322_10 | LTL | 32 | (intercept) | 0.0006 | 0.002 | -0.002 | 0.004 | 0.714 | 0.0048 | 70.435 |  |
| MZB1 | 16322_10 | LTL | 32 | Mode Based | 0.0107 | 0.008 | -0.004 | 0.026 | 0.162 | 0.0048 | 70.435 |  |
| MZB1 | 16322_10 | LTL | 32 | MR-PRESSO (Raw) | 0.0113 | 0.008 | -0.006 | 0.028 | 0.184 | 0.0048 | 70.435 |  |
| MZB1 | 16322_10 | LTL | 29 | MR-PRESSO (Outlier-Corrected) | 0.0141 | 0.006 | 0.002 | 0.026 | 0.023 | 0.0048 | 70.435 |  |
| TNFRSF17 | 2665_26 | LTL | 24 | IVW | -0.0017 | 0.019 | -0.038 | 0.035 | 0.928 | 0.0030 | 58.233 |  |
| TNFRSF17 | 2665_26 | LTL | 24 | Weighted median | -0.0027 | 0.011 | -0.024 | 0.019 | 0.808 | 0.0030 | 58.233 |  |
| TNFRSF17 | 2665_26 | LTL | 24 | MR-Egger | 0.0223 | 0.047 | -0.070 | 0.115 | 0.637 | 0.0030 | 58.233 |  |
| TNFRSF17 | 2665_26 | LTL | 24 | (intercept) | -0.0021 | 0.004 | -0.010 | 0.005 | 0.579 | 0.0030 | 58.233 |  |
| TNFRSF17 | 2665_26 | LTL | 24 | Mode Based | -0.0010 | 0.015 | -0.030 | 0.028 | 0.945 | 0.0030 | 58.233 |  |
| TNFRSF17 | 2665_26 | LTL | 24 | MR-PRESSO (Raw) | -0.0017 | 0.019 | -0.041 | 0.037 | 0.929 | 0.0030 | 58.233 |  |
| TNFRSF17 | 2665_26 | LTL | 21 | MR-PRESSO (Outlier-Corrected) | -0.0035 | 0.009 | -0.022 | 0.015 | 0.695 | 0.0030 | 58.233 |  |
| KDR | 3651_50 | LTL | 48 | IVW | -0.0030 | 0.004 | -0.011 | 0.005 | 0.487 | 0.0117 | 115.442 |  |
| KDR | 3651_50 | LTL | 48 | Weighted median | -0.0050 | 0.005 | -0.015 | 0.005 | 0.318 | 0.0117 | 115.442 |  |
| KDR | 3651_50 | LTL | 48 | MR-Egger | -0.0049 | 0.007 | -0.019 | 0.009 | 0.476 | 0.0117 | 115.442 |  |
| KDR | 3651_50 | LTL | 48 | (intercept) | 0.0004 | 0.001 | -0.002 | 0.003 | 0.717 | 0.0117 | 115.442 |  |
| KDR | 3651_50 | LTL | 48 | Mode Based | -0.0024 | 0.005 | -0.012 | 0.007 | 0.607 | 0.0117 | 115.442 |  |
| KDR | 3651_50 | LTL | 48 | MR-PRESSO (Raw) | -0.0030 | 0.004 | -0.012 | 0.006 | 0.491 | 0.0117 | 115.442 |  |
| KDR | 3651_50 | LTL | 47 | MR-PRESSO (Outlier-Corrected) | -0.0046 | 0.003 | -0.011 | 0.002 | 0.174 | 0.0117 | 115.442 |  |
| GDF15 | 4374_45 | LTL | 43 | IVW | -0.0087 | 0.004 | -0.017 | -0.001 | 0.030 | 0.0094 | 103.678 |  |
| GDF15 | 4374_45 | LTL | 43 | Weighted median | -0.0145 | 0.006 | -0.026 | -0.003 | 0.013 | 0.0094 | 103.678 |  |
| GDF15 | 4374_45 | LTL | 43 | MR-Egger | -0.0201 | 0.007 | -0.033 | -0.007 | 0.003 | 0.0094 | 103.678 |  |
| GDF15 | 4374_45 | LTL | 43 | (intercept) | 0.0022 | 0.001 | 0.000 | 0.004 | 0.037 | 0.0094 | 103.678 |  |
| GDF15 | 4374_45 | LTL | 43 | Mode Based | -0.0145 | 0.005 | -0.025 | -0.004 | 0.008 | 0.0094 | 103.678 |  |
| GDF15 | 4374_45 | LTL | 43 | MR-PRESSO (Raw) | -0.0087 | 0.004 | -0.017 | -0.001 | 0.036 | 0.0094 | 103.678 |  |
| GDF15 | 4374_45 | LTL | 43 | MR-PRESSO (Outlier-Corrected) | No Outliers Detected | | | | | | |  |
| GP1Bα | 4990_87 | LTL | 29 | IVW | -0.0221 | 0.009 | -0.041 | -0.004 | 0.018 | 0.0046 | 75.552 |  |
| GP1Bα | 4990_87 | LTL | 29 | Weighted median | -0.0182 | 0.009 | -0.035 | -0.001 | 0.038 | 0.0046 | 75.552 |  |
| GP1Bα | 4990_87 | LTL | 29 | MR-Egger | -0.0598 | 0.017 | -0.093 | -0.026 | 0.000 | 0.0046 | 75.552 |  |
| GP1Bα | 4990_87 | LTL | 29 | (intercept) | 0.0042 | 0.002 | 0.001 | 0.007 | 0.011 | 0.0046 | 75.552 |  |
| GP1Bα | 4990_87 | LTL | 29 | Mode Based | -0.0205 | 0.012 | -0.045 | 0.004 | 0.097 | 0.0046 | 75.552 |  |
| GP1Bα | 4990_87 | LTL | 29 | MR-PRESSO (Raw) | -0.0221 | 0.009 | -0.041 | -0.003 | 0.025 | 0.0046 | 75.552 |  |
| GP1Bα | 4990_87 | LTL | 27 | MR-PRESSO (Outlier-Corrected) | -0.0110 | 0.007 | -0.025 | 0.003 | 0.123 | 0.0046 | 75.552 |  |
| THPO | 5947_90 | LTL | 6 | IVW | -0.0669 | 0.072 | -0.208 | 0.074 | 0.352 | 0.0005 | 37.344 |  |
| THPO | 5947_90 | LTL | 6 | Weighted median | -0.0197 | 0.023 | -0.065 | 0.026 | 0.396 | 0.0005 | 37.344 |  |
| THPO | 5947_90 | LTL | 6 | MR-Egger | -0.0537 | 0.215 | -0.475 | 0.368 | 0.803 | 0.0005 | 37.344 |  |
| THPO | 5947_90 | LTL | 6 | (intercept) | -0.0010 | 0.015 | -0.030 | 0.028 | 0.947 | 0.0005 | 37.344 |  |
| THPO | 5947_90 | LTL | 6 | Mode Based | 0.0026 | 0.024 | -0.044 | 0.049 | 0.912 | 0.0005 | 37.344 |  |
| THPO | 5947_90 | LTL | 6 | MR-PRESSO (Raw) | -0.0669 | 0.072 | -0.266 | 0.133 | 0.395 | 0.0005 | 37.344 |  |
| THPO | 5947_90 | LTL | 3 | MR-PRESSO (Outlier-Corrected) | -0.0422 | 0.028 | -0.401 | 0.317 | 0.273 | 0.0005 | 37.344 |  |
| ST3GAL6 | 6947_4 | LTL | 210 | IVW | -0.0019 | 0.001 | -0.004 | 0.000 | 0.089 | 0.0602 | 135.868 |  |
| ST3GAL6 | 6947_4 | LTL | 210 | Weighted median | -0.0011 | 0.001 | -0.004 | 0.002 | 0.459 | 0.0602 | 135.868 |  |
| ST3GAL6 | 6947_4 | LTL | 210 | MR-Egger | -0.0044 | 0.001 | -0.007 | -0.002 | 0.001 | 0.0602 | 135.868 |  |
| ST3GAL6 | 6947_4 | LTL | 210 | (intercept) | 0.0013 | 0.000 | 0.000 | 0.002 | 0.002 | 0.0602 | 135.868 |  |
| ST3GAL6 | 6947_4 | LTL | 210 | Mode Based | -0.0029 | 0.001 | -0.005 | 0.000 | 0.019 | 0.0602 | 135.868 |  |
| ST3GAL6 | 6947_4 | LTL | 210 | MR-PRESSO (Raw) | -0.0019 | 0.001 | -0.004 | 0.000 | 0.090 | 0.0602 | 135.868 |  |
| ST3GAL6 | 6947_4 | LTL | 209 | MR-PRESSO (Outlier-Corrected) | -0.0020 | 0.001 | -0.004 | 0.000 | 0.050 | 0.0602 | 135.868 |  |
| CHL1 | 8958_51 | LTL | 27 | IVW | -0.0058 | 0.010 | -0.026 | 0.014 | 0.569 | 0.0056 | 98.682 |  |
| CHL1 | 8958_51 | LTL | 27 | Weighted median | 0.0026 | 0.005 | -0.008 | 0.013 | 0.614 | 0.0056 | 98.682 |  |
| CHL1 | 8958_51 | LTL | 27 | MR-Egger | 0.0229 | 0.014 | -0.006 | 0.051 | 0.114 | 0.0056 | 98.682 |  |
| CHL1 | 8958_51 | LTL | 27 | (intercept) | -0.0065 | 0.003 | -0.011 | -0.002 | 0.010 | 0.0056 | 98.682 |  |
| CHL1 | 8958_51 | LTL | 27 | Mode Based | 0.0017 | 0.005 | -0.007 | 0.011 | 0.713 | 0.0056 | 98.682 |  |
| CHL1 | 8958_51 | LTL | 27 | MR-PRESSO (Raw) | -0.0058 | 0.010 | -0.027 | 0.015 | 0.574 | 0.0056 | 98.682 |  |
| CHL1 | 8958_51 | LTL | 25 | MR-PRESSO (Outlier-Corrected) | -0.0008 | 0.004 | -0.009 | 0.007 | 0.826 | 0.0056 | 98.682 |  |
| * R² indicates variance explained by genetic instruments. ♰ F statistic represents the strength of the genetic instruments IVW= inverse-variance weighted method; MR-PRESSO (Outlier-Corrected) test was presented after exclusion of outlier SNPs if outliers were detected. | | | | | | | | | | | |  |
|  |  |  |  |  |  |  |  |  |  |  |  |  |

| **Table S15. Summary of Instrumental Variables Used in the Protein-LTL Backward Direction MR Analysis** | | | | | | | | |
| --- | --- | --- | --- | --- | --- | --- | --- | --- |
| **Protein** | **SNP** | **Effect Allele** | **Other Allele** | **Chr** | **Position (GRCh37)** | **Position (GRCh38)** | **R2*** | **F♰** |
| PLOD3 | rs149062332 | C | T | 3 | 186341025 | 186623236 | 6.69E-05 | 31.587 |
|  | rs9862378 | T | G | 3 | 58324394 | 58338667 | 6.38E-05 | 30.132 |
|  | rs11748446 | C | G | 5 | 172989605 | 173562602 | 1.03E-04 | 48.803 |
|  | rs13162614 | T | C | 5 | 173717503 | 174290500 | 1.04E-04 | 49.246 |
|  | rs17691833 | G | A | 5 | 173190787 | 173763784 | 7.02E-05 | 33.144 |
|  | rs72816176 | T | C | 5 | 172600842 | 173173839 | 6.86E-05 | 32.393 |
|  | rs80028711 | CTCTG | CTCTA | 5 | 172546325 | 173119318 | 1.04E-04 | 49.012 |
|  | rs117811651 | A | C | 7 | 98381716 | 98752404 | 1.64E-04 | 77.560 |
|  | rs13232865 | T | C | 7 | 99639825 | 100042202 | 8.01E-05 | 37.845 |
|  | rs140392759 | A | C | 7 | 103456708 | 103816261 | 7.58E-05 | 35.796 |
|  | rs142309161 | T | C | 7 | 100948025 | 101304744 | 2.81E-04 | 132.860 |
|  | rs143850556 | G | C | 7 | 96384345 | 96755033 | 2.56E-04 | 120.694 |
|  | rs144081584 | T | C | 7 | 101848404 | 102205124 | 1.57E-04 | 74.074 |
|  | rs144874182 | A | C | 7 | 96885388 | 97256076 | 1.09E-04 | 51.517 |
|  | rs147769251 | T | C | 7 | 94117829 | 94488517 | 9.11E-05 | 43.010 |
|  | rs150814238 | T | C | 7 | 100851777 | 101208496 | 7.86E-05 | 37.099 |
|  | rs191464857 | C | T | 7 | 97642549 | 98013237 | 2.63E-04 | 124.016 |
|  | rs212425 | A | C | 7 | 105514977 | 105874531 | 8.51E-05 | 40.186 |
|  | rs62465594 | T | C | 7 | 100991649 | 101348368 | 6.36E-05 | 30.038 |
|  | rs6962326 | TATATATATATACACACACACACACACACACACACAC | TATATATATACACACACACACACACACACACACACAC | 7 | 104560083 | 104919626 | 1.80E-04 | 85.050 |
|  | rs697392 | C | T | 7 | 98196687 | 98567375 | 6.86E-05 | 32.398 |
|  | rs73173919 | A | C | 7 | 101181089 | 101537808 | 1.47E-04 | 69.606 |
|  | rs73173931 | T | C | 7 | 101209730 | 101566450 | 1.19E-04 | 56.163 |
|  | rs77339260 | G | C | 7 | 97360330 | 97731018 | 2.25E-04 | 106.489 |
|  | rs7781473 | A | C | 7 | 105315318 | 105674871 | 6.37E-05 | 30.090 |
|  | rs2477642 | C | T | 10 | 17863037 | 17821038 | 1.23E-04 | 57.850 |
|  | rs372060974 | T | C | 10 | 18156657 | 17867728 | 7.30E-05 | 34.469 |
|  | rs565840574 | G | A | 10 | 17848398 | 17806399 | 1.13E-04 | 53.377 |
|  | rs71497225 | C | G | 10 | 18138665 | 17849736 | 1.13E-04 | 53.459 |
|  | rs7964859 | G | C | 12 | 102220783 | 101827005 | 9.03E-05 | 42.641 |
| COL28A1 | rs12126578 | C | A | 1 | 107490554 | 106947932 | 6.30E-05 | 29.757 |
|  | rs11677932 | A | G | 2 | 238223955 | 237315312 | 1.26E-04 | 59.422 |
|  | rs11678707 | G | C | 2 | 238306680 | 237398037 | 7.93E-05 | 37.433 |
|  | rs10254101 | T | C | 7 | 151415536 | 151718450 | 6.78E-05 | 31.996 |
|  | rs767298 | TAACTAAT | GAACTAAT | 7 | 7274584 | 7234953 | 6.94E-05 | 32.752 |
|  | rs2129775 | TACTAATCATCA | TACTAACCATCA | 15 | 53862699 | 53570496 | 6.56E-05 | 30.996 |
|  | rs12945299 | ACGCT | ACACT | 17 | 7080069 | 7176748 | 8.71E-05 | 41.122 |
|  | rs235366 | A | G | 21 | 46204085 | 44784170 | 6.85E-05 | 32.351 |
|  | rs3088026 | T | C | 21 | 47549613 | 46129699 | 9.43E-05 | 44.538 |
| SVEP1 | rs11264614 | T | C | 1 | 157053726 | 157083934 | 2.75E-04 | 130.030 |
|  | rs114889704 | G | T | 1 | 155534434 | 155564643 | 7.42E-05 | 35.024 |
|  | rs115770375 | G | C | 1 | 152812903 | 152840427 | 7.79E-05 | 36.779 |
|  | rs12031639 | T | C | 1 | 154650823 | 154678347 | 1.25E-04 | 59.053 |
|  | rs144800015 | CGGCCAGGCAGAGATGCTCCTCACCTCCCAGACGGGGTTGTGGCCGGGCAGAGG | CGGCCGGGCAGAGATGCTCCTCACCTCCCAGACGGGGTTGTGGCCGGGCAGAGG | 1 | 152444663 | 152472182 | 9.58E-05 | 45.231 |
|  | rs147639000 | A | G | 1 | 156878044 | 156908252 | 1.37E-03 | 650.064 |
|  | rs149541919 | A | G | 1 | 156623822 | 156654030 | 9.99E-05 | 47.193 |
|  | rs2494034 | C | A | 1 | 158842453 | 158872663 | 1.33E-04 | 62.817 |
|  | rs2795032 | G | C | 1 | 157552260 | 157582470 | 7.45E-05 | 35.201 |
|  | rs35603727 | A | G | 1 | 156007988 | 156038197 | 8.20E-05 | 38.726 |
|  | rs4110937 | A | G | 1 | 157479006 | 157509216 | 7.04E-05 | 33.236 |
|  | rs45444697 | G | C | 1 | 155034632 | 155062156 | 6.31E-04 | 298.319 |
|  | rs6698628 | T | C | 1 | 154721780 | 154749304 | 7.90E-05 | 37.315 |
|  | rs67195253 | T | C | 1 | 156796815 | 156827023 | 1.09E-04 | 51.373 |
|  | rs74617664 | C | T | 7 | 103591579 | 103951132 | 7.94E-05 | 37.476 |
|  | rs10759431 | AAACAC | AAACAA | 9 | 113167322 | 110405037 | 7.62E-04 | 360.004 |
|  | rs10816961 | G | A | 9 | 113049678 | 110287398 | 1.45E-04 | 68.592 |
|  | rs10980324 | C | T | 9 | 113081565 | 110319285 | 1.31E-04 | 61.765 |
|  | rs111413458 | T | A | 9 | 113954913 | 111192633 | 1.35E-04 | 63.773 |
|  | rs11244049 | ATGGCGGGGCTGGGCTGCAGTGGTGGGCTGGGCTGCAGTGG | ATGGTGGGGCTGGGCTGCAGTGGTGGGCTGGGCTGCAGTGG | 9 | 136123092 | 133247701 | 1.17E-04 | 55.220 |
|  | rs112639900 | C | T | 9 | 114397121 | 111634841 | 4.31E-04 | 203.544 |
|  | rs116878365 | C | T | 9 | 113476051 | 110713771 | 7.93E-05 | 37.456 |
|  | rs116923150 | A | G | 9 | 114876489 | 112114209 | 2.94E-04 | 138.751 |
|  | rs117638436 | C | T | 9 | 116064345 | 113302065 | 2.09E-04 | 98.637 |
|  | rs117781218 | G | A | 9 | 116337010 | 113574730 | 6.58E-05 | 31.058 |
|  | rs117920122 | A | G | 9 | 115481182 | 112718902 | 2.44E-04 | 115.350 |
|  | rs12350319 | T | C | 9 | 112518534 | 109756254 | 8.76E-05 | 41.379 |
|  | rs13285472 | G | A | 9 | 112734742 | 109972462 | 2.27E-04 | 107.091 |
|  | rs138764012 | A | G | 9 | 113155995 | 110393715 | 1.42E-04 | 67.006 |
|  | rs139854432 | G | A | 9 | 113386600 | 110624320 | 1.05E-03 | 497.354 |
|  | rs141937351 | A | C | 9 | 112600280 | 109838000 | 2.00E-04 | 94.420 |
|  | rs145419292 | T | C | 9 | 112441339 | 109679059 | 4.85E-04 | 228.992 |
|  | rs149834214 | T | C | 9 | 114231682 | 111469402 | 7.08E-05 | 33.423 |
|  | rs17806752 | T | C | 9 | 112962942 | 110200662 | 8.16E-05 | 38.552 |
|  | rs1888990 | G | C | 9 | 112771675 | 110009395 | 1.58E-04 | 74.679 |
|  | rs4483229 | A | G | 9 | 111980234 | 109217954 | 1.28E-04 | 60.526 |
|  | rs4571793 | G | A | 9 | 115891728 | 113129448 | 7.18E-05 | 33.920 |
|  | rs4978437 | G | A | 9 | 114069273 | 111306993 | 1.06E-04 | 49.874 |
|  | rs4978736 | A | G | 9 | 111524718 | 108762438 | 2.32E-04 | 109.556 |
|  | rs555000 | A | T | 9 | 113645403 | 110883123 | 2.71E-04 | 128.062 |
|  | rs60066776 | C | T | 9 | 114099687 | 111337407 | 3.34E-04 | 157.834 |
|  | rs62571298 | A | G | 9 | 113368340 | 110606060 | 2.92E-04 | 137.808 |
|  | rs62573124 | A | C | 9 | 113276603 | 110514323 | 1.85E-04 | 87.211 |
|  | rs62578882 | A | G | 9 | 112918083 | 110155803 | 8.01E-05 | 37.807 |
|  | rs7020788 | A | G | 9 | 113232979 | 110470699 | 1.11E-03 | 525.845 |
|  | rs72751879 | A | G | 9 | 113779575 | 111017295 | 1.14E-04 | 53.661 |
|  | rs72756534 | A | C | 9 | 113521503 | 110759223 | 4.04E-04 | 190.902 |
|  | rs72759341 | G | A | 9 | 113908189 | 111145909 | 2.71E-04 | 128.174 |
|  | rs72762039 | A | C | 9 | 114810114 | 112047834 | 1.81E-04 | 85.576 |
|  | rs75691100 | G | T | 9 | 110700041 | 107937760 | 6.68E-05 | 31.538 |
|  | rs75733144 | G | C | 9 | 116423446 | 113661166 | 1.96E-04 | 92.750 |
|  | rs75823710 | C | T | 9 | 112366890 | 109604610 | 8.07E-05 | 38.094 |
|  | rs75885967 | G | T | 9 | 111305676 | 108543396 | 6.96E-05 | 32.880 |
|  | rs77268922 | A | G | 9 | 116380938 | 113618658 | 7.64E-05 | 36.081 |
|  | rs7866874 | T | C | 9 | 112025944 | 109263664 | 1.48E-04 | 69.835 |
|  | rs78742138 | C | T | 9 | 113260708 | 110498428 | 2.51E-03 | 1188.927 |
|  | rs78776206 | A | G | 9 | 113765615 | 111003335 | 3.29E-04 | 155.471 |
|  | rs79950608 | T | C | 9 | 113635972 | 110873692 | 1.05E-04 | 49.529 |
|  | rs2923099 | A | C | 11 | 10369340 | 10347793 | 8.03E-05 | 37.917 |
|  | rs7952602 | C | G | 11 | 126233669 | 126363774 | 6.38E-05 | 30.114 |
|  | rs879620 | T | C | 16 | 4015729 | 3965728 | 6.71E-05 | 31.695 |
| MZB1 | rs114014780 | C | G | 3 | 57724341 | 57738614 | 4.33E-04 | 204.496 |
|  | rs58061357 | CAAAAAAAAAAC | CAAAAAAAAAAA | 3 | 56666513 | 56632474 | 7.47E-05 | 35.284 |
|  | rs6771011 | G | A | 3 | 57681084 | 57695357 | 2.45E-04 | 115.632 |
|  | rs6857005 | A | G | 4 | 57740281 | 56874115 | 7.58E-05 | 35.780 |
|  | rs115403270 | G | A | 5 | 137979481 | 138643792 | 7.95E-05 | 37.528 |
|  | rs144366368 | C | G | 5 | 139368466 | 139988881 | 1.13E-04 | 53.379 |
|  | rs184739217 | T | A | 5 | 138972624 | 139593039 | 1.41E-04 | 66.453 |
|  | rs186055462 | T | C | 5 | 136904641 | 137568952 | 1.22E-04 | 57.718 |
|  | rs189387183 | A | G | 5 | 138673281 | 139337592 | 5.27E-04 | 249.195 |
|  | rs62385338 | C | A | 5 | 139074693 | 139695108 | 9.03E-05 | 42.655 |
|  | rs2523515 | A | G | 6 | 31341887 | 31374110 | 1.62E-04 | 76.725 |
|  | rs28732146 | A | T | 6 | 31561353 | 31593576 | 7.23E-05 | 34.123 |
|  | rs4133115 | CTTCTTCTTG | CTTCTTCTTC | 6 | 139618369 | 139297223 | 8.90E-05 | 42.012 |
|  | rs60160049 | G | T | 6 | 32510208 | 32542431 | 8.61E-05 | 40.680 |
|  | rs2017544 | C | T | 7 | 76039448 | 76410131 | 8.14E-05 | 38.430 |
|  | rs2347784 | G | C | 7 | 6524843 | 6485212 | 2.91E-04 | 137.401 |
|  | rs73169671 | A | C | 7 | 150947689 | 151250603 | 8.04E-05 | 37.947 |
|  | rs836554 | T | C | 7 | 6445235 | 6405604 | 7.33E-05 | 34.630 |
|  | rs1983890 | T | C | 10 | 6178614 | 6136651 | 9.19E-05 | 43.383 |
|  | rs2497318 | T | C | 10 | 94432000 | 92672243 | 7.17E-05 | 33.868 |
|  | rs214080 | G | A | 11 | 17299762 | 17278215 | 2.30E-04 | 108.512 |
|  | rs7108992 | C | A | 11 | 128381867 | 128511972 | 7.16E-05 | 33.812 |
|  | rs9516445 | A | G | 13 | 95256891 | 94604637 | 8.85E-05 | 41.809 |
|  | rs11621634 | G | A | 14 | 106108271 | 105641934 | 2.95E-04 | 139.438 |
|  | rs2857374 | C | G | 14 | 106345181 | 105879323 | 7.26E-05 | 34.275 |
|  | rs709589 | C | T | 14 | 106306841 | 105840505 | 4.56E-04 | 215.576 |
|  | rs76915552 | T | C | 14 | 106393467 | 105927607 | 7.44E-05 | 35.113 |
|  | rs12439187 | C | T | 15 | 75052914 | 74760573 | 6.77E-05 | 31.963 |
|  | rs4792798 | A | G | 17 | 16840862 | 16937548 | 1.19E-04 | 56.325 |
|  | rs62073975 | A | G | 17 | 45622690 | 47545324 | 8.98E-05 | 42.418 |
|  | rs9901675 | A | G | 17 | 7484812 | 7581494 | 1.15E-04 | 54.086 |
|  | rs3177243 | C | G | 22 | 24179922 | 23837735 | 9.16E-05 | 43.260 |
| TNFRSF17 | rs12758915 | G | A | 1 | 227058344 | 226870643 | 7.60E-05 | 35.891 |
|  | rs13425999 | T | C | 2 | 33702203 | 33477136 | 7.44E-05 | 35.143 |
|  | rs4851257 | T | C | 2 | 100775297 | 100158835 | 7.31E-05 | 34.522 |
|  | rs6728684 | G | T | 2 | 26150773 | 25927904 | 9.89E-05 | 46.716 |
|  | rs4690163 | C | T | 4 | 975137 | 981349 | 1.47E-04 | 69.631 |
|  | rs113081750 | G | A | 6 | 364926 | 364926 | 6.69E-05 | 31.577 |
|  | rs1140809 | A | C | 6 | 30611676 | 30643899 | 1.16E-04 | 54.798 |
|  | rs115736104 | A | G | 6 | 267616 | 267616 | 1.01E-04 | 47.915 |
|  | rs1418708 | A | G | 6 | 205610 | 205610 | 7.19E-05 | 33.932 |
|  | rs2647071 | T | C | 6 | 32573910 | 32606133 | 8.89E-05 | 41.963 |
|  | rs510432 | C | T | 6 | 106774030 | 106326155 | 7.54E-05 | 35.602 |
|  | rs6942338 | T | C | 6 | 417104 | 417104 | 6.76E-05 | 31.921 |
|  | rs78377363 | C | T | 6 | 138125881 | 137804744 | 7.00E-05 | 33.062 |
|  | rs3778754 | G | C | 7 | 128575552 | 128935498 | 9.53E-05 | 45.020 |
|  | rs2532744 | G | T | 10 | 28902618 | 28613689 | 1.44E-04 | 68.071 |
|  | rs10141746 | G | A | 14 | 103237259 | 102770922 | 1.19E-04 | 56.344 |
|  | rs3803286 | GAGAG | GAGAA | 14 | 103246470 | 102780129 | 1.77E-04 | 83.743 |
|  | rs11570136 | A | T | 16 | 12058832 | 11964975 | 5.02E-04 | 236.984 |
|  | rs78083064 | T | A | 16 | 11821999 | 11728143 | 6.70E-05 | 31.619 |
|  | rs17334923 | T | C | 17 | 43827244 | 45749878 | 6.61E-05 | 31.220 |
|  | rs34562254 | A | G | 17 | 16842991 | 16939677 | 4.20E-04 | 198.517 |
|  | rs894872 | G | T | 17 | 77910974 | 79937175 | 7.21E-05 | 34.039 |
|  | rs72939458 | A | T | 18 | 60765648 | 63098415 | 9.04E-05 | 42.682 |
|  | rs10048885 | G | A | 22 | 30580252 | 30184263 | 7.77E-05 | 36.677 |
| KDR | rs144426992 | G | A | 1 | 161569893 | 161600103 | 7.88E-05 | 37.191 |
|  | rs61830291 | C | A | 1 | 221001142 | 220827800 | 8.36E-05 | 39.464 |
|  | rs34211178 | A | G | 3 | 98383562 | 98664718 | 7.79E-04 | 367.944 |
|  | rs116530298 | A | G | 4 | 55966759 | 55100592 | 1.31E-04 | 61.819 |
|  | rs12500575 | T | C | 4 | 55765735 | 54899569 | 6.76E-05 | 31.911 |
|  | rs138087021 | T | C | 4 | 55939452 | 55073285 | 1.06E-04 | 49.908 |
|  | rs149642376 | T | A | 4 | 56463610 | 55597443 | 2.65E-04 | 124.930 |
|  | rs17711073 | C | T | 4 | 55978160 | 55111993 | 7.67E-05 | 36.211 |
|  | rs2305948 | T | C | 4 | 55979558 | 55113391 | 8.71E-04 | 411.391 |
|  | rs2634465 | G | A | 4 | 56532616 | 55666449 | 2.02E-04 | 95.304 |
|  | rs34231037 | G | A | 4 | 55972946 | 55106779 | 2.28E-03 | 1076.891 |
|  | rs35297343 | C | T | 4 | 57963121 | 57096955 | 7.74E-05 | 36.551 |
|  | rs55713360 | CGG | CGA | 4 | 55992067 | 55125898 | 4.81E-04 | 227.448 |
|  | rs62297731 | T | C | 4 | 54654363 | 53788196 | 7.60E-05 | 35.878 |
|  | rs62304389 | T | C | 4 | 55631105 | 54764939 | 9.53E-05 | 45.006 |
|  | rs66984899 | G | A | 4 | 56781913 | 55915747 | 6.82E-05 | 32.186 |
|  | rs6853369 | TTTG | TTTA | 4 | 56503322 | 55637152 | 6.46E-05 | 30.507 |
|  | rs74807145 | T | G | 4 | 56456565 | 55590398 | 8.53E-05 | 40.260 |
|  | rs112625334 | C | T | 6 | 31473353 | 31505576 | 7.15E-05 | 33.747 |
|  | rs187570181 | T | C | 6 | 32164587 | 32196810 | 9.52E-05 | 44.970 |
|  | rs434841 | A | G | 6 | 32191041 | 32223264 | 9.40E-05 | 44.365 |
|  | rs8192585 | A | G | 6 | 32188823 | 32221046 | 1.29E-04 | 61.090 |
|  | rs35783704 | A | G | 8 | 105966258 | 104954030 | 6.47E-05 | 30.548 |
|  | rs6993770 | T | A | 8 | 106581528 | 105569300 | 6.46E-05 | 30.515 |
|  | rs10120389 | CGCCTGTGGCCTGTGGCCTGC | CGCCTGTGGCCTGTGGCCTGT | 9 | 135372852 | 132497445 | 6.71E-05 | 31.702 |
|  | rs112471944 | T | C | 9 | 135117742 | 132242355 | 6.47E-05 | 30.549 |
|  | rs117537850 | C | T | 9 | 136255929 | 133390145 | 3.08E-04 | 145.708 |
|  | rs117608058 | A | G | 9 | 136405404 | 133540282 | 1.10E-04 | 51.784 |
|  | rs146798134 | AATTGTGCCACTGCG | AATTGTGCCACTGCA | 9 | 135783439 | 132908038 | 2.11E-04 | 99.425 |
|  | rs17150482 | C | T | 9 | 136194595 | 133327758 | 3.32E-04 | 156.971 |
|  | rs2491103 | AAAATAAAT | AATATAAAT | 9 | 130313821 | 127551540 | 6.71E-05 | 31.689 |
|  | rs28463601 | A | G | 9 | 136374896 | 133509774 | 2.99E-04 | 141.054 |
|  | rs3858099 | C | T | 9 | 136684952 | 133819830 | 1.05E-04 | 49.389 |
|  | rs55710199 | T | C | 9 | 136376405 | 133511283 | 1.96E-04 | 92.740 |
|  | rs569956268 | C | G | 9 | 136575551 | 133710429 | 7.76E-05 | 36.640 |
|  | rs576123 | T | C | 9 | 136144308 | 133268896 | 1.96E-03 | 927.537 |
|  | rs579232 | G | T | 9 | 136599763 | 133734641 | 6.54E-05 | 30.878 |
|  | rs72759433 | ATGAC | GTGAC | 9 | 135770154 | 132894767 | 7.32E-05 | 34.562 |
|  | rs75305750 | A | G | 9 | 135982933 | 133107546 | 7.95E-05 | 37.537 |
|  | rs8176757 | C | A | 9 | 136130012 | 133254625 | 3.77E-04 | 178.023 |
|  | rs112564808 | C | T | 11 | 126047190 | 126177295 | 6.40E-05 | 30.229 |
|  | rs117603193 | G | A | 11 | 126260864 | 126390969 | 8.56E-05 | 40.426 |
|  | rs145418098 | A | G | 11 | 125986048 | 126116153 | 6.82E-05 | 32.198 |
|  | rs16921960 | C | A | 11 | 95188894 | 95455730 | 8.72E-05 | 41.194 |
|  | rs35166255 | A | G | 11 | 126301756 | 126431861 | 4.04E-04 | 190.898 |
|  | rs143501783 | C | T | 17 | 7800319 | 7897001 | 7.65E-05 | 36.139 |
|  | rs149151704 | T | C | 17 | 81008138 | 83050262 | 7.29E-05 | 34.411 |
|  | rs62073443 | C | T | 17 | 81007175 | 83049299 | 7.09E-05 | 33.476 |
| GDF15 | rs10424678 | C | T | 19 | 18649696 | 18538886 | 1.00E-04 | 47.366 |
|  | rs111615037 | A | G | 19 | 18569910 | 18459100 | 1.83E-04 | 86.425 |
|  | rs112949659 | A | G | 19 | 18739895 | 18629085 | 2.98E-04 | 140.823 |
|  | rs113700483 | A | G | 19 | 18494508 | 18383698 | 7.76E-04 | 366.602 |
|  | rs114681259 | T | G | 19 | 19269428 | 19158619 | 1.54E-04 | 72.501 |
|  | rs115605748 | C | A | 19 | 30364078 | 29873171 | 6.35E-05 | 29.991 |
|  | rs11673146 | A | G | 19 | 18356779 | 18245969 | 8.12E-05 | 38.366 |
|  | rs116851292 | G | A | 19 | 20083600 | 19972791 | 7.05E-05 | 33.313 |
|  | rs117226460 | C | G | 19 | 22807397 | 22624595 | 7.93E-05 | 37.424 |
|  | rs117427429 | G | T | 19 | 18910904 | 18800095 | 9.13E-05 | 43.120 |
|  | rs11880730 | C | T | 19 | 20611728 | 20428922 | 1.05E-04 | 49.508 |
|  | rs1227731 | A | G | 19 | 18497903 | 18387093 | 1.39E-03 | 657.251 |
|  | rs12462034 | C | T | 19 | 18475226 | 18364416 | 2.28E-03 | 1078.409 |
|  | rs12982058 | T | C | 19 | 17409380 | 17298571 | 6.94E-05 | 32.757 |
|  | rs138086622 | T | A | 19 | 18634669 | 18523859 | 1.03E-04 | 48.536 |
|  | rs138462992 | G | A | 19 | 18928265 | 18817456 | 1.30E-04 | 61.254 |
|  | rs142320622 | A | G | 19 | 19067921 | 18957112 | 1.71E-04 | 80.610 |
|  | rs144144745 | T | C | 19 | 18438942 | 18328132 | 9.72E-05 | 45.912 |
|  | rs145653034 | C | T | 19 | 17764329 | 17653520 | 7.13E-05 | 33.655 |
|  | rs147105422 | A | G | 19 | 17833468 | 17722659 | 8.41E-05 | 39.710 |
|  | rs147706800 | A | G | 19 | 18254108 | 18143298 | 1.21E-04 | 57.169 |
|  | rs148038170 | A | G | 19 | 18475304 | 18364494 | 8.93E-05 | 42.192 |
|  | rs149390855 | A | G | 19 | 18493608 | 18382798 | 9.63E-05 | 45.495 |
|  | rs16982307 | A | G | 19 | 18421690 | 18310880 | 3.42E-04 | 161.388 |
|  | rs1962457 | A | G | 19 | 20447879 | 20337070 | 1.11E-04 | 52.572 |
|  | rs2335890 | G | A | 19 | 20145347 | 20034538 | 8.35E-05 | 39.440 |
|  | rs2435000 | C | T | 19 | 22011115 | 21828313 | 1.36E-04 | 64.113 |
|  | rs2701307 | C | T | 19 | 20589758 | 20406936 | 7.76E-05 | 36.647 |
|  | rs34500783 | C | A | 19 | 19878197 | 19767388 | 1.08E-04 | 51.208 |
|  | rs45485597 | A | G | 19 | 17025311 | 16914501 | 6.95E-05 | 32.835 |
|  | rs533828592 | GC | GT | 19 | 18448024 | 18337213 | 2.59E-04 | 122.364 |
|  | rs55801550 | T | C | 19 | 21319386 | 21136583 | 9.22E-05 | 43.535 |
|  | rs55836620 | C | T | 19 | 22045943 | 21863141 | 8.69E-05 | 41.023 |
|  | rs56089672 | A | G | 19 | 17252410 | 17141600 | 1.59E-04 | 75.015 |
|  | rs62118195 | C | G | 19 | 16820015 | 16709204 | 8.16E-05 | 38.554 |
|  | rs62121123 | C | T | 19 | 18166559 | 18055749 | 1.33E-04 | 62.814 |
|  | rs72995433 | C | T | 19 | 18693972 | 18583162 | 6.98E-05 | 32.948 |
|  | rs73009453 | T | C | 19 | 18483859 | 18373049 | 2.98E-04 | 140.799 |
|  | rs73923156 | G | A | 19 | 18844416 | 18733606 | 6.97E-05 | 32.904 |
|  | rs75089010 | A | G | 19 | 18802617 | 18691807 | 2.63E-04 | 124.308 |
|  | rs77752345 | T | C | 19 | 18413773 | 18302963 | 1.49E-04 | 70.478 |
|  | rs78258728 | A | G | 19 | 18848829 | 18738019 | 6.53E-05 | 30.820 |
|  | rs8110571 | A | G | 19 | 17692929 | 17582120 | 7.63E-05 | 36.021 |
| GP1Bα | rs1434282 | T | C | 1 | 199010721 | 199041592 | 1.34E-04 | 63.165 |
|  | rs3767809 | C | T | 1 | 118154831 | 117612209 | 8.09E-05 | 38.186 |
|  | rs62175229 | T | C | 2 | 160677559 | 159821048 | 1.05E-04 | 49.644 |
|  | rs114124975 | T | C | 3 | 183266913 | 183549125 | 6.65E-05 | 31.399 |
|  | rs56407378 | T | G | 3 | 142803974 | 143085132 | 9.14E-05 | 43.181 |
|  | rs7428843 | T | C | 3 | 143023597 | 143304755 | 6.61E-05 | 31.216 |
|  | rs78565404 | T | C | 3 | 184090242 | 184372454 | 1.16E-04 | 54.786 |
|  | rs952982 | T | G | 3 | 184089572 | 184371784 | 6.56E-05 | 30.964 |
|  | rs114694170 | C | T | 5 | 88180196 | 88884379 | 9.07E-05 | 42.816 |
|  | rs11759553 | T | A | 6 | 135422296 | 135101158 | 1.81E-04 | 85.647 |
|  | rs28590669 | G | A | 6 | 32621443 | 32653666 | 8.66E-05 | 40.889 |
|  | rs819441 | G | T | 7 | 80222614 | 80593298 | 7.35E-05 | 34.721 |
|  | rs10110559 | T | C | 8 | 11264598 | 11407089 | 6.40E-05 | 30.225 |
|  | rs11998274 | C | G | 8 | 9139828 | 9282318 | 7.77E-05 | 36.675 |
|  | rs6993770 | T | A | 8 | 106581528 | 105569300 | 3.13E-04 | 147.851 |
|  | rs12005199 | A | G | 9 | 4763491 | 4763491 | 2.97E-04 | 140.166 |
|  | rs461068 | G | C | 9 | 4835827 | 4835827 | 1.33E-04 | 62.699 |
|  | rs10876913 | A | G | 12 | 57044670 | 56650886 | 8.41E-05 | 39.731 |
|  | rs3184504 | C | T | 12 | 111884608 | 111446804 | 5.80E-04 | 273.848 |
|  | rs1555405 | A | G | 14 | 101176769 | 100710432 | 1.42E-04 | 66.982 |
|  | rs117107082 | T | C | 17 | 4745237 | 4841942 | 9.75E-05 | 46.028 |
|  | rs138959040 | A | G | 17 | 4500387 | 4597092 | 2.90E-04 | 137.119 |
|  | rs140353756 | A | G | 17 | 3546665 | 3643371 | 1.09E-04 | 51.299 |
|  | rs148477713 | G | C | 17 | 3409598 | 3506304 | 1.17E-04 | 55.114 |
|  | rs17834140 | T | C | 17 | 55465771 | 57388410 | 7.17E-05 | 33.846 |
|  | rs185009887 | A | G | 17 | 5751652 | 5848332 | 1.97E-04 | 93.134 |
|  | rs72832028 | G | A | 17 | 4502961 | 4599666 | 6.50E-05 | 30.715 |
|  | rs72835078 | T | G | 17 | 4826592 | 4923297 | 6.18E-04 | 291.913 |
|  | rs77185535 | C | G | 17 | 4796441 | 4893146 | 2.27E-04 | 107.063 |
| THPO | rs1706417 | G | A | 3 | 165448909 | 165731121 | 8.56E-05 | 40.424 |
|  | rs9389278 | C | T | 6 | 135525927 | 135204789 | 7.26E-05 | 34.273 |
|  | rs6993770 | T | A | 8 | 106581528 | 105569300 | 1.12E-04 | 52.871 |
|  | rs11248395 | T | G | 10 | 124871148 | 123111632 | 6.75E-05 | 31.876 |
|  | rs16978104 | A | G | 18 | 42164823 | 44584858 | 6.31E-05 | 29.802 |
|  | rs17758695 | T | C | 18 | 60920854 | 63253621 | 7.37E-05 | 34.816 |
| ST3GAL6 | rs111274194 | A | T | 3 | 104480675 | 104761831 | 1.08E-04 | 50.880 |
|  | rs111750967 | T | C | 3 | 72600620 | 72551469 | 6.31E-05 | 29.803 |
|  | rs111820011 | G | T | 3 | 97414096 | 97695252 | 1.05E-04 | 49.785 |
|  | rs112623189 | A | G | 3 | 77193837 | 77144686 | 1.72E-04 | 81.207 |
|  | rs112689780 | G | A | 3 | 76934458 | 76885307 | 1.25E-04 | 59.128 |
|  | rs113073961 | TACGAAGGAC | TACGAAGGAT | 3 | 94607985 | 94889132 | 1.20E-04 | 56.496 |
|  | rs113764913 | A | T | 3 | 111243729 | 111524882 | 1.10E-04 | 51.799 |
|  | rs113851417 | C | T | 3 | 99846802 | 100127958 | 9.17E-05 | 43.306 |
|  | rs114051017 | T | C | 3 | 94653379 | 94934535 | 1.38E-04 | 65.362 |
|  | rs114432877 | G | A | 3 | 104707278 | 104988434 | 8.99E-05 | 42.465 |
|  | rs114521356 | T | C | 3 | 94424576 | 94705732 | 1.63E-04 | 77.045 |
|  | rs114628728 | T | G | 3 | 86200887 | 86151737 | 1.20E-04 | 56.613 |
|  | rs114701373 | G | C | 3 | 104907857 | 105189013 | 6.70E-05 | 31.652 |
|  | rs114745468 | T | A | 3 | 94691400 | 94972556 | 2.07E-04 | 97.841 |
|  | rs114773642 | C | G | 3 | 86542718 | 86493568 | 1.48E-04 | 70.107 |
|  | rs114804042 | T | G | 3 | 104413164 | 104694320 | 2.86E-04 | 135.130 |
|  | rs114833766 | C | T | 3 | 105702486 | 105983639 | 1.23E-04 | 57.906 |
|  | rs115056441 | A | T | 3 | 98650522 | 98931678 | 1.28E-02 | 6118.791 |
|  | rs115149735 | C | T | 3 | 103566784 | 103847940 | 6.62E-05 | 31.249 |
|  | rs115426710 | T | A | 3 | 96755953 | 97037109 | 2.32E-04 | 109.396 |
|  | rs115840193 | T | C | 3 | 101722882 | 102004038 | 8.51E-05 | 40.188 |
|  | rs115903975 | A | C | 3 | 98884735 | 99165891 | 4.20E-04 | 198.221 |
|  | rs115908492 | C | T | 3 | 93631140 | 93912296 | 3.82E-04 | 180.543 |
|  | rs115920108 | A | G | 3 | 100656639 | 100937795 | 2.38E-04 | 112.240 |
|  | rs116226749 | T | C | 3 | 94081481 | 94362637 | 2.98E-04 | 140.815 |
|  | rs116491677 | T | G | 3 | 97519047 | 97800203 | 1.70E-04 | 80.462 |
|  | rs116497138 | T | G | 3 | 87229633 | 87180483 | 9.90E-05 | 46.734 |
|  | rs116560349 | T | G | 3 | 82094406 | 82045255 | 9.22E-05 | 43.559 |
|  | rs116589363 | T | A | 3 | 82260477 | 82211326 | 1.40E-04 | 66.188 |
|  | rs116701466 | TAC | TAT | 3 | 108705702 | 108986853 | 1.26E-04 | 59.320 |
|  | rs11924482 | A | C | 3 | 100817478 | 101098634 | 3.37E-04 | 159.158 |
|  | rs12152427 | CTTTCATTTTTTTTTTC | TTTTCATTTTTTTTTTC | 3 | 100530925 | 100812081 | 4.36E-04 | 206.063 |
|  | rs12486063 | G | A | 3 | 103920627 | 104201783 | 2.36E-04 | 111.678 |
|  | rs12491293 | T | C | 3 | 99157982 | 99439138 | 5.33E-04 | 251.624 |
|  | rs12630951 | TACTTTTTTTT | TACTTTTTTTG | 3 | 107073273 | 107354416 | 6.30E-05 | 29.746 |
|  | rs13082154 | T | C | 3 | 107745398 | 108026551 | 6.60E-05 | 31.160 |
|  | rs13082870 | C | T | 3 | 101943151 | 102224307 | 6.45E-05 | 30.449 |
|  | rs13098840 | A | G | 3 | 93840234 | 94121390 | 7.76E-05 | 36.629 |
|  | rs13318932 | T | C | 3 | 98396350 | 98677506 | 2.81E-03 | 1330.899 |
|  | rs13353434 | C | T | 3 | 77219956 | 77170805 | 8.65E-05 | 40.841 |
|  | rs138207568 | A | C | 3 | 97190119 | 97471275 | 1.59E-04 | 75.219 |
|  | rs138945476 | T | A | 3 | 77106616 | 77057465 | 9.77E-05 | 46.130 |
|  | rs139959146 | A | G | 3 | 87245162 | 87196012 | 1.00E-04 | 47.220 |
|  | rs140078112 | A | G | 3 | 99020813 | 99301969 | 3.47E-04 | 163.757 |
|  | rs140410634 | T | C | 3 | 100188197 | 100469353 | 2.77E-04 | 130.771 |
|  | rs140472724 | A | G | 3 | 90212054 | 90162904 | 8.68E-05 | 40.979 |
|  | rs140628273 | G | A | 3 | 105431232 | 105712388 | 8.92E-05 | 42.135 |
|  | rs141138401 | T | C | 3 | 100548733 | 100829889 | 8.46E-04 | 399.644 |
|  | rs141299020 | A | G | 3 | 90216379 | 90167229 | 2.28E-04 | 107.845 |
|  | rs141356139 | A | G | 3 | 88863146 | 88813996 | 6.55E-05 | 30.918 |
|  | rs142167693 | T | C | 3 | 100778443 | 101059599 | 5.38E-04 | 254.093 |
|  | rs142435650 | A | G | 3 | 101707036 | 101988192 | 7.26E-04 | 343.166 |
|  | rs142514836 | T | G | 3 | 97262981 | 97544137 | 1.28E-04 | 60.260 |
|  | rs142626331 | C | A | 3 | 109016899 | 109298052 | 7.43E-05 | 35.069 |
|  | rs142641622 | C | T | 3 | 106877592 | 107158745 | 1.26E-04 | 59.284 |
|  | rs142783466 | G | A | 3 | 97972314 | 98253470 | 3.82E-04 | 180.574 |
|  | rs143195167 | T | C | 3 | 75524057 | 75474906 | 9.87E-05 | 46.588 |
|  | rs143504407 | C | A | 3 | 101986085 | 102267241 | 3.39E-04 | 159.995 |
|  | rs144131524 | A | G | 3 | 99064158 | 99345314 | 2.26E-03 | 1069.446 |
|  | rs144386867 | G | C | 3 | 81059657 | 81010506 | 8.49E-05 | 40.112 |
|  | rs1447834 | A | G | 3 | 79027354 | 78978204 | 1.03E-04 | 48.549 |
|  | rs144946806 | A | G | 3 | 89776219 | 89727069 | 3.45E-04 | 162.948 |
|  | rs144993658 | T | C | 3 | 104858620 | 105139776 | 1.78E-04 | 83.847 |
|  | rs145121727 | A | T | 3 | 80209154 | 80160004 | 7.44E-05 | 35.121 |
|  | rs145208113 | A | G | 3 | 103710137 | 103991293 | 2.16E-04 | 102.225 |
|  | rs145507768 | T | C | 3 | 97893017 | 98174173 | 7.45E-05 | 35.202 |
|  | rs145556095 | T | C | 3 | 76971190 | 76922039 | 2.15E-04 | 101.631 |
|  | rs146499133 | C | G | 3 | 84155329 | 84106178 | 1.91E-04 | 90.228 |
|  | rs146548838 | G | C | 3 | 83700635 | 83651484 | 9.37E-05 | 44.268 |
|  | rs146628847 | A | G | 3 | 78273390 | 78224240 | 1.44E-04 | 68.223 |
|  | rs146774454 | A | G | 3 | 87197879 | 87148729 | 2.88E-04 | 136.028 |
|  | rs146902326 | G | A | 3 | 98180149 | 98461305 | 9.89E-04 | 467.212 |
|  | rs147025965 | G | A | 3 | 83274121 | 83224970 | 8.73E-05 | 41.202 |
|  | rs147080039 | A | G | 3 | 77872665 | 77823514 | 3.30E-04 | 155.945 |
|  | rs147232533 | C | T | 3 | 97826961 | 98108117 | 4.28E-04 | 202.354 |
|  | rs147510222 | G | A | 3 | 100166212 | 100447368 | 2.36E-04 | 111.593 |
|  | rs147551296 | G | A | 3 | 84546853 | 84497702 | 8.59E-05 | 40.581 |
|  | rs147673991 | A | C | 3 | 107674251 | 107955404 | 1.44E-04 | 68.217 |
|  | rs148119505 | C | A | 3 | 80353670 | 80304520 | 1.57E-04 | 74.155 |
|  | rs148276704 | A | G | 3 | 110113854 | 110395007 | 7.29E-05 | 34.433 |
|  | rs148410612 | G | A | 3 | 78079900 | 78030749 | 7.33E-05 | 34.633 |
|  | rs149048756 | A | G | 3 | 104942499 | 105223655 | 1.18E-04 | 55.582 |
|  | rs149222170 | A | G | 3 | 106179326 | 106460479 | 1.33E-04 | 62.783 |
|  | rs149517005 | A | G | 3 | 97842355 | 98123511 | 2.01E-03 | 953.076 |
|  | rs150215452 | T | C | 3 | 95707048 | 95988204 | 2.65E-04 | 124.981 |
|  | rs150626861 | A | G | 3 | 81796818 | 81747667 | 8.08E-05 | 38.137 |
|  | rs150911169 | T | C | 3 | 74919823 | 74870672 | 7.38E-05 | 34.846 |
|  | rs151014187 | T | C | 3 | 90038677 | 89989527 | 3.55E-04 | 167.827 |
|  | rs151057489 | G | A | 3 | 79070710 | 79021560 | 6.55E-04 | 309.631 |
|  | rs151178105 | T | C | 3 | 101856527 | 102137683 | 2.38E-04 | 112.433 |
|  | rs151184523 | ATTATTCC | ATTACTCC | 3 | 97763189 | 98044341 | 8.61E-05 | 40.661 |
|  | rs1666130 | T | C | 3 | 77598943 | 77549792 | 1.18E-04 | 55.725 |
|  | rs17018841 | A | T | 3 | 81412517 | 81363366 | 1.21E-04 | 57.171 |
|  | rs180767589 | A | G | 3 | 109803823 | 110084976 | 6.89E-05 | 32.519 |
|  | rs181434690 | A | G | 3 | 95378088 | 95659244 | 5.01E-04 | 236.553 |
|  | rs1851817 | C | A | 3 | 98818848 | 99100004 | 3.12E-04 | 147.291 |
|  | rs185676730 | G | A | 3 | 114629471 | 114910624 | 1.00E-04 | 47.219 |
|  | rs188248000 | A | G | 3 | 79294259 | 79245109 | 6.33E-05 | 29.870 |
|  | rs188584451 | A | T | 3 | 98885464 | 99166620 | 1.69E-03 | 798.265 |
|  | rs189852791 | T | C | 3 | 81129509 | 81080358 | 2.63E-04 | 124.141 |
|  | rs189990093 | A | G | 3 | 112092092 | 112373245 | 7.74E-05 | 36.552 |
|  | rs190209276 | A | G | 3 | 80338584 | 80289434 | 1.59E-04 | 75.192 |
|  | rs1921444 | A | G | 3 | 106028694 | 106309847 | 9.42E-05 | 44.494 |
|  | rs192921606 | C | T | 3 | 88943901 | 88894751 | 3.83E-04 | 180.889 |
|  | rs2291377 | T | C | 3 | 105219699 | 105500855 | 1.28E-04 | 60.567 |
|  | rs2470704 | T | A | 3 | 105662998 | 105944151 | 1.13E-04 | 53.470 |
|  | rs2673460 | C | T | 3 | 104175286 | 104456442 | 9.04E-05 | 42.696 |
|  | rs28415035 | A | G | 3 | 75380286 | 75331135 | 8.46E-05 | 39.965 |
|  | rs28545169 | T | C | 3 | 106087917 | 106369070 | 1.01E-04 | 47.658 |
|  | rs291961 | A | G | 3 | 86511758 | 86462608 | 1.38E-04 | 65.096 |
|  | rs34220515 | T | G | 3 | 82522696 | 82473545 | 7.69E-05 | 36.319 |
|  | rs34685370 | C | G | 3 | 104661374 | 104942530 | 8.29E-05 | 39.170 |
|  | rs35546434 | G | A | 3 | 109768946 | 110050099 | 6.83E-05 | 32.232 |
|  | rs377511918 | TTTTGTGTGTGTGTGTGTGT | TTGTGTGTGTGTGTGTGTGT | 3 | 101548725 | 101829879 | 4.32E-04 | 204.153 |
|  | rs3929577 | TT | TA | 3 | 90117270 | 90068119 | 1.78E-04 | 84.023 |
|  | rs4317102 | C | A | 3 | 93971276 | 94252432 | 1.44E-04 | 68.183 |
|  | rs4677001 | A | T | 3 | 75396818 | 75347667 | 7.90E-05 | 37.314 |
|  | rs4857416 | G | A | 3 | 98718645 | 98999801 | 6.31E-04 | 297.946 |
|  | rs4858926 | A | G | 3 | 88030545 | 87981395 | 2.87E-04 | 135.476 |
|  | rs5019886 | CTCTCTG | CTCTCTC | 3 | 89042817 | 88993661 | 1.16E-04 | 54.721 |
|  | rs527846263 | C | G | 3 | 94558037 | 94839193 | 7.61E-05 | 35.928 |
|  | rs532033470 | TGGGGGGG | GGGGGGGG | 3 | 77677599 | 77628448 | 1.54E-04 | 72.962 |
|  | rs553150038 | G | A | 3 | 85403535 | 85354385 | 2.10E-04 | 99.386 |
|  | rs55856242 | T | C | 3 | 101249096 | 101530252 | 2.99E-04 | 141.270 |
|  | rs55975409 | T | C | 3 | 103796994 | 104078150 | 6.33E-05 | 29.889 |
|  | rs565570751 | TTATAAATAATGTG | TTATAAATAATGTA | 3 | 96692999 | 96974142 | 1.06E-04 | 49.846 |
|  | rs57195901 | C | T | 3 | 78341249 | 78292099 | 2.00E-04 | 94.477 |
|  | rs59260753 | A | T | 3 | 104883628 | 105164784 | 1.67E-04 | 78.652 |
|  | rs62248916 | G | A | 3 | 74135283 | 74086132 | 8.47E-05 | 40.015 |
|  | rs62254257 | A | T | 3 | 84699028 | 84649877 | 1.38E-04 | 65.353 |
|  | rs62254531 | C | T | 3 | 77926394 | 77877243 | 9.25E-05 | 43.679 |
|  | rs62262054 | T | C | 3 | 86371797 | 86322647 | 7.78E-05 | 36.744 |
|  | rs62263020 | CACATATATACATACACATATATACATACATACACATATATACATATATACACATATACACGTATATACACATATACACGTATATACATATATACACA | CACATATATACATACACATATATACATACATACACATATATACATATATACACATATACACGTATATACATATATACACGTATATACATATATACACA | 3 | 105342609 | 105623695 | 2.75E-04 | 129.931 |
|  | rs62263455 | T | C | 3 | 95466276 | 95747432 | 1.26E-04 | 59.699 |
|  | rs62271539 | G | A | 3 | 88836415 | 88787265 | 2.05E-04 | 96.655 |
|  | rs62282889 | G | A | 3 | 101035058 | 101316214 | 2.18E-04 | 103.131 |
|  | rs6549079 | GCTTCATG | ACTTCATG | 3 | 86200984 | 86151834 | 8.50E-05 | 40.130 |
|  | rs6766689 | C | T | 3 | 103508773 | 103789929 | 5.82E-04 | 274.877 |
|  | rs6777370 | G | T | 3 | 89696749 | 89647599 | 1.77E-04 | 83.723 |
|  | rs6807851 | C | A | 3 | 104278733 | 104559889 | 1.45E-04 | 68.462 |
|  | rs6810052 | G | A | 3 | 106761147 | 107042300 | 6.33E-05 | 29.878 |
|  | rs691307 | C | T | 3 | 96245795 | 96526951 | 1.06E-04 | 50.038 |
|  | rs71311332 | A | G | 3 | 97418346 | 97699502 | 1.96E-04 | 92.707 |
|  | rs71311389 | A | G | 3 | 98201544 | 98482700 | 1.30E-04 | 61.254 |
|  | rs72923352 | T | C | 3 | 94490855 | 94772011 | 2.93E-04 | 138.463 |
|  | rs73104113 | T | C | 3 | 69628923 | 69579772 | 7.73E-05 | 36.481 |
|  | rs73130751 | G | A | 3 | 85306528 | 85257378 | 3.22E-04 | 152.062 |
|  | rs73134215 | G | A | 3 | 87006981 | 86957831 | 1.45E-04 | 68.575 |
|  | rs73142972 | T | C | 3 | 97989394 | 98270550 | 2.89E-04 | 136.671 |
|  | rs73145861 | G | C | 3 | 93625910 | 93907066 | 2.34E-04 | 110.329 |
|  | rs73148319 | A | G | 3 | 99933870 | 100215026 | 1.14E-04 | 53.703 |
|  | rs73150411 | G | A | 3 | 102217329 | 102498485 | 4.80E-04 | 226.620 |
|  | rs73153256 | C | T | 3 | 89712244 | 89663094 | 4.51E-04 | 212.858 |
|  | rs73155483 | T | C | 3 | 102021837 | 102302993 | 3.33E-04 | 157.146 |
|  | rs73177456 | T | C | 3 | 105076941 | 105358097 | 1.31E-04 | 62.003 |
|  | rs73187672 | A | G | 3 | 104669086 | 104950242 | 1.84E-04 | 86.991 |
|  | rs74321252 | A | G | 3 | 107323104 | 107604257 | 6.41E-05 | 30.272 |
|  | rs7433284 | A | G | 3 | 42872590 | 42831098 | 1.13E-04 | 53.466 |
|  | rs7433331 | G | A | 3 | 100156704 | 100437860 | 1.75E-04 | 82.717 |
|  | rs74356132 | T | C | 3 | 81412281 | 81363130 | 8.31E-05 | 39.247 |
|  | rs74551591 | G | C | 3 | 74580484 | 74531333 | 1.12E-04 | 52.717 |
|  | rs74588001 | G | T | 3 | 95543699 | 95824855 | 4.27E-04 | 201.547 |
|  | rs74589988 | T | C | 3 | 96098343 | 96379499 | 1.19E-04 | 56.074 |
|  | rs74928832 | T | C | 3 | 85086455 | 85037304 | 1.54E-04 | 72.725 |
|  | rs75221418 | A | G | 3 | 82503480 | 82454329 | 7.89E-05 | 37.263 |
|  | rs75344916 | T | C | 3 | 101743276 | 102024432 | 2.53E-04 | 119.481 |
|  | rs75936087 | A | G | 3 | 77750204 | 77701053 | 8.18E-05 | 38.609 |
|  | rs75972094 | G | A | 3 | 107677801 | 107958954 | 6.53E-05 | 30.850 |
|  | rs76061304 | C | T | 3 | 95123759 | 95404915 | 2.18E-04 | 102.760 |
|  | rs7614193 | T | C | 3 | 87591859 | 87542709 | 9.99E-05 | 47.164 |
|  | rs76269162 | A | G | 3 | 100843284 | 101124440 | 6.61E-05 | 31.227 |
|  | rs76625599 | TG | TT | 3 | 79494353 | 79445202 | 9.25E-05 | 43.665 |
|  | rs76699394 | T | A | 3 | 102242696 | 102523852 | 1.49E-04 | 70.392 |
|  | rs76770215 | A | G | 3 | 103887421 | 104168577 | 1.08E-04 | 50.848 |
|  | rs76781898 | G | T | 3 | 94323227 | 94604383 | 6.58E-05 | 31.056 |
|  | rs76836886 | C | T | 3 | 79586003 | 79536853 | 2.40E-04 | 113.219 |
|  | rs76865030 | G | A | 3 | 89286760 | 89237610 | 2.89E-04 | 136.316 |
|  | rs76951538 | TT | AT | 3 | 103402561 | 103683717 | 2.22E-04 | 104.816 |
|  | rs77041475 | G | A | 3 | 95766490 | 96047646 | 2.52E-04 | 119.154 |
|  | rs77428067 | A | C | 3 | 100560285 | 100841441 | 8.58E-05 | 40.500 |
|  | rs77462412 | G | A | 3 | 89034908 | 88985758 | 1.83E-04 | 86.339 |
|  | rs77576857 | A | G | 3 | 102983425 | 103264581 | 2.99E-04 | 141.295 |
|  | rs77691713 | G | A | 3 | 104645519 | 104926675 | 1.19E-04 | 56.287 |
|  | rs77837460 | A | G | 3 | 103456239 | 103737395 | 6.65E-05 | 31.391 |
|  | rs77884189 | A | T | 3 | 88496903 | 88447753 | 1.84E-04 | 86.850 |
|  | rs78346902 | T | G | 3 | 100125307 | 100406463 | 1.28E-04 | 60.316 |
|  | rs78415552 | A | G | 3 | 106606098 | 106887251 | 8.42E-05 | 39.756 |
|  | rs78456010 | C | A | 3 | 107828707 | 108109860 | 6.33E-05 | 29.872 |
|  | rs78761191 | A | C | 3 | 105299699 | 105580855 | 1.60E-04 | 75.726 |
|  | rs79207798 | G | C | 3 | 109101670 | 109382823 | 7.08E-05 | 33.443 |
|  | rs79223210 | G | A | 3 | 95419620 | 95700776 | 3.42E-04 | 161.568 |
|  | rs79254103 | C | T | 3 | 100464565 | 100745721 | 2.07E-04 | 97.739 |
|  | rs79380379 | T | C | 3 | 97783680 | 98064836 | 2.48E-04 | 117.356 |
|  | rs79619962 | A | C | 3 | 111567626 | 111848779 | 7.91E-05 | 37.336 |
|  | rs79674527 | G | A | 3 | 82127493 | 82078342 | 1.64E-04 | 77.645 |
|  | rs79897514 | G | C | 3 | 94345645 | 94626801 | 2.35E-04 | 110.877 |
|  | rs79906866 | T | G | 3 | 99742628 | 100023784 | 1.87E-04 | 88.408 |
|  | rs80217925 | C | G | 3 | 76551066 | 76501915 | 1.98E-04 | 93.545 |
|  | rs80352472 | A | T | 3 | 82217419 | 82168268 | 1.15E-04 | 54.381 |
|  | rs9810227 | G | T | 3 | 101219385 | 101500541 | 2.40E-04 | 113.513 |
|  | rs9836217 | G | A | 3 | 86959091 | 86909941 | 2.96E-04 | 139.598 |
|  | rs9850950 | A | G | 3 | 98156336 | 98437492 | 4.76E-04 | 224.966 |
|  | rs1264708 | G | A | 6 | 30057154 | 30089377 | 9.94E-05 | 46.922 |
|  | rs3130162 | C | G | 6 | 33126064 | 33158287 | 7.62E-05 | 35.961 |
|  | rs3130975 | T | C | 6 | 31081838 | 31114061 | 6.35E-05 | 29.975 |
|  | rs550478926 | G | C | 9 | 117085186 | 114322906 | 9.04E-05 | 42.679 |
|  | rs139117241 | T | C | 10 | 27907547 | 27618618 | 7.07E-05 | 33.365 |
|  | rs12436465 | C | T | 14 | 65806877 | 65340159 | 1.29E-04 | 60.825 |
|  | rs11667325 | A | G | 19 | 52301063 | 51797810 | 7.06E-05 | 33.321 |
|  | rs28873836 | C | G | 19 | 52314655 | 51811402 | 4.56E-04 | 215.184 |
| CHL1 | rs112600426 | G | A | 3 | 2616767 | 2575083 | 1.03E-04 | 48.621 |
|  | rs113069256 | G | T | 3 | 771406 | 729722 | 8.54E-05 | 40.309 |
|  | rs11918944 | G | A | 3 | 98675416 | 98956572 | 1.34E-04 | 63.196 |
|  | rs13077895 | G | A | 3 | 93078 | 51395 | 1.50E-03 | 707.685 |
|  | rs140649637 | T | C | 3 | 1148736 | 1107052 | 7.18E-05 | 33.896 |
|  | rs147994625 | A | G | 3 | 427372 | 385689 | 3.67E-04 | 173.319 |
|  | rs1523906 | T | G | 3 | 838233 | 796550 | 1.24E-04 | 58.745 |
|  | rs17212700 | C | T | 3 | 1345648 | 1303964 | 7.29E-05 | 34.409 |
|  | rs17334011 | TT | TA | 3 | 317688 | 276004 | 4.87E-04 | 230.263 |
|  | rs2454673 | A | G | 3 | 98621119 | 98902275 | 3.85E-04 | 181.882 |
|  | rs28799988 | T | C | 3 | 1607813 | 1566129 | 9.52E-05 | 44.977 |
|  | rs461791 | G | A | 3 | 321327 | 279644 | 2.50E-04 | 118.220 |
|  | rs6442833 | G | A | 3 | 451407 | 409724 | 9.39E-05 | 44.352 |
|  | rs6786962 | G | A | 3 | 5344777 | 5303092 | 6.32E-05 | 29.823 |
|  | rs6806211 | CTCTC | CTCTA | 3 | 311214 | 269527 | 1.94E-04 | 91.443 |
|  | rs72991460 | T | C | 3 | 672186 | 630502 | 6.57E-05 | 31.012 |
|  | rs73011252 | T | C | 3 | 2054861 | 2013177 | 6.34E-05 | 29.945 |
|  | rs75740142 | C | T | 3 | 361660 | 319977 | 2.14E-04 | 101.087 |
|  | rs76756609 | GATAA | GATAG | 3 | 1105964 | 1064276 | 9.80E-05 | 46.255 |
|  | rs9817484 | TATTTATTT | TAATTATTT | 3 | 387150 | 345465 | 1.49E-04 | 70.590 |
|  | rs9829992 | A | G | 3 | 228852 | 187169 | 1.01E-04 | 47.562 |
|  | rs9847822 | C | A | 3 | 428129 | 386446 | 3.39E-04 | 160.035 |
|  | rs12288924 | A | G | 11 | 126286010 | 126416115 | 1.21E-04 | 56.957 |
|  | rs2066985 | T | G | 11 | 126251286 | 126381391 | 2.07E-04 | 97.748 |
|  | rs2057417 | G | A | 13 | 50058867 | 49484731 | 6.60E-05 | 31.180 |
|  | rs12945886 | G | A | 17 | 7073014 | 7169695 | 1.19E-04 | 56.059 |
|  | rs7253490 | A | C | 19 | 22293706 | 22110904 | 7.38E-05 | 34.855 |
| TXNDC5 | rs1043784 | C | T | 6 | 7881931 | 7881698 | 4.70E-04 | 222.197 |
|  | rs112061778 | T | C | 6 | 6686642 | 6686409 | 1.21E-04 | 57.042 |
|  | rs113432335 | A | G | 6 | 7304048 | 7303815 | 2.06E-04 | 97.273 |
|  | rs114568154 | T | A | 6 | 9131202 | 9130969 | 1.13E-04 | 53.437 |
|  | rs115447463 | A | T | 6 | 10804272 | 10804039 | 6.90E-05 | 32.565 |
|  | rs116160783 | A | G | 6 | 8674783 | 8674550 | 1.46E-04 | 68.904 |
|  | rs116502862 | T | C | 6 | 11914536 | 11914303 | 9.78E-05 | 46.181 |
|  | rs116827259 | A | T | 6 | 12682844 | 12682612 | 9.93E-05 | 46.889 |
|  | rs13209436 | T | C | 6 | 8133492 | 8133259 | 1.15E-04 | 54.193 |
|  | rs138493941 | A | G | 6 | 6543860 | 6543627 | 7.79E-04 | 368.265 |
|  | rs141361442 | T | C | 6 | 6338173 | 6337940 | 6.63E-05 | 31.323 |
|  | rs147067275 | A | C | 6 | 7822979 | 7822746 | 2.20E-04 | 103.845 |
|  | rs148218954 | A | G | 6 | 7508624 | 7508391 | 8.01E-05 | 37.847 |
|  | rs148841592 | TAAGTG | GAAGTG | 6 | 5313971 | 5313738 | 6.64E-05 | 31.337 |
|  | rs149679123 | C | T | 6 | 7947075 | 7946842 | 2.26E-04 | 106.628 |
|  | rs149742777 | A | T | 6 | 7909820 | 7909587 | 5.73E-04 | 270.789 |
|  | rs17295665 | A | C | 6 | 5931065 | 5930832 | 8.48E-05 | 40.064 |
|  | rs17339636 | A | G | 6 | 7936441 | 7936208 | 1.07E-04 | 50.482 |
|  | rs190713039 | T | C | 6 | 7426128 | 7425895 | 1.32E-03 | 623.659 |
|  | rs3033968 | AAA | AAC | 6 | 8588435 | 8588200 | 1.02E-04 | 48.190 |
|  | rs557363657 | A | G | 6 | 8962742 | 8962509 | 1.57E-04 | 74.353 |
|  | rs62389984 | C | G | 6 | 7730264 | 7730031 | 1.03E-04 | 48.807 |
|  | rs6928267 | GGAAGAAGAAGAAGAAGAAGAAGAAGAAGAAGAAGAAGAAGAAGAAGAAGAG | GGAAGAAGAAGAAGAAGAAGAAGAAGAAGAAGAAGAAGAAGAAGAAGAAGAC | 6 | 16100153 | 16099871 | 8.92E-05 | 42.103 |
|  | rs71559141 | G | A | 6 | 6941686 | 6941453 | 1.65E-04 | 77.693 |
|  | rs77186833 | T | C | 6 | 9912068 | 9911835 | 7.47E-05 | 35.273 |
|  | rs6796 | C | T | 7 | 6502367 | 6462736 | 7.16E-05 | 33.833 |
|  | rs78740585 | ACTG | GCTG | 7 | 150944302 | 151247216 | 1.37E-04 | 64.482 |
|  | rs7903703 | TC | TT | 10 | 28935149 | 28646219 | 6.67E-05 | 31.476 |
|  | rs28929474 | T | C | 14 | 94844947 | 94378610 | 1.73E-04 | 81.512 |
|  | rs56238200 | A | G | 17 | 80356678 | 82398802 | 4.81E-04 | 227.203 |
|  | rs8078222 | C | T | 17 | 7434239 | 7530922 | 8.61E-05 | 40.658 |
| SNPs used as genetic instruments for the backward MR analysis. Included are the effect and other alleles, chromosome location, base-pair positions (GRCh37 and GRCh38), * R² (proportion of variance in the protein explained by each SNP), and ♰ F-statistics (instrument strength). Abbreviations: SNP, single nucleotide polymorphism; R², variance explained; F, F-statistic. | | | | | | | | |
|  |  |  |  |  |  |  |  |  |

| **Table S16. Sensitivity Analysis of LTL with Incident AAA by Additional Adjustment for White Blood Cell Count in ARIC (1987-2019)** | | | | |  |
| --- | --- | --- | --- | --- | --- |
|  | **HR (95% CI)** | **P value** | **N Events / Total Participants †** | **P trend** |  |
| Continuous LTL per unit increment | 0.891 (0.811, 0.979) | 0.002 | 499/10,161 |  |  |
| LTL quantile group 1 | Ref |  | 159/2,444 | 0.02 |  |
| LTL quantile group 2 | 0.839 (0.665, 1.060) | 0.14 | 130/2,511 |  |  |
| LTL quantile group 3 | 0.765 (0.601, 0.975) | 0.03 | 114/2,581 |  |  |
| LTL quantile group 4 | 0.766 (0.592, 0.991) | 0.043 | 96/2,625 |  |  |
| Hazard ratios (HR) and 95% confidence intervals (CI) for the association between leukocyte telomere length (LTL) and incident abdominal aortic aneurysm (AAA), adjusted for white blood cell count, age, sex, body mass index (BMI), race, center, diabetes, hypertension, smoking status, eGFR, and LTL sample visit. † Number of AAA events / total number of participants. | | | | |  |
|  |  |  |  |  |  |
|  |  |  |  |  |  |

| **Table S17. Associations Between the LTL-Associated Proteins and AAA Incidence in ARIC (1987-2019)*** | | | | |
| --- | --- | --- | --- | --- |
| **Protein ID** | **HR (95% CI)** | **P** | **Gene Symbol** | **Target Full Name** |
| 3651_50 | 0.957 (0.871, 1.051) | 0.357 | KDR | Vascular endothelial growth factor receptor 2 |
| 2665_26 | 1.007 (0.912, 1.112) | 0.886 | TNFRSF17 | Tumor necrosis factor receptor superfamily member 17 |
| 4374_45 | 1.238 (1.115, 1.375) | <0.001 | GDF15 | Growth/differentiation factor 15 |
| 6947_4 | 0.943 (0.864, 1.029) | 0.187 | ST3GAL6 | Type 2 lactosamine alpha-2,3-sialyltransferase |
| 8958_51 | 0.993 (0.902, 1.093) | 0.881 | CHL1 | Neural cell adhesion molecule L1-like protein |
| 4990_87 | 1.000 (0.915, 1.093) | 0.996 | GP1Bα | Platelet glycoprotein Ib alpha chain |
| *Hazard ratios (HR) and 95% confidence intervals (CI) for the associations between proteins identified as causally associated with leukocyte telomere length (LTL) and the risk of incident abdominal aortic aneurysm (AAA). Models were adjusted for age, sex, race, field center, body mass index (BMI), prevalent diabetes and hypertension, smoking status, and estimated glomerular filtration rate (eGFR). | | | | |

|  | **Table S18. Mediation Analysis of the Association Between LTL and AAA through Proteins as Mediators in ARIC**♰ | | | | | | | | |
| --- | --- | --- | --- | --- | --- | --- | --- | --- | --- |
|  |  |  | **Decomposition of Mediation Effects** | | | | | **Protein-AAA Association Results*** | |
| **Protein ID** | **Gene Symbol** | **Protein Name** | **HR for Direct Effect (95% CI)** | **HR for Indirect Effect (95% CI)** | **HR for Total Effect (95%CI)** | **Proportion Mediated (95% CI)** | **P-value for Proportion Mediated** | **HR for AAA (95% CI)** | **P value** |
| 4374_45 | GDF15 | Growth/ differentiation factor 15 | 0.886  (0.806, 0.973) | 0.982  (0.970, 0.993) | 0.869  (0.790, 0.956) | 0.124  (0.013, 0.235) | 0.028 | 1.238  (1.115, 1.375) | < 0.001 |
| 3651_50 | KDR | Vascular endothelial growth factor receptor 2 | 0.876 (0.799, 0.961) | 0.999 (0.991, 1.007) | 0.875 (0.797, 0.961) | 0.008 (-0.047, 0.063) | 0.777 | 0.957 (0.871, 1.051) | p=0.357 |
| 2665_26 | TNFRSF17 | Tumor necrosis factor receptor superfamily member 17 | 0.874 (0.796, 0.959) | 0.998 (0.991, 1.005) | 0.872 (0.795, 0.957) | 0.015 (-0.036, 0.066) | 0.557 | 1.007 (0.912, 1.112) | p=0.886 |
| 6947_4 | ST3GAL6 | Type 2 lactosamine alpha-2,3-sialyltransferase | 0.873 (0.795, 0.958) | 0.998 (0.993, 1.003) | 0.871 (0.793, 0.956) | 0.016 (-0.020, 0.052) | 0.380 | 0.943 (0.864, 1.029) | p=0.187 |
| 8958_51 | CHL1 | Neural cell adhesion molecule L1-like protein | 0.873 (0.795, 0.958) | 1.000 (0.992, 1.007) | 0.872 (0.795, 0.957) | 0.003 (-0.050, 0.056) | 0.915 | 0.993 (0.902, 1.093) | p=0.881 |
| 4990_87 | GP1Bα | Platelet glycoprotein Ib alpha chain | 0.874 (0.796, 0.960) | 0.997 (0.986, 1.008) | 0.872 (0.794, 0.956) | 0.020 (-0.055, 0.095) | 0.603 | 1.000 (0.915, 1.093) | p=0.996 |
| ♰All effect decomposition from the mediation analysis was on the hazard ratio scale via the regression-based approach.  Adjusted for corresponding LTL sample visit, age, sex, race, field center, eGFR, BMI, prevalent diabetes and hypertension, and smoking status.  *The GDF15-AAA association analysis adjusted for age, sex, race, field center, eGFR, BMI, prevalent diabetes and hypertension, and smoking status. | | | | | | | | | |

**Table S19. Spearman Correlation for Protein Measures by SomaScan vs Other Assays in ARIC for Selected Top LTL Proteins**

| **Aptamer ID** | **Olink ID** | **Gene Name** | **Uniprot** | **Spearman r* (N=102)** | **CVBA SomaScan (N=115)** | **CVBA Olink (N=105)** |
| --- | --- | --- | --- | --- | --- | --- |
| 4374_45 | Roche assay | GDF15 | Q99988 | Soma vs Roche 0.936 | 5.7% | CV Roche 5.3% |
| 4990_87 | OID45365 | GP1Bα | P07359 | 0.746 | 6.7% | 5.6% |
| 11212_7 | OID45003 | TXNDC5 | Q8NBS9 | 0.736 | 3.6% | 15.8% |
| 5947_90 | OID44322 | THPO | P40225 | 0.437 | 7.8% | 6.1% |
| 2665_26 | OID45282 | TNFRSF17 | Q02223 | 0.771 | 4.6% | 23.0% |
| 16322_10 | OID44799 | MZB1 | Q8WU39 | 0.849 | 7.6% | 8.9% |
| 10612_18 | OID41632 | PLOD3 | O60568 | -0.054 | 5.0% | 8.8%♰ |
| 8958_51 | OID45336 | CHL1 | O00533 | 0.765 | 5.7% | 8.1% |
| 10702_1 | OID43551 | COL28A1 | Q2UY09 | -0.037 | 5.3% | 14.2% |
| 8275_31 | OID45216 | PEAR1 | Q5VY43 | 0.578 | 4.5% | 13.8% |
| 3651_50 | OID44727 | KDR | P35968 | 0.609 | 5.7% | 6.5% |
| 7185_29 | OID44691 | GP5 | P40197 | 0.662 | 4.7% | 16.5% |
| *Spearman correlation for protein measures between SomaScan 11k and Olink Explore HT platforms unless otherwise stated.  CVBA, coefficient of variation by Bland-Altman method; SomaScan: SomaScan 11 k assay; Olink, Olink Explore HT assay  ♰ CVBA for PLOD3 was calculated after setting all values below the limit of detection (LOD; 0.106 NPX) to LOD/2. | | | | | | |
